# Evaluation of public cancer datasets and signatures identifies TP53 mutant signatures with robust prognostic and predictive value

**DOI:** 10.1186/s12885-015-1102-7

**Published:** 2015-03-26

**Authors:** Brian David Lehmann, Yan Ding, Daniel Joseph Viox, Ming Jiang, Yi Zheng, Wang Liao, Xi Chen, Wei Xiang, Yajun Yi

**Affiliations:** 1 Department of Biochemistry, Vanderbilt University, Nashville, TN USA; 2 Department of Biostatistics, Vanderbilt University, Nashville, TN USA; 3 Division of Epidemiology, Vanderbilt University, Nashville, TN USA; 4 Vanderbilt Ingram Cancer Center, Vanderbilt University, Nashville, TN USA; 5 Department of Medicine, Vanderbilt University, Nashville, TN USA; 6 Department of Pediatrics, Maternal and Child Health Care Hospital of Hainan Province, Haikou, China; 7 Feinberg School of Medicine, Northwestern University, Chicago, IL USA; 8 Laboratory of Nuclear Receptors and Cancer Research, Center for Basic Medical Research, Nantong University School of Medicine, Nantong, Jiangsu China; 9 Pediatric Surgery Department, Qilu Hospital of Shandong University, Jinan, Shangdong China; 10 Department of Cardiovascular Disease, Hainan General Hospital, Haikou, Hainan China; 11 Department of Dermatology, Hainan General Hospital, Haikou, Hainan China; 12 Division of Genetic Medicine, 536A Light Hall, Vanderbilt University, 2215 Garland Avenue, Nashville, TN 37232-0275 USA

**Keywords:** Breast cancer, Gene expression profiles, Signatures, Meta-analysis, Prognosis, HER2− breast cancer, Chemosensitivity

## Abstract

**Background:**

Systematic analysis of cancer gene-expression patterns using high-throughput transcriptional profiling technologies has led to the discovery and publication of hundreds of gene-expression signatures. However, few public signature values have been cross-validated over multiple studies for the prediction of cancer prognosis and chemosensitivity in the neoadjuvant setting.

**Methods:**

To analyze the prognostic and predictive values of publicly available signatures, we have implemented a systematic method for high-throughput and efficient validation of a large number of datasets and gene-expression signatures. Using this method, we performed a meta-analysis including 351 publicly available signatures, 37,000 random signatures, and 31 breast cancer datasets. Survival analyses and pathologic responses were used to assess prediction of prognosis, chemoresponsiveness, and chemo-drug sensitivity.

**Results:**

Among 31 breast cancer datasets and 351 public signatures, we identified 22 validation datasets, two robust prognostic signatures (BRmet50 and PMID18271932Sig33) in breast cancer and one signature (PMID20813035Sig137) specific for prognosis prediction in patients with ER-negative tumors. The 22 validation datasets demonstrated enhanced ability to distinguish cancer gene profiles from random gene profiles. Both prognostic signatures are composed of genes associated with TP53 mutations and were able to stratify the good and poor prognostic groups successfully in 82%and 68% of the 22 validation datasets, respectively. We then assessed the abilities of the two signatures to predict treatment responses of breast cancer patients treated with commonly used chemotherapeutic regimens. Both BRmet50 and PMID18271932Sig33 retrospectively identified those patients with an insensitive response to neoadjuvant chemotherapy (mean positive predictive values 85%-88%). Among those patients predicted to be treatment sensitive, distant relapse-free survival (DRFS) was improved (negative predictive values 87%-88%). BRmet50 was further shown to prospectively predict taxane-anthracycline sensitivity in patients with HER2-negative (HER2-) breast cancer.

**Conclusions:**

We have developed and applied a high-throughput screening method for public cancer signature validation. Using this method, we identified appropriate datasets for cross-validation and two robust signatures that differentiate TP53 mutation status and have prognostic and predictive value for breast cancer patients.

**Electronic supplementary material:**

The online version of this article (doi:10.1186/s12885-015-1102-7) contains supplementary material, which is available to authorized users.

## Background

Hundreds of transcriptional profiles have been identified to report useful information in the field of predictive oncology such as the likelihood of cancer progression [1,2], cancer subtying [3], treatment outcomes [4], and drug sensitivities [5-7].

Beyond its clinical utility, a signature can also provide candidate genes for gene function analysis [8] and serve as a marker of specific mechanisms, pathways [9], mutations (e.g., TP53 mutation) [10], and various biological states such as wound healing [11,12], hypoxia [13,14], and tumor stroma [15]. Utilizing a common translational strategy, these studies often demonstrate that these signatures have a significant association with clinical outcome in cancer patients.

There are at least several hundred cancer signatures and dozens of validation datasets that have been reported in the scientific literature [7,16]. However, the overproducing in signature discovery relative to signature validation presents an exceptional challenge to their use. It is evident that the majority of transcriptional gene signature studies published to date do not progress beyond the discovery phase. The validation phase of gene-expression signatures is very time-consuming and costly because it requires either multiple retrospective studies with large sample sizes or prospective clinical trials. For these reasons, there has been no systematic method for assessing the prognostic and predictive value of these publicly available signatures across multiple cancer patient populations.

Because there are no standard criteria to guide the selection of test datasets, most studies focus on a few well-known datasets (e.g., NKI295 [17]). In fact, few signatures have been externally validated using more than five datasets. Not surprisingly, this validation method has inevitable limitations in terms of statistical power and sample selection bias. A common weakness of this approach is its lack of consistency and reproducibility [18-22] resulting in the false positive paradox whereby falsely significant gene-expression signatures are identified more frequently than truly significant ones [16].

The identification of robust predictive signatures through meta-analysis of publicly available gene-expression signatures on a large scale still represents an underexploited opportunity.

To avoid overtreatment – subjected to morbidity from cytotoxic chemotherapy for negligible benefit, an important problem inherent to neoadjuvant (preoperative) chemotherapy is the identification of those patients likely to be sensitive to neoadjuvant chemotherapy from those likely to be insensitive. One strategy for doing so is the use of prognostic and predictive biomarkers. The chemotherapeutic response to neoadjuvant chemotherapy measured at the time of definitive surgery is usually dichotomized as pathologic complete response (pCR; e.g., absence of invasive breast cancer in both the primary tumor bed and regional lymph nodes) and residual disease (RD). It can also be categorized into a semi-quantitative, four-tiered response score, (e.g., residual cancer burden (RCB-0/I to IV)).

Patients with breast cancer that achieve pCR or RCB-0/I following neoadjuvant chemotherapy often have an excellent probability of long-term survival (>3 years relapse-free), while patients with RD often have a higher probability of early relapse within 3 years [23-25]. Thus, pCR or RD after neoadjuvant chemotherapy provides a clinical model for validation of gene-expression signature prediction.

There are very few molecular tests developed specifically to predict the probability of both short-term pCR/RD/RCB following neoadjuvant chemotherapy and long-term survival [26-28]. Very few studies in the discovery phase have both gene-expression profiles and treatment responses available that can be used to develop signatures directly related to treatment responses. In the validation phase, large and logistically challenging clinical trials may take decades to accumulate sufficient events for a useful analysis. An alternative and more rapid approach is to evaluate the predictive value of a prognostic marker for chemosensitivity in the neoadjuvant setting [4,29,30].

To analyze the prognostic and predictive value of publicly available signatures, we performed a large-scale meta-analysis of cancer signatures, including 351 publicly available signatures and 31 validation datasets in breast cancer.

Our three primary objectives were: (1) to systematically evaluate the performance of public signatures and validation datasets in the prediction of breast cancer prognosis, (2) to analyze the association between predicted and actual treatment responses (pCR/RD/DRFS), and (3) to assess the predictive value of a signature for taxane-anthracycline sensitivity in patients with human epidermal growth factor receptor 2 negative (HER2-) breast cancer.

## Methods

### Publicly available signatures

In the past two decades, a large number of gene-expression signatures have been reported and tested on an individual basis. This abundance of signatures has provided us with the unique opportunity to perform a large-scale meta-analysis of signatures for cancer prognosis.

We collected 351 gene-expression signatures from a total of 206 studies (Additional file 1: Table S1). Each study has one or more signatures generated using its authors’ own study designs and sample phenotypes. 95% (333) of the collected signatures are derived from cancer-related studies, with 73% (257) representing breast cancer signatures. The remaining 5% (18) are from other (non-cancer) diseases. Most breast cancer signature phenotypes are related to cancer relapse or poor prognosis, including tumor size, nodal involvement, grade, lymphovascular invasion, TP53 status, BRCA1 mutation, BRCA2 mutation, estrogen receptor (ER) status, and HER2 status (Additional file 1: Table S1).

### Validation datasets

In order to use survival analysis to validate the public signatures across multiple test datasets, we collected 31 breast cancer datasets containing both clinical survival data and gene-expression data. These datasets were derived from published human cancer studies, the Gene Expression Omnibus (GEO) provided by the National Center for Biotechnology Information (NCBI) [31], and The Cancer Genome Atlas (TCGA) (Additional file 1: Table S2).

Each test dataset includes gene-expression values interrogated at the genome level by over 20,000 gene probes (“Total probe number” in Additional file 1: Table S2) and clinical endpoints (outcome events and survival time). The primary clinical endpoints in the validation datasets include disease-specific survival (DSS), disease-free survival (DFS), distant metastasis-free survival (DMFS), overall survival (OS), relapse-free survival (RFS), and distant relapse-free survival (DRFS). These publicly available datasets meet common criteria for survival analysis [32]. The average follow-up length was 10 years across the 31 datasets.

Among the 31 test datasets, two datasets (GSE25055 and GSE25065) have special tumor samples from patients with HER2- breast cancer treated with neoadjuvant chemotherapy (taxane-anthracycline) [6].

GSE25055 includes a cohort of 310 samples with an average pathologic response rate of 25% (pCR), and GSE25065 has a cohort of 198 patients with an average pathologic response rate of 30% (pCR or RCB-I). Both datasets have a median follow-up of 3 years, and an overall 3-year DRFS of 79% [6].

### Translational study design for drug sensitivity prediction

Sequential taxane and anthracycline-based drugs are common regimens for newly diagnosed ERBB2 (HER2 or HER2/neu)-negative breast cancer patients. Data from two studies, GSE25055 and GSE25065, in which patients received this preoperative chemotherapy regimen and the pathologic responses were recorded following surgery were used to test the predictive ability of the gene-expression signatures [6].

In order to construct a reference drug-sensitivity signature for individual prospective prediction, we used the Sanger Genomics of Drug Sensitivity (GDS) dataset containing hundreds of annotated human cancer cell lines [33]. These cancer cell lines have been characterized using gene-expression profiling, and their sensitivities to hundreds of anti-cancer drugs, including taxane-anthracycline, have been assessed. Each cancer cell line has two sets of data-chemosensitivity data and transcriptional profiles from microarrays. By linking the drug activity to the gene-expression profiles in cancer cell lines, the Sanger GDS dataset has facilitated the identification of several genomic markers of drug sensitivity in cancer cells [33]. The taxane-anthracycline drug sensitivity in the breast cancer cell line model was measured as the drug concentration leading to 50% growth inhibition of cancer cells compared to controls (IC_50_).

We identified 13 HER2- breast cancer cell lines that are sensitive to anthracycline and/or taxane treatment (log(IC_50_) < −1). BRmet50 and PMID18271932Sig33 gene-expression values were retrieved to build two taxane-anthracycline-sensitive reference profiles called centroids defined as the average of each predictor’s gene-expression values across the 13 drug-sensitive cell lines [3]. Consequently, taxane-anthracycline sensitivity prediction was achieved by correlating the expression profile of each patient sample with the centroid computed by the PAM algorithm [3]. Briefly, we calculated the Spearman’s rank correlation between each patient profile and the centroids. A patient was predicted to have a sensitive taxane-anthracycline response if the correlation coefficient was larger than 0.35. Otherwise, the patient was considered to be insensitive or resistant to taxane-anthracycline.

Two types of treatment responses were used in the translational study including short-term pathological responses (pCR, RD, or RCB) and long-term DRFS. The first objective of the study was the prediction of pathologic response. We examined whether actual pathologic responses were associated with predicted responses (sensitive and insensitive). The second objective was prediction of long-term treatment outcomes by determining whether patients predicted to be treatment-sensitive had improved DRFS.

### TCGA gene expression and TP53 mutational analysis

TP53 mutation status and Z-score normalized RNA-seq expression values (V2 RSEM) were obtained from cBioPortal [34] for genes in the BRmet50 and PMID18271932Sig33 signatures. Unsupervised hierarchical clustering (Euclidean, complete) was performed on samples containing both RNA-seq expression values and TP53 mutation status was visualized with R package ‘heatmap.2’ (version 3.1.0).

### Statistical analysis

**Figure 1:**
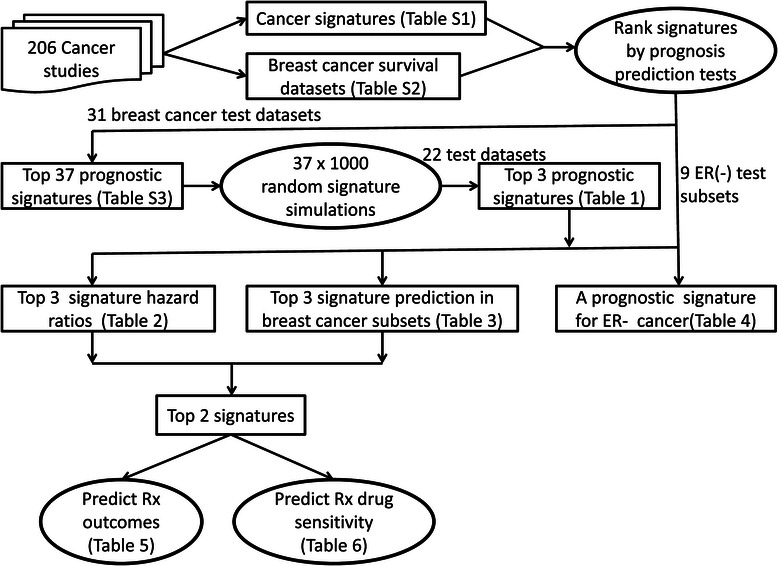
**Overview of meta-analysis of signatures in cancer.** We have performed a large-scale meta-analysis of cancer signatures, including 351 publicly available cancer signatures (Additional file 1: Table S1) and 31 breast cancer test datasets (Additional file 1: Table S2). Based on the predictive performance of each signature in 31 breast cancer test datasets and 9 ER-negative (ER-) subsets, we first identified our top 37 signature candidates (Additional file 1: Table S3) for breast cancer prognosis prediction and one signature for prognosis prediction in ER- subsets (Table 4). Using 37,000 random signature permutation tests and 22 verified test datasets, we narrowed down our top 37 candidates to our top three signatures (Table 1). Next, the top three signatures were further evaluated by uni-/multi-variate hazard ratio tests (Table 2) and breast cancer subsets (Table 3), and two of the three were confirmed as valid and independent prognostic signatures. Finally, we examined the ability of the top two signatures to predict chemotherapy outcomes in breast cancer patients (Table 5) and taxane-anthracycline sensitivity in patients with HER2 - beast cancer (Table 6).

Our statistical approaches, as illustrated in Figure 1, assessed the ability of 351 public signatures and 37,000 random control signatures to serve as survival time predictors (Additional file 1: Table S3, Table 1). First, hierarchical clustering of each signature gene profile in each test dataset was performed and visualized using the open-source desktop program (version 1.5.0.Gbeta) developed at Vanderbilt University. Spearman rank correlation was used to measure the similarities in gene-expression profiles among patient samples.

**Table 1:** **Top 3 signatures for prognosis prediction**

Signature	Significant P value %	Adjusted median P value	Signature description
**BRmet50**	82%	0.013	Meta-signature for cancer metastasis
**PMID18271932Sig33**	64%	0.014	Predictor gene set for TP53 status
**PMID16505416Sig822**	68%	0.015	Poor prognosis signatures for ER+ and PR+ breast cancer

**Figure 2:**
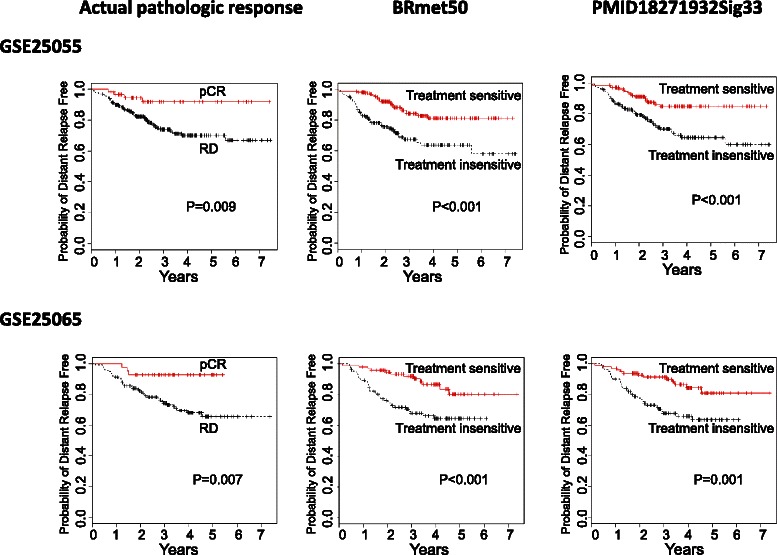
**Kaplan-Meier estimates of distant relapse–free survival analyses of three predictors.** 508 patients with HER2- breast cancer from two independent datasets (GSE25055 and GSE25065) were stratified into two groups according to the gene-expression profiles of two genomic predictors (BRmet50 and PMID18271932) and pathologic response (after treatment) such as pathologic complete response (pCR) and residual disease (RD). In each survival plot, two types of distant relapse-free survival retrospectively determined the two genomic predictor group names (treatment-sensitive and treatment insensitive) and were compared: pCR or treatment-sensitive group (solid red line) and RD or treatment-insensitive group (dashed black line). The distant relapse-free time in years is displayed on the x-axis, and the y-axis shows the probability of distant relapse-free survival. The P values indicate the statistical significance of survival time differences between the two groups.

**Figure 3:**
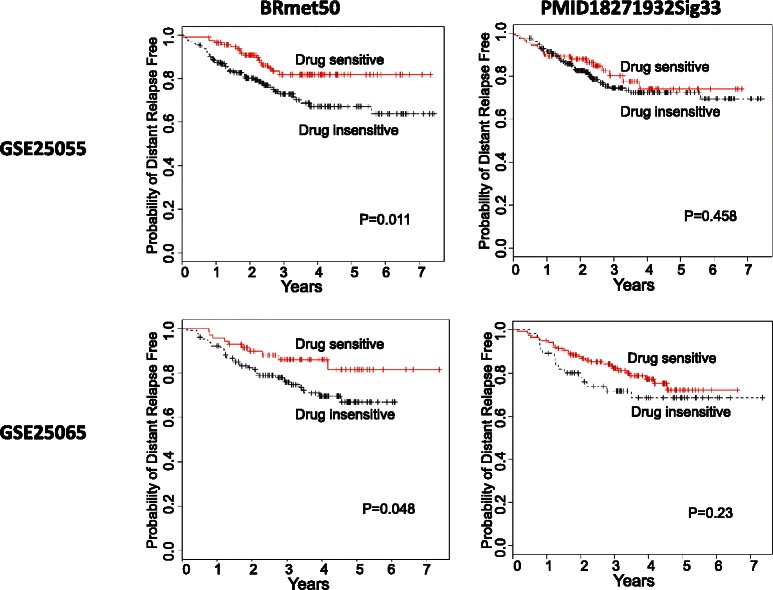
**Kaplan-Meier estimates of distant relapse–free survival analyses of two predictors of taxane-anthracycline sensitivity.** 508 patients with HER2- breast cancer from two independent datasets (GSE25055 and GSE25065) were stratified into two groups according to the taxane-anthracycline centroid correlation. In each survival plot, two types of distant relapse-free survival were prospectively determined before taxane-anthracycline treatment: drug-sensitive (solid red line) and drug-insensitive (dashed black line). The distant relapse-free time in years is displayed on the x-axis, and the y-axis shows the probability of distant relapse-free survival. The P values indicate the statistical significance of survival time differences between the two groups.

To evaluate various signatures with full datasets and subsets, survival curves were calculated using the Kaplan–Meier method and compared using the log-rank test. The association between each gene signature and survival time was also evaluated using univariate and multivariate Cox proportional hazards models. Unsupervised hierarchical clustering based on average linkage was performed to group the patient samples. The group assignments for the patient samples were determined for each dataset based on the first bifurcation of the clustering sample dendrogram [35]. Using disease outcomes, Kaplan-Meier curves for the two groups were compared. For graphical representation, Kaplan-Meier curves of survival probability were plotted (Figures 2 and 3). Log-rank tests and c-index measurements were conducted for the two groups’ survival difference. The Cox proportional hazards model was applied to some datasets for both univariate and multivariate survival analyses (Tables 2, 3, and 4). P values reported are two-sided. Various disease outcomes (e.g., relapse, distant metastasis) were used as clinical endpoints (Tables 2 and 3). The estimated hazard ratio (HR), its 95% confidence interval (CI), and the P value allowed us to directly compare the performances of different signatures. All these analyses were carried out with the open-source R software, version 2.14.1.

**Table 2:** **Comparison of top 3 signatures by hazard ratio model**

		BR1042	BR1095	BR1128	BR1141	BRGSE7390
Signatures	Clinical	RFS	DFS	DFS	RFS	RFS
**BRmet50**	**c-index**	0.657	0.605	0.637	0.607	0.633
	**P value**	0.002	<0.001	<0.001	<0.001	0.001
**Log-rank test**	**P value**	0.002	<0.001	<0.001	<0.001	0.001
**Univariate**	**HR**	2.8(1.4-5.5)	2.2(1.4-3.3)	2.8(1.5-4.9)	2.2(1.5-3.3)	2.3(1.4-3.8)
	**P value**	0.002	<0.001	<0.001	<0.001	0.001
**Multivariate**	**HR**	3.2(1.4-7.5)	1.8(1.1-2.9)	2.0(1.0-3.9)	2.3(1.4-3.6)	3.0(1.6-5.9)
	**P value**	0.006	0.021	0.035	0.001	0.001
**PMID**	**c-index**	0.616	0.622	0.665	0.636	0.608
**18271932**						
**Sig33**						
	**P value**	0.030	<0.001	<0.001	<0.001	0.010
**Log-rank test**	**P value**	0.030	<0.001	<0.001	<0.001	0.010
**Univariate**	**HR**	2.0(1.1-3.8)	2.5(1.6 -3.9)	3.5(2.0-6.4)	2.4(1.6-3.6)	1.9(1.2-3.3)
	**P value**	0.030	<0.001	<0.001	<0.001	0.010
**Multivariate**	**HR**	1.9(0.8-4.5)	2.0(1.2-3.3)	2.9(1.5-5.6)	2.3(1.5-3.7)	2.7(1.4-5.3)
	**P value**	0.171	0.006	0.002	<0.001	0.004
**PMID**	**c-index**	0.517	0.586	0.638	0.549	0.634
**16505416**						
**Sig822**						
	**P value**	0.698	0.002	<0.001	0.156	0.001
**Log-rank test**	**P value**	0.698	0.002	<0.001	0.156	0.001
**Univariate**	**HR**	1.1( 0.4 -1.7)	1.9(1.3-3.0)	2.8(1.6-4.8)	1.4(0.9-2.1)	2.3(1.4-3.9)
	**P value**	0.698	0.002	0.000	0.155	0.001
**Multivariate**	**HR**	1.3(0.6-2.7)	1.5(0.9-2.7)	2.3(1.2-4.4)	1.5(1.0-2.4)	2.8(1.4-5.4)
	**P value**	0.544	0.138	0.014	0.053	0.003

Multivariate HR adjusted by age, grade, continuous tumor size, LN, ER, NPI1.

**Table 3:** **Hazard ratio risks and log-rank tests in BR1141**

	BRmet50		PMID18271932Sig33		PMID16505416Sig822	
	HR(95% CI)	HR P	HR (95% CI)	HR P	HR (95% CI)	HR P
**Tumor size**						
**T1**	2.6(1.3-5.5)	0.009	2.4(1.1-5.0)	0.019	1.0(0.5-2.1)	0.943
**T2**	1.7(1.0-2.8)	0.044	2.0(1.3-3.3)	0.008	0.7(0.4-1.2)	0.209
**Lymph node involvement**						
**No**	2.3(1.4-3.9)	0.001	2.2(1.3-3.3)	0.003	0.8(0.5-1.4)	0.511
**Yes**	2.0(1.0-4.1)	0.053	2.8(1.4-5.0)	0.004	0.6(0.3-1.4)	0.245
**Tamoxifen treatment**						
**No**	2.6(1.4-5.0)	0.004	2.5(1.3-5.0)	0.007	1.1(0.5-2.0)	0.869
**Yes**	2.2(1.2-3.9)	0.007	2.6(1.4-5.0)	0.001	0.6(0.3-1.0)	0.041
**Differentiation**						
**Good**	2.3(0.6-8.4)	0.196	2.5(0.8-10)	0.131	1.3(0.4-3.8)	0.682
**Intermediate**	2.5(1.5-4.3)	0.001	3.3(2.0-5.0)	<0.001	0.7(0.4-1.2)	0.194
**Poor**	1.4(0.6-3.4)	0.442	1.3(0.6-3.3)	0.554	0.5(0.2-1.1)	0.086
**ER status**						
**Negative**	1.4(0.5-4.0)	0.495	1.7(0.6-5.0)	0.329	0.9(0.3-2.4)	0.782
**Positive**	2.5(1.6-4.0)	<0.001	2.5(1.7-5.0)	<0.001	0.7(0.4-1.1)	0.102

Note: T1 denotes tumor size less than or equal to 2.0 cm, and T2 denotes tumor size larger than 2.0 cm.

HR: Hazard ratio value.

HR P: Hazard ratio P value.

**Table 4:** **A prognostic signature for patients with ER- breast cancer**

ER negative dataset (sample size)	Clinical end points	Log-rank test	Univariate HR		Multivariate HR	
		P value	HR (95% CI low-high)	P value	HR (95% CI low-high)	P value
**BR1141(40)**	**RFS**	0.828	1.1(0.4-3.0)	0.828	NA^#^	NA
**BR2411(69)**	**RFS**	0.590	1.3(0.5-2.9)	0.591	NA	NA
**BRGSE20624(115)**	**RFS**	0.442	1.3(0.5-3.0)	0.602	NA	NA
**BR25055(131)**	**DRFS**	0.406	1.3(0.7-2.5)	0.406	NA	NA
**BR1405(77)**	**RFS**	0.015	2.5(1.2-5.4)	0.015	NA	NA
**BRGSE21653(113)**	**DFS**	0.022	2.1(1.1-3.9)	0.022	2.5(1.1-5.4)	0.023
**BRMetabric**	**DSS**	0.001	2.2(1.4-3.7)	0.001	2.4(1.1-5.2)	0.022
**D22522925(196)**						
**BRMetabric**	**DSS**	0.015	1.6(1.1 -2.5)	0.015	1.7(1.0-2.9)	0.036
**V22522925(244)**						
**BRTCGA(118)**	**OS**	0.003	13.8(1.4-34.6)	0.003	3.2(1.5-5.2)	0.025

Note: Multivariate HR adjusted factors in each test dataset are as follows: BRGSE21653 (grade, PR, HER2 status, ki67), BRMetabricD22522925 and BRMetabricV22522925 (age, menopausal status, grade, size, stage, lymph node positivity, NPI, HER2 status, PAM50 subtype, treatment), BRTCGA (PR, HER2 status, T stage).

#: No adjusted clinical covariates; no mulitvariate HR analysis was performed.

Pathologic response to neoadjuvant chemotherapy was defined as pCR/RD or RCB for evaluation of response prediction. The primary prediction endpoint was DRFS at 3 years (median follow-up for the validation cohort). Predictive performance was assessed by the positive predictive value (PPV), defined as the probability of RD, distant relapse, or death for patients predicted to be treatment-insensitive, and the negative predictive value (NPV), defined as the patient’s probability of pCR/RCB-0/I or improved DRFS (>3 years) for patients predicted to be treatment-sensitive [6].

## Results

### Study overview

To investigate the performance of public cancer signatures, we performed a large-scale meta-analysis (Figure 1) of cancer signatures, including 351 publicly available signatures from 206 studies (Additional file 1: Table S1). Based on the predictive performance of each signature in 31 breast cancer test datasets (Additional file 1: Table S2) and nine estrogen receptor-negative (ER-) subsets, we identified 37 significant signature candidates (Additional file 1: Table S3) capable of robustly predicting breast cancer prognosis as a whole and one signature that predicts prognosis in the ER- setting (Table 4). Using 37,000 random signature permutation tests, we narrowed down our 37 candidate signatures to a top three (Table 1). The top three signatures were further evaluated for their ability to independently predict prognosis by uni-/multi-variate Cox proportional hazards models (Table 2) as well as breast cancer subsets (Table 3). Two of the three were confirmed as valid prognostic signatures. Finally, we examined the top two signatures’ ability to predict chemotherapy outcomes in breast cancer patients (Table 5) and taxane-anthracycline sensitivity in patients with HER2- breast cancer (Table 6).

**Table 5:** **Chemotherapy outcome prediction using prognostic signatures**

Response prediction (n = sample size)	BRmet50	PMID18271932Sig33	pCR	RCB
pCR/RD(n = 993)*				
Mean PPV(SD)	88(6)	88(5)	NA	NA
Mean NPV(SD)	35(4)	35(6)	NA	NA
RCB(n = 417)**				
Mean PPV(SD)	85(1)	86(3)	NA	NA
Mean NPV(SD)	42(1)	41(0.3)	NA	NA
DRFS(n = 508)**				
Mean PPV(SD)	32(0.3)	31(2)	26(0.4)	49(2)
Mean NPV(SD)	88(6)	87(4)	92(0.8)	83(4)

*Five data sets with neoadjuvant chemotherapy records include GSE25055 (anthracycline-taxane ), GSE25065 (anthracycline-taxane ), GDS4057(5-fluorouracil, doxorubicin, and cyclophosphamide), GSE32646 (5-fluorouracil-epirubicin, and cyclophosphamide), GSE41998 (doxorubicin and cyclophosphamide followed by ixabepilone or paclitaxel).

**DRFS: Distant relapse free-survival, 508 samples from two data sets including GSE25055 and GSE25065.

**Table 6:** **Prediction of taxane-anthracycline sensitivity in patients with HER2-negative(HER2-) breast cancer**

	pCR prediction(n = 488)			
Prediction evaluation*	PPV (SD)	NPV (SD)	Sensitivity (SD)	Specificity (SD)
**Unsupervised clustering**				
**BRmet50**	93(14)	34(3)	58(3)	83(0.6)
**PMID18271932Sig33**	92(3)	32(5)	53(6)	83(6)
**Anthracycline-taxane Centroid**				
**BRmet50**	89(6)	27(0.6)	40(5)	83(6)
**PMID18271932Sig33**	77(4)	20(3)	29(1)	67(0.5)

### Evaluation of public signatures using 31 test datasets identifies signatures with robust prognostic ability

To examine the 351 public signatures and rank their ability to predict breast cancer prognosis, we retrospectively screened them (Additional file 1: Table S1) using 31 test datasets (Additional file 1: Table S2).

To identify gene-expression signatures with robust predictive capacity, we performed 10,881 log-rank tests (351 signatures multiplied by 31 breast cancer test datasets). Signatures that provide true prognostic value should demonstrate statistical significance across multiple datasets. Therefore, we ranked the 351 public signatures by percentage of significant P values in the 31 breast cancer datasets. Those signatures capable of predicting prognosis successfully (P < 0.05) in more than half of the test datasets (i.e., significant P value rate > 50%) were selected for further signature analysis (Additional file 1: Table S3) [32] and dataset validation.

We identified 37 signatures with robust predictive ability. Among these were such signatures as Oncotype DX (PMID18360352Sig21, ranked number 3) [2] and MammaPrint (PMID11823860Sig70, ranked number 35) [36], which had the ability to predict prognosis in 65% and 52% of breast cancer test datasets, respectively (Additional file 1: Table S3). The signature with the most robust predictive ability was BRmet50, as it was able to predict prognosis successfully in 23 out of 31 breast cancer datasets (74%) [32].

Although phenotypes and study designs are heterogeneous among the 37 signatures, they share the same functional space in predicting breast cancer prognosis. These results support the notion that breast cancer clinical outcomes are associated with various mechanisms and tumor phenotypes.

Among the top 37 signatures (Additional file 1: Table S3), a few signatures are the result of direct design, in which a prognostic signature is derived from a direct comparison of two groups with opposite prognosis outcomes (e.g., signature PMID18231641Sig73 with phenotype relapse vs. non-relapse in Additional file 1: Table S3) [37]. However, the majority of the signatures are the result of indirect design, comparing phenotypes such as low or high proliferation (signature PMID18662380Sig355) [38] and TP53 status (signature PMID18271932Sig33) [39]. Regardless of study design, all 37 signatures were found to be associated with patient prognosis. Interestingly enough, the performances of the signatures derived via direct design are not necessarily better than those derived via indirect design.

### Random signature simulation identifies appropriate test datasets

The predictive ability of each of the 351 public cancer signatures varied across the 31 breast cancer test datasets. Assuming that the majority of the 351 public signatures are, in fact, associated with cancer prognosis, we can use them to identify those test datasets prone to producing false negative results, as these will be the datasets in which most of the public signatures will fail to appropriately stratify patients into their prognostic groups. Such instances in which signatures failed to stratify patients were recorded as “N/A” in the survival analyses. The percentages of failure rates (“N/A rates”) from the 351 signature log-rank tests are listed for each dataset in Additional file 1: Table S2.

The N/A rates in most test datasets were very low (<5%, Additional file 1: Table S2). However, four datasets including GSE10510, BR17663798, GSE2607GPL1390, and BR907 had unacceptably high N/A rates (>5%). These four datasets that performed poorly with most test signatures and resulted in predictions with high false-negative rates were subsequently removed before performing further survival analyses. Thus, we filtered the datasets with high N/A rates before performing other survival analyses.

To control false positive results from the log-rank tests using top 37 signatures and the 31 test datasets, we compared the predictive ability of each of the top 37 signatures to 1,000 random signatures of identical length (ranging from 12 to 1,019 genes).

For a given test dataset, we summarized the mean percentage of significant P values from the top 37 pubic cancer signatures and the mean percentage of significant P values from the 37,000 random signatures (Additional file 1: Table S3).

We then computed the differential index, which is defined as the difference between the mean percentage of significant P values from the top 37 signatures and the mean percentage of significant P values from the 37,000 random P values.

A high-performance breast cancer test dataset has a large differential index (≥9%), indicating a low percentage of significant P values from the 37,000 random signature simulations and a high percentage of significant P values among the top 37 signatures. Similarly, a poorly-performing dataset has a small differential index (<9%) because it has a high percentage of significant P values from the 37,000 random signature simulations.

Based on the differential index, nine datasets demonstrated poor performance given their small differential indices (<9%), which included the four datasets identified as having high N/A rates (Additional file 1: Table S2). Therefore, the 22 remaining breast cancer datasets were considered to be datasets with good test performance on the basis of a large differential index (≥9%).

### Meta-validation of top 37 prognostic signatures in breast cancer

To further narrow down the top signature candidates, we re-evaluated the top 37 cancer signatures’ performances in the 22 verified test datasets with adjusted P values from the 22,000 random signature simulations. Specifically, we adjusted the P values of the top 37 signature candidates in each test dataset using 1,000 randomly generated P values.

For a given test dataset and test signature, we expected that 5% or fewer of these 1,000 random signature P values would be smaller than the P value of the corresponding top 37 test signature.

Thus, an adjusted P value is determined dynamically by adjusting the P value from one of the top 37 signatures using the P values from 1,000 random signatures of equal length in the same test dataset. We counted the number of random signatures that had smaller P values than the P value of their corresponding top 37 signature and divided it by 1,000. We were thus able to obtain a new permutation-adjusted P value for each top signature.

We ranked the top 37 signatures by their adjusted median P values over the 22 validated breast cancer datasets (Table 1). BRmet50*,* PMID16505416Sig822, and PMID18271932Sig33 were the only signatures that could distinguish a good prognostic group from a poor prognostic group successfully in 82%, 68%, and 64% of the 22 test datasets, respectively, with adjusted median P values < 0.05. The 34 other prognostic signatures were unable to discriminate prognosis groups in the majority of test datasets when compared with random signatures of equal length (median P values > 0.05).

BRmet50 was deduced from data similarity-based meta-analysis and demonstrated robust prognostic prediction in multiple cancer types [32]. PMID16505416Sig822 was derived from estrogen-responsive genes identified by treating MCF7 cells with 17beta-estradiol and previously has been shown to add significant prognostic information independent of standard clinical predictors [40]. PMID18271932Sig33 was obtained by identifying gene transcripts differing between patients with or without TP53 mutations identified by DNA sequencing and was shown to be a significant prognostic factor for recurrence and survival in two external datasets [39].

For PMID18271932Sig33, the 22 validated datasets are fully-independent test datasets.

There is one out of the 22 datasets served as a training set [40] for PMID16505416Sig822. Among the 22 datasets, nine were used as training sets for BRmet50 [10,17,36,41-45], and remaining 13 are independent test datasets [3,6,40,46-53]. To avoid over-fitting of the nine training datasets, we had used a ‘leave-one-out’ cross-validation strategy to deduce nine BRmet50 control signatures for the corresponding nine training datasets [32]. In each leave-one-out trial, the included signatures remained clustered and shared the core set of the 50 genes. We had tested these control meta-signatures in corresponding training datasets and found that their prognostic performances were as good as BRmet50 [32].

### Multivariate comparison of the top three signatures identifies signatures with additional prognostic value in addition to standard clinicopathologic features

To further evaluate the performance of the top three signatures, we examined five datasets (BR1042, BR1095, BR1128, BR1141, and GSE7390) sharing a common set of clinicopathologic characteristics including tumor size, grade, lymph node status, and Nottingham Prognostic Index (NPI) [54,55]. We performed C-indices, log-rank tests, and univariate Cox proportional hazards model to compare the performance of the top three signatures. In addition, we performed multivariate Cox proportional hazards models to compare with other prognostic factors, namely, age, tumor size, grade, lymph node status, and NPI. The unadjusted and adjusted hazard ratios of these factors and the top three signatures were then determined (Table 2).

Univariate Cox proportional hazards analysis demonstrated that PMID16505416Sig822 could not successfully predict cancer prognoses in two out of the five datasets. However, BRmet50 and PMID18271932Sig33 were able to significantly differentiate tumor samples into two prognostic groups in all five validation datasets. The hazard ratios for BRmet50 and PMID18271932Sig33 were consistently greater than those of PMID16505416Sig822 as evidenced by the fact that optimal unadjusted hazard ratios (HR) (high risk vs. low risk) in BR1042 were 2.8 (95% CI: 1.4–5.5, P = 0.002) for BRmet50, 2.0 (95% CI: 1.1-3.8, P = 0.03) for PMID18271932Sig33, and 1.1 (95% CI: 0.4-1.7, P = 0.69) for PMID16505416Sig822, respectively (Table 2). These data suggest that the BRmet50 and PMID18271932Sig33 signatures more efficient at predicting relapse-free survival in BR1042, BR1141, and GSE7390 and disease-free survival in BR1095 and BR1128 than PMID16505416Sig822.

As another means of assessing performance, we calculated the c-index, which is a generalization of the area under the receiver operating characteristic (ROC) curve [56], for the cancer signatures in the 5 validation datasets (Table 2). The prognostic value (c-index) for BRmet50, PMID18271932Sig33, and PMID16505416Sig822 were compared. For any given test dataset, BRmet50 c-indices were similar to those of PMID18271932Sig33 but significantly higher than those of PMID16505416Sig822, suggesting that the prognostic information provided by the BRmet50 and PMID18271932Sig33 signatures were comparable but better than that of PMID16505416Sig822.

To determine if BRmet50, PMID18271932Sig33, and PMID16505416Sig822 added independent prognostic information to other standard clinicopathologic features, we performed multivariate Cox proportional hazards analysis. In this multivariate Cox proportional-hazards analysis (Table 2), significant associations (P < 0.05) were observed in all five test datasets between BRmet50 and patient relapse-free or disease-free survival time after adjustment for standard clinical covariates. Thus, BRmet50 contributed new and important prognostic information beyond that provided by established clinical predictors. Except for one analysis using BR1042, PMID18271932Sig33 also demonstrated significant association after adjustment for standard clinical covariates in the other four test datasets. On the other hand, PMID16505416Sig822 showed no significant associations in three test datasets after adjustment for standard clinical covariates.

Together the data suggested that BRmet50 and PMID18271932Sig33 had comparable predictive power while PMID16505416Sig822 showed poor performance in c-index and uni- and multi-variate Cox proportional hazards analyses.

### Prognostic signatures have predictive value in breast cancer subsets

To determine the performance of the top three signatures in different subsets of breast cancer, we evaluated their predictive power using a well-characterized dataset (BR1141) containing commonly used covariates.

The 269 patients from BR1141 were stratified according to tumor size, lymph node status, tamoxifen treatment, histologic grade, and ER status. A univariate Cox proportional hazards model was used to evaluate the association of individual signatures with the clinical outcome in each category (Table 3).

BRmet50 and PMID18271932Sig33 performed equally well in the ER+ and intermediate -grade subsets of BR1141. The association between the top two signatures and the risk of relapse was significant regardless of tumor size, lymph node status, and tamoxifen treatment (P < 0.05).

However, while the top two signatures were significantly associated with outcome in patients with ER+ tumors (hazard ratio = 2.5, P < 0.0001) this was not the case for those that were ER- (log-rank P = 0.495 and 0.329, HR = 1.4 and 1.7, 95% CI: 0.5–5.0). Both BRmet50 and PMID18271932Sig33 were incapable of stratifying tumors with high (grade 3) or low (grade 1) differentiation (P > 0.05).

PMID16505416Sig822 had no apparent predictive value for almost all subsets of the BR1141 dataset (P > 0.05), with the exception of the tamoxifen-treated patient subset, which is expected given its derivation from estrogen responsive cells [40].

### Identification of a prognostic signature for ER- breast cancer

Like most breast cancer signatures, BRmet50 and PMID18271932Sig33 are derived from datasets in which ER-positive (ER+) tumors predominant and perform well in the prediction of prognosis in ER+ tumors but poorly in ER- tumors (Table 4).

In order to identify signatures that could provide prognostic value in multiple ER- subsets, we created a subset of our 22 datasets consisting of nine ER- datasets. We tested 351 public cancer signatures with the nine ER- datasets using log-rank tests, and univariate and multivariate HR analyses. From our results, we identified one signature, PMID20813035Sig137 with good predictive value, which is enriched in adhesion/EMT genes and derived from ER- and claudin-low breast tumors [57]. PMID20813035Sig137 was able to predict prognosis successfully in 56% (five out of nine) of ER- datasets (Table 4) while BRmet50 and PMID18271932Sig33 were able to predict prognosis successfully in 11%. PMID20813035Sig137 produced statistically significant unadjusted HR values (P < 0.05) in five of the ER- datasets and significant adjusted HR values in four ER- datasets.

Among the 1,000 random signatures of lengths identical to PMID20813035Sig137 (137 genes), only PMID20813035Sig137 was able to predict prognosis in more than four out of nine ER- subsets. Therefore, the ability of PMID20813035Sig137 to predict prognosis in ER- subsets reached statistical significance.

### Prognostic signatures have predictive value for neo-adjuvant chemotherapy

Chemotherapy response is determined by primary tumor biology. Consequently, the initial development of a predictive signature for chemotherapy response does not necessary have to make use of treatment response and survival data, but can instead rely on indirect markers. For example, BRmet50 is actually derived from primary tumor samples with various phenotypes known to be associated with prognosis and treatment sensitivity (e.g., grade, size, proliferative rates, nodal status, and molecular markers). Since PMID16505416Sig822 was generated as an ER-responsive signature and only provided additional prognostic value in the tamoxifen-treated setting, we decided to evaluate the predictive value of the remaining two prognostic signatures to predict chemotherapy outcomes and drug sensitivity in breast cancer patients.

To prospectively assess the predictive value of the top two prognostic markers, we collected the five test datasets (GDS4057, GSE32646, GSE41998, GSE25055 and GSE25065) [6,58-60] containing neoadjuvant chemotherapy responses and gene-expression profiles. The chemotherapy regimens included anthracycline/taxane (GSE25055 and GSE25065), 5-fluorouracil/doxorubicin/cyclophosphamide (GDS4057), 5-fluorouracil/epirubicin/cyclophosphamide (GSE32646), and doxorubicin/ cyclophosphamide/ixabepilone/paclitaxel (GSE41998). We evaluated the predictive performance of the top two signature profiles by using the surrogates of both conventional short-term treatment response and long-term survival time. The short-term outcome measurements in all five test datasets (993 patients) include actual pCR/RD and RCB. The long-term survival measurement in two datasets (GSE25055 and GSE25065, 508 patients) is DRFS. The derived positive predictive value (PPV), the negative predictive value (NPV), sensitivity, and specificity are compared accordingly (Table 5).

Both BRmet50 and PMID18271932Sig33 can predict pCR/RD or RCB in five independent cohorts with those patients predicted to belong to the insensitive response group having high RD rates after neoadjuvant chemotherapy (mean PPV = 85%-88%) (Table 5). Those patients predicted to have a good response had significantly longer DRFS (i.e., no relapse within three years, NPV = 87%-88%) than those predicted to have early relapse and shorter DRFS (i.e., relapse within three years, PPV = 31%-32%). The NPV values of these two genomic predictors (BRmet50 and PMID18271932Sig33) were comparable to those of traditional predictive methods (pCR and RCB, 92% and 83%, respectively).

These results suggest that BRmet50 and PMID18271932Sig33 have similar predictive value in terms of predicting chemotherapeutic response. They can both accurately predict the clinical responses of breast cancer patients treated with commonly used chemotherapeutic drugs, especially for those patients who will go on to fail chemotherapy as assessed by residual disease (RD or RCB-II/III) upon surgery or 3-year DRFS. This predictive power is of significant clinical importance because it has the ability to identify those patients most likely to fail chemotherapy and would likely experience chemotherapy toxicity without the benefit of halting or slowing disease progression.

To determine if the predictive power of these signatures translates into prognostic value, we compared DRFS for the top two signatures using Kaplan–Meier survival analysis (Figure 2). The distant relapse-free survival time for the groups predicted to be treatment-sensitive was significantly longer than that of the groups predicted to be treatment-insensitive (P < 0.01) and followed a similar curve as the actual pathologic response assessed by pCR. For example, the 5-year disease-free survival rates were 82%-85% for the BRmet50 and PMID18271932Sig33-predicted responders while the relapse-free rates were 61%-65% for the BRmet50 and PMID18271932Sig33-predicted insensitive groups*.*

These results demonstrate that these signatures have predictive power for neoadjuvant chemotherapy in addition to their long-term prognostic value.

### The predictive value of the prognostic signatures in HER2-negative patients treated with chemotherapy

Since ERBB2 (HER2 or HER2/neu) positive patients receive targeted therapy there exists a need to determine if newly diagnosed HER2-negative patients would benefit from the use of neoadjuvant taxane-anthracycline chemotherapy, as assessed by short-term treatment response (pCR and RCB0/I) and, more importantly, long-term (DRFS) prognosis [6].

Given our finding that the top two prognostic signatures can retrospectively predict some common chemotherapeutic outcomes in breast cancer patients (Table 5), we designed an algorithm using the BRmet50 and PMID18271932Sig33 predictors for prospective prediction of taxane-anthracycline sensitivity in individual patients with HER2- breast cancer.

In order to build a model for taxane/antracycline chemotherapy we extracted the drug sensitivities for 13 HER2-negative breast cancer cell lines from the publically available Genomics of Drug Sensitivity (GDS) dataset [33]. Using these cell lines we created a taxane-anthracycline-sensitive reference profile for sensitive/resistant cell lines (log(IC50) < −1) and compiled gene expression centroids for each. Using these centroids, we generated treatment sensitive and insensitive signatures for BRmet50 and PMID18271932Sig33 [3]. These signatures were then correlated to gene expression from HER2-negative patients in GSE25055 and GSE25065 who were subsequently treated with chemotherapy containing sequential taxane and anthracycline-based regimens.

To evaluate the ability of the two signatures to predict drug sensitivity, the groups predicted to be either drug-sensitive or drug-insensitive were compared to their actual short-term drug responses (pCR/RD) and long-term DRFS outcomes. We then computed performance values (PPV, NPV, sensitivity, and specificity) and performed Kaplan-Meier analyses.

We have presented two prediction models for drug responses in breast cancer patients: (1) an unsupervised clustering-based retrospective prediction and (2) a drug-sensitive centroid-based prospective prediction (Table 6). We found that BRmet50 was capable of predicting drug sensitivity in both models.

When drug sensitivity prediction results were assessed by actual treatment response (pCR/RCB) in HER2- breast cancer patients, we found that the prospective individual predictions using the centroid model showed comparable results to those obtained using the unsupervised clustering-based prediction. Both had high PPV (85%-93% vs. 74%-89%, respectively) and specificity (71%-83% vs. 67%-83%, respectively) (Table 6). The BRmet50 predictions had higher PPVs and NPVs than did the PMID18271932Sig33 predictions. For example, the average PPVs of BRmet50 predictions were 82%-89% while the average PPVs of PMID18271932Sig33 were 74%-77%.

The taxane-anthracycline sensitivity predictions based on the BRmet50 and PMID18271932Sig33 centroids were evaluated by long-term drug response (i.e., distant relapse-free survival) using Kaplan-Meier analyses (Figure 3). The results demonstrated that there was a significant difference in distant relapse-free survival between the patients who were predicted to be drug-sensitive and those who were predicted to be drug-insensitive by the BRmet50 centroid (P < 0.05, Figure 3) while PMID18271932Sig33 centroids failed to achieve statistically significance (P > 0.05). Thus, the BRmet50 centroid model can predict significantly improved DRFS for patients with taxane-anthracycline sensitivity. The ability of BRmet50 to predict drug sensitivity is better than that of PMID18271932Sig33, which is consistent with the PPV and NPV data in Table 6.

The data suggests that a combination of a prognostic signature and chemosentivity data from pre-clinical breast cancer cell lines can prospectively predict chemotherapy sensitivity in individual patients with HER2- breast cancer.

### Gene signature annotation analyses of the top two prognostic signatures pathways associated with TP53 mutations

Since BRmet50 and PMID18271932Sig33 have similar prognostic patterns, we anticipated that there may be a large number of overlapping genes and gene functions between the two signatures that shed light on a fundamental mechanism of cancer prognosis prediction.

We previously examined the 50 genes in BRmet50 with regard to their functions and relevance to cancer (Additional file 1: Table S4) [32]. Some of the genes in the signature are known to be involved in tumor progression [61-67]. Of the 50 genes in BRmet50, 39 were up-regulated and 11 were down-regulated in aggressive tumors. More than half of the BRmet50 gene-expression directions and functions have been confirmed in publicly accessible data (29/50), but the other 21 have not yet been confirmed and represent potential functional genes involved in cancer progression and metastasis.

Among the 32 genes in the PMID18271932Sig33 signature, there are 20-upregulated genes and four down-regulated genes in samples with TP53 mutations (Additional file 1: Table S5). The remaining eight gene-expression directions are unknown. Of the 20 upregulated genes, 14 genes have molecular functions related to the cell cycle and/or cell division.

Only seven genes in BRmet50 overlap with PMID18271932Sig33, suggesting that BRmet50 and PMID18271932Sig33 are two distinct signatures. However, most genes from both signatures are involved in cancer tumorigenesis and tumor progression. Using Ingenuity pathway analysis, we found that the BRmet50-identified genes are involved in 45 different pathways, and that the PMID18271932Sig33-identified genes are involved in 24 distinct pathways. Between the two lists, we identified 10 common canonical pathways (Table 7).

**Table 7:** **Major pathways of BRmet50 and PMID18271932Sig33**

Pathways	BRmet50	PMID18271932Sig33
Cell cycle: mitotic roles of polo-like kinase	KIF23,PRC1,CCNB2	KIF23,PTTG1,PRC1,CCNB2,PKMYT1,PLK1
Cell cycle: G2/M checkpoint regulation	CCNB2	CCNB2,PKMYT1,PLK1
Cell cycle: control of chromosomal replication	CDC45,CDT1	CDC45
Cell cycle: regulation	CCNB2	CCNB2
Cell cycle: checkpoint control	RFC4	PLK1
DNA damage: 14-3-3σ signaling	CCNB2	CCNB2
DNA damage repair: ATM signaling	RAD51,CCNB2	CCNB2
DNA damage response: BRCA1 pathway	RAD51,RFC4	PLK1
DNA damage response: salvage pathways of pyrimidine ribonucleotides	NEK2	PLK1
Protein ubiquitination	UBE2S,UBE2C	UBE2C

Both signatures are heavily enriched for genes involved in cycle checkpoint regulation and DNA damage response, (Table 7). Since cell cycle control is directly or indirectly disrupted by TP53 mutations in tumor cells, the overlapping functions in BRmet50 and PMID18271932Sig33 may represent gene-expression alterations resulting from the loss of TP53 [39].

**Figure 4:**
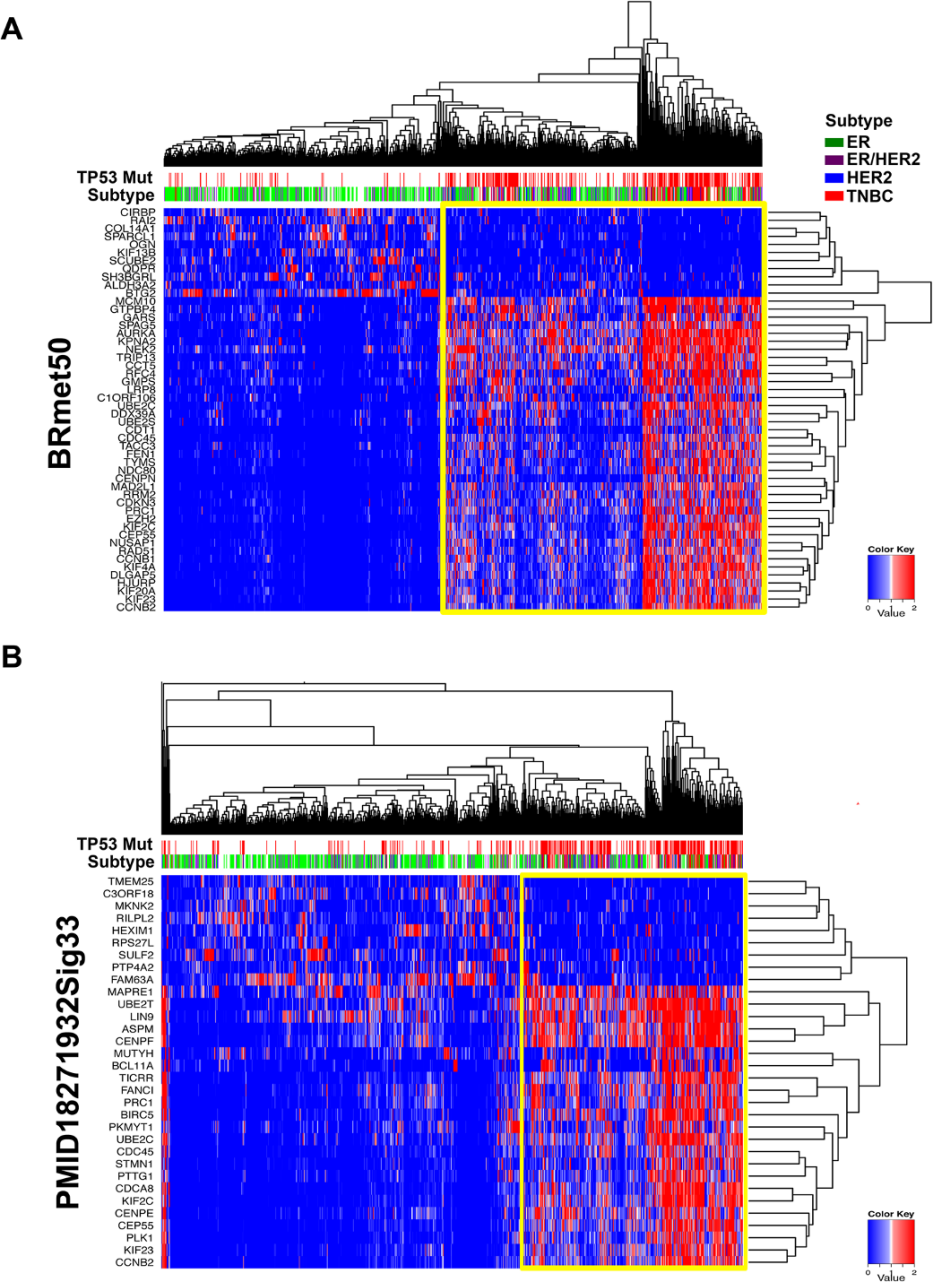
**BRmet50 and PMID18271932Sig33 enrich for breast cancer tumors with mutated TP53.** Heat maps display unsupervised hierarchical clustering for genes in **(A)** BRmet50 and **(B)** PMID18271932Sig33 in the breast cancer TCGA cohort. TP53 mutation (red) and breast cancer subtypes are indicated in the color bars above the heat map.

To determine if both signatures identify tumors with TP53 mutations, we examined the TCGA breast cohort and extracted the gene expression data (RNA-seq) for each gene comprising the signature as well as TP53 mutation status. Of the 958 tumors with both gene expression data and TP53 mutation status, 30.3% (290) of the tumors exhibited TP53 mutations (Additional file 1: Table S6). Unsupervised hierarchical clustering performed on the gene expression for each signature showed a substantial enrichment in TP53 mutations for tumors with elevated gene-expression of both PMID18271932Sig33 (56.3% vs. 13.1%, P < 0.0001) and BRmet50 (48.7% vs. 8.5%, p < 0.0001) (Figure 4). While enriched for TNBC tumors, known to carry a high frequency of TP53 mutations, the clustering appears to be subtype-independent with numerous ER+ and HER+ tumors clustering with TNBC tumors. The tumors enriched by both signatures were highly correlated (82.2%), suggesting that both signatures may be a functional gene expression readout loss of TP53 function states.

## Discussion

Hundreds of transcriptional gene signature studies published to date have not progressed beyond the discovery phase because the validation phase of gene-expression signatures is very time-consuming and costly. Additionally, many prognostic signatures often fail to reproduce in independent datasets. Therefore, we developed a high-throughput *in silico* validation method capable of identifying signatures with robust prognostic value through systematical evaluation of the performance of 351 public cancer signatures across 31 breast cancer validation datasets.

Because almost all public cancer signatures have only been validated using a few test datasets, a practical question is how to select additional valid datasets for signature validation. Therefore, we developed a differential index system using 37,000 random signatures. We identified nine datasets that were considered to be poorly-performing datasets and 22 breast cancer datasets that were considered to exhibit good performance. For the first time, our study proposes a criterion for validation of public datasets by providing 22 validation datasets from 31 available breast cancer datasets for survival analysis. The results suggest that the differential index should be implemented as an additional inclusion/exclusion criterion for the selection of validation datasets.

While it is true that many other studies have previously shown that signatures related to cell cycle or proliferation [68,69] or p53 pathway [10,70] can predict outcome in one or a few tumor datasets, this is not the case when evaluated in a large sample size (Additional file 1: Table S1). Based on the rank order of 351 prognostic signatures’ performance in 10,881 log-rank tests, we identified two prognostic signatures (BRmet50 and PMID18271932Sig33) in breast cancer and one signature (PMID20813035Sig137) specific for prognosis prediction in patients with ER-negative tumors. However, many other signatures are unable to predict prognosis in more than 50% of breast cancer datasets (Table 1).

We also computed Q values based on multiple hypothesis testing of 10,881 P values from the log-rank tests [71]. The percentages of significant Q values (false discovery rate < 8%) were then ranked accordingly, and a similar ranking pattern as the significant P value rates has been observed (data not shown). Most of top37 signatures (33/37) had significant Q values in more than 50% of test datasets except the three signatures (PMID12714683Sig29, PMID19014521Sig72, PMID21501481Sig224) having the significant Q values in 48% test datasets.

A signature can be designed directly from clinical outcome comparisons or indirectly by making comparisons between various molecular mechanisms and disease phenotypes as opposed to survival data. After the first round of validations, only two (PMID15721472Sig76 and PMID17076897Sig52) found in the top 37 signature list were directly designed signatures derived from DRFS comparisons. The top two prognostic signatures were indirectly designed. BRmet50 was derived from our previously implemented meta-analysis of breast cancer gene-expression profiles [7], and PMID18271932Sig33 is derived from TP53 mutation status, which is the most common and fundamental genomic alteration in cancer [39,72]. Not only can the two signatures (BRmet50 and PMID18271932Sig33) predict the clinical responses of breast cancer patients to commonly used chemotherapies, but also both signatures can retrospectively predict cancer treatment response (pCR/RD) and survival (DRFS) to neoadjuvant chemotherapy. Furthermore, BRmet50 can prospectively predict taxane-anthracycline sensitivity in patients with HER2-negative (HER2-) breast cancer (Table 6). Since cancer prognosis correlates with many aspects of cancer biology and clinical phenotypes, our results (Additional file 1: Table S3 and Table 1) suggest that indirectly-designed signatures are robust for the prediction of cancer prognosis. However, not all indirectly-designed signatures can be used for prediction of short-term treatment response. For instance, the prognosis signature PMID11823860Sig70 (MammaPrint) can predict prognosis in patients with breast cancer but was unable to reliably predict pCR in breast cancer patients predicted to have a good long-term, prognosis [29].

We realized that many well-known predictive signatures in Additional file 1: Tables S1 and S3 were not included in the top signatures (Table 1). For example, PMID11823860Sig70 (MammaPrint) was designed specifically to predict distant metastasis in early-stage breast cancer patients with lymph node-negative status [36,41]. The 21-gene Recurrence Score assay from PMID18360352Sig21 (Oncotype DX Breast Cancer Assay) is prognostic for women treated with tamoxifen with lymph node-negative and ER+ breast cancer [2,30]. One explanation is that we did not compare those well-known signatures within their own individual predictive conditions as described in the original studies.

The source datasets we analyzed in this study are all from microarray platform. The common limitation with microarray is high background noise interference introduced at different experimental and analysis stages. This problem often affects the data quality for meta-analysis. We expect that RNA-seq technology based gene-expression profiles will improve data quality and has a revolutionary impact on the meta-analysis of gene-expression research.

One obvious limitation of the BRmet50 and PMID18271932Sig33 is that they cannot predict prognosis in patients with ER- tumors. Almost all public cancer signatures including BRmet50 and PMID18271932Sig33 are derived from whole tumor samples. Intertumor and intratumor heterogeneity of whole tumors is evident in their histology, gene expression, genotype, and metastatic and proliferative potential [73,74]. The major subtype of whole primary tumors is ER-positive (ER+) (~60%). Thus, our top two signatures, various multi-gene assays such as the 70-gene MammaPrint signature (designated here as BRsig70) [36], the 76-gene signature (BRsig76) [41], and many others [1,75-78], which have been developed for clinical prognosis prediction in classifying ER+ patients into low- or high-risk groups for recurrence, are considerably less informative for ER- patients [79-86].

In order to identify prognostic signatures for patients with ER- breast cancer, we examined public signatures in the setting of ER- breast cancer and identified PMID20813035Sig137 as a good predictor of prognosis in patients with ER- tumors. The PMID20813035Sig137 signature is derived from breast tumors of the claudin-low tumor subtype. Claudin-low tumors are characterized by low to absent expression of luminal differentiation markers, and high enrichment for epithelial-to-mesenchymal transition markers, immune response genes, and cancer stem cell-like features. Clinically, the majority of claudin-low tumors carry a poor prognosis and are ER-, progesterone receptor (PR)-negative, and HER2- (triple negative) invasive ductal carcinomas [57]. Our data suggest that the predictor of prognosis in ER- tumors should be identified independently from ER- subset samples rather than from whole tumor samples, and a unique predictive model is required for those patients with ER- breast cancer.

PMID18271932Sig33 is comparable to BRmet50 in terms of predicting prognosis (Tables 1, 2, and 3), treatment response, and DRFS in breast cancer patients following neoadjuvant chemotherapy. PMID18271932Sig33 is derived from a set of genes that were differentially expressed between mutant TP53 and wild type TP53 tumors [39]. When BRmet50 and PMID18271932Sig33 genes are analyzed by an overlapping analysis, there are a limited number of common genes. Seven genes are common between BRmet50 (14%) and PMID18271932Sig33 (22%). Despite this small amount of overlap in gene composition, like many other prognostic signatures (Additional file 1: Table S1), the major functions of both signatures are essentially equivalent in prognostic performance in breast cancer [87]. The gene functions of the two signatures are common and highly correlated with cell cycle controls and cell proliferation (Additional file 1: Tables S4 and S5) [7,38,81], which stands in contrast to the gene composition and functions of the ER- predictor (PMID20813035Sig137). The 10 pathways shared by the top two signatures are essential for cancer prognosis and drug sensitivity. Most genes in the 10 common pathways are related to cell cycle and DNA damage response (Table 7, Additional file 1: Tables S4 and S5). Taxane and anthracycline are known for their ability to bind DNA in several different ways and inhibit cancer cell division and duplication of DNA for mitosis and DNA replication [88].

TP53 mutations are the most common genetic alterations in many types of cancer. specifically ranging from 20% to 50% in breast cancer [39]. In high-grade ovarian adenocarcinomas, TP53 is mutated in almost all tumors (96%) [72]. However, TP53 is an unpredictable tool for individual risk evaluation, metastasis, and overall survival. It is not easy to correctly evaluate TP53 status and its correlated clinical outcomes by standard DNA sequencing analysis [89], and there is no significant association with recurrence-free survival (RFS) between the two different TP53 statuses [39].

Our results suggest that the TP53 mutation-driven gene-expression signature (PMID18271932Sig33) is a good biomarker for prognosis prediction in breast cancer. PMID18271932Sig33 represents the functional consequences of TP53 mutations that are relevant to the TP53 characterization of molecular pathways in tumorigenesis, drug sensitivity, and the prognosis of several cancers.

## Conclusions

Our study provides a high-throughput validation method for assessing the prognostic value of all available public gene-expression signatures in breast cancer patients and 22 breast cancer datasets that are useful for survival analyses. Using this method, we identified two prognostic and TP53 mutation-driven signatures (BRmet50 and PMID18271932Sig33) in breast cancer and one signature (PMID20813035Sig137) specific for prognosis prediction in patients with ER-negative tumors. Moreover, our results suggest that indirectly designed prognostic signatures can retrospectively predict patient response to chemotherapy and prospectively predict taxane-anthracycline sensitivity for individual patients with HER2- breast cancer.

## Additional file

Additional file 1: **Table S1.** Overview of meta-analysis of signatures in cancer. **Table S2.** List of validation datasets in breast cancer. **Table S3.** Identification of prognostic signature candidates in breast cancer. **Table S4.** Annotation of genes in BRmet50. **Table S5.** Annotation of genes in PMID18271932Sig33. **Table S6.** TP53, ER, PR, and HER2 statuses in TCGA tumor samples.

## Abbreviations

BRmet50: 50-gene signature
BRsig70: The 70-gene signature in breast cancer or MammaPrint
BRsig76: The 76-gene signature in breast cancer
Oncotype DX: The 21-gene signature
CI: Confidence interval
ER: Estrogen receptor)
GEO: Gene Gxpression Omnibus
HR: Hazard ratio
NCBI: The National Center for Biotechnology Information
NPI: Nottingham Prognostic Index
ROC: Receiver operating characteristic
pCR: Pathologic complete response
RD: Residual disease
RCB: Residual cancer burden
NCBI: National Center for Biotechnology Information
TCGA: The Cancer Genome Atlas
DSS: Disease-specific survival
DFS: Disease-free survival
DMFS: Distant metastasis-free survival
OS: Overall survival
RFS: Relapse-free survival
DRFS: Distant relapse-free survival
HER2: Human epidermal growth factor receptor 2
GDS: Sanger Genomics of Drug Sensitivity (GDS)
IC50: Drug concentration leading to 50% growth inhibition of cancer cells compared to controls
PPV: Positive predictive value
NPV: Negative predictive value
