# Supplementary material for: Evaluation of public cancer datasets and signatures identifies TP53 mutant signatures with robust prognostic and predictive value
Source: BMC Cancer. 2015 Mar 26;15:179. doi: 10.1186/s12885-015-1102-7 (PMC4404582; doi:10.1186/s12885-015-1102-7)
Supplement: Additional file 1: — Table S1. Overview of meta-analysis of signatures in cancer. Table S2. List of validation datasets in breast cancer. Table S3. Identification of prognostic signature candidates in breast cancer. Table S4. Annotation of genes in BRmet50. Table S5. Annotation of genes in PMID18271932Sig33. Table S6. TP53, ER, PR, and HER2 statuses in TCGA tumor samples. [file 12885_2015_1102_MOESM1_ESM.pdf]

Table S1. Overview of meta-analysis of signatures in cancer

Signature ID	Description	Disease
PMID10741968Sig43	Fibroblasts from Hutchinson-Gilford Progeria	Aging
PMID14769913Sig3413	Alzheimer’s Disease(AD)-Related Genes”	Alzheimer’s
PMID14769913Sig609	609 “Incipient ADGs” or IADGs	Alzheimer’s
PMID11559565Sig102	Glioblastoma Multiforme (GBM)	Brain cancer
PMID10582678Sig16	Metastatic breast cancer	Breast Cancer
PMID10963602Sig412	Intrinsic Gene Subset	Breast Cancer
PMID11165872Sig16	MCF-7 Cell Line Overexpressing MnSOD	Breast Cancer
PMID11207349Sig51	BRCA1, BRCA2, and the Sporadic Tumors	Breast Cancer
PMID11553815Sig33	SAM264 Clone Set (Luminal/ER+ Cluster)	Breast Cancer
PMID11553815Sig40	SAM264 Clone Set (Basal Epithelial Cluster)	Breast Cancer
PMID11553815Sig66	SAM264 Clone Set (Proliferation Cluster)	Breast Cancer
PMID11562467Sig100	ER+ Tumor Samples	Breast Cancer
PMID11823860Sig70	MammaPrint for breast cancer relapse prediction	Breast Cancer
PMID11988840Sig170	Cancer Progression Signature	Breast Cancer
PMID12096084Sig110	110 BRCA-Discriminating Genes	Breast Cancer
PMID12414654Sig70	Treatment with Hydrogen Peroxide, Menadione, or t-Butyl Hydroperoxide in MCF7 Human Breast Cancer Cells	Breast Cancer
PMID12495447Sig57	Rho^0 (Devoid of mtDNA) Breast Cancer Cell Line	Breast Cancer
PMID12538167Sig23	23-Gene Set	Breast Cancer
PMID12610208Sig60	Classification of familial non-BRCA1/BRCA2	Breast Cancer
PMID12714683Sig200	Top 200 Genes Correlating with Tumor Grade	Breast Cancer
PMID12714683Sig29	Cluster of 29 Genes Showing Consistent Up-Regulation in IDC	Breast Cancer
PMID12714683Sig85	85 Genes with Increased Expression in IDC	Breast Cancer
PMID12747878Sig164	Genes Associated with Metagene Predictors of Breast Cancer Recurrence	Breast Cancer
PMID12747878Sig176	Genes Associated with Metagene Predictors of Lymph-Node Metastasis	Breast Cancer
PMID12829800Sig500	Intrinsic Gene List	Breast Cancer
PMID12842083Sig102	In Vivo-Selected Bone Metastasis Signature	Breast Cancer
PMID12842083Sig11	11 Genes that Differed by >4-Fold in Expression Level between Weak and Strong Metastatic Groups	Breast Cancer
PMID12917485Sig137	High-Grade Breast Tumors	Breast Cancer
PMID12917485Sig485	Breast Cancer Relapse	Breast Cancer
PMID12917485Sig606	ER+ Breast Tumors	Breast Cancer
PMID12917485Sig706	One of these Six Subgroups of Breast Cancer Tumors: Her-2/neu, Basal-Like 1, Basal-Like 2, Luminal-Like 1, Luminal-Like 2, or Luminal-Like 3	Breast Cancer
PMID14737112Sig32	HMGA1a-Induced Expression Profile	Breast Cancer
PMID14871811BCSig39	Breast Carcinomas	Breast Cancer
PMID15073102Sig13	13-Gene Signature	Breast Cancer
PMID15073102Sig14	14-Gene Signature	Breast Cancer
PMID15073102Sig3	3-Gene Signature	Breast Cancer
PMID15073102Sig4	4-Gene Signature	Breast Cancer
PMID15073102Sig5	5-Gene Signature	Breast Cancer

PMID15073102Sig6	6-Gene Signature	Breast Cancer
PMID15136595Sig74	Multigene Predictor of Pathologic Complete Response (pCR) to Sequential Weekly Paclitaxel and Fluorouracil + Doxorubicin + Cyclophosphamide (T/FAC) Neoadjuvant Chemotherapy Regimen for Breast Cancer	Breast Cancer
PMID15171711Sig96	Estrogen-Stimulated ZR-75·1 Cells	Breast Cancer
PMID15193263Sig2	HOXB13:IL17BR Expression Ratio	Breast Cancer
PMID15235906Sig21	5-year Postoperative Poor Prognosis of Primary Breast Cancers	Breast Cancer
PMID15235906Sig23	5-year Postoperative Good Prognosis of Primary Breast Cancers	Breast Cancer
PMID15254972Sig61	ERα-Positive Cell Lines and Tumor Biopsies	Breast Cancer
PMID15260889Sig681	Immortal Cell Lines	Breast Cancer
PMID15318932Sig50	Ductal Carcinoma in situ (DCIS)	Breast Cancer
PMID15588851Sig10	Hormones, Growth Factors and Receptors Showing 8-Fold or Greater Change in mRNA Expression between the Tumor and Normal Breast Tissue	Breast Cancer
PMID15588851Sig4	Genes Located Near the Breakpoint Regions (8p23 and 16q21)	Breast Cancer
PMID15598354Sig90	90-Gene Meta-Signature	Breast Cancer
PMID15637295Sig111	MCF-7 Cells Transfected Overnight with Empty pcDNA3 Vector and Postincubated with Estrogen (E2) (1 μM, 24 h)	Breast Cancer
PMID15637295Sig96	CON (Untransfected) MCF-7 Cells Postincubated with Estrogen (CON+E2) (1 μM, 24 h)	Breast Cancer
PMID15679045Sig13	Breast Cancer Cells Exposed to Dexamethasone (Dex)	Breast Cancer
PMID15681518Sig81	Tamoxifen-Responsive Estrogen Receptor-Positive Primary Breast Carcinomas	Breast Cancer
PMID15721472Sig76	76-Gene Prognostic Signature (for Development of Metastasis)	Breast Cancer
PMID15735024Sig3373	Genes whose Expression is Correlated with that of Neighboring Genes in Infiltrating Ductal Breast Carcinoma	Breast Cancer
PMID15762987Sig183	Estrogen Receptor Alpha Status Signature	Breast Cancer
PMID15869330Sig786	Stromal Genes Signature	Breast Cancer
PMID15897907Sig3198	Luminal/Apocrine/Basal Gene Set	Breast Cancer
PMID15899795Sig50	Breast cancer cell proliferation signature	Breast Cancer
PMID16091131Sig31	Preoperative Systemic Chemotherapy (PST) with Epirubicin/Cyclophosphamide (EC) Favorable Outcome Signature	Breast Cancer
PMID16115903Sig298	breast cancer all subtypes	Breast Cancer
PMID16141321Sig32	32-gene p53 signature	Breast Cancer
PMID16280042Sig64	64-Gene Set	Breast Cancer
PMID16317158Sig23	Epigallocatechin-3 Gallate (EGCG) Signature	Breast Cancer
PMID16322341Sig69	Endometria Obtained from Women Using Tamoxifen	Breast Cancer
PMID16457687Sig76	TGF-β in HER-2 Overexpressing Mesenchymal MDA-MB-231	Breast Cancer
PMID16465392Sig17	AS-raf-ODN Treated (Raf-1 Protein Kinase Inhibited) Cells	Breast Cancer
PMID16478745Sig97	97-Gene List	Breast Cancer
PMID16491069Sig45	Breast Cancer Proliferation Cluster	Breast Cancer

PMID16495925Sig43	TGFβ Pathway, EMT Control and Cytoskeletal (Re)organization Signature	Breast Cancer
PMID16505416Sig822	Estrogen-SAM List	Breast Cancer
PMID16536878Sig232	ANOVA-Analysis Output	Breast Cancer
PMID16536878Sig27	PAM-Analysis Output	Breast Cancer
PMID16626501Sig53	53 Classifier Genes	Breast Cancer
PMID16636340Sig87	Genes Involved in Bone Metastasis of Breast Cancer	Breast Cancer
PMID16643655Sig306	Single Sample Predictor	Breast Cancer
PMID16651414Sig527	List of 527 Probe Sets Significantly Differentially Expressed between Medullary and Ductal Breast Cancers	Breast Cancer
PMID16752223Sig4	ABC Transporters Selected as Best Classifiers at a Significance Threshold of 0.003	Breast Cancer
PMID16849555Sig22	Identities of the 22 Genes Coordinately Repressed Late in 3D Time Course	Breast Cancer
PMID16849555Sig60	List of All 60 3D Differential Genes on Affymetrix HG-U133A and Rosetta Chips	Breast Cancer
PMID16886892Sig57	Fibrocystic Breast Cell Line (MCF-10a) Exposed to 10 μM Lycopene for 48 h	Breast Cancer
PMID16896004Sig30	30-Probe Set Predictor (DLDA-30)	Breast Cancer
PMID16936776Sig200	Top 200 Cox-Ranked Genes	Breast Cancer
PMID16965636ERBB2Sig10	BCMP11/ABCC11 Group 2	Breast Cancer
PMID16965636ESR1Sig10	BCMP11/ABCC11 Group 1	Breast Cancer
PMID16965636Sig10	BCMP11/ABCC11 Group 3	Breast Cancer
PMID16979889Sig60	Well-Established Breast Cancer Cell Line with High Metastatic Potential (MDA-MB-435HM)	Breast Cancer
PMID17016441Sig5	Cytokeratin induced genes in Basal-Like Cancers	Breast cancer
PMID17023159Sig20	Remained Free from Distant Recurrence for 40 Months or Longer	Breast Cancer
PMID17069663Sig35	Ductal Carcinoma in situ	Breast Cancer
PMID17069663Sig43	Well-Differentiated DCIS	Breast Cancer
PMID17069663Sig78	Well/Well-Intermediately-Differentiated DCIS	Breast Cancer
PMID17069663Sig80	Poorly-Differentiated DCIS Samples	Breast Cancer
PMID17076897Sig52	Consensus Prognostic Gene Expression Classifier for ER-Positive (+) Breast Cancer	Breast Cancer
PMID17079448Sig264	PAM 264-Gene Classifier	Breast Cancer
PMID17123152Sig79	Lymph Node Metastases	Breast Cancer
PMID17150101Sig52	p53 Loss Signature	Breast Cancer
PMID17157792Sig66	Genes in Recurrent Amplicons Associated with Reduced Survival Duration in Breast Cancer	Breast Cancer
PMID17160137Sig59	59 RB (Retinoblastoma Tumor Suppressor) Target Genes	Breast Cancer
PMID17224074Sig16	Tumor Samples with Somatic Alterations	Breast Cancer
PMID17245340Sig15	List of Overexpressed Candidate Genes in Breast Cancer Compared to Normal Tissues, Identified by cDNA (EST) and the SAGE DGED from the CGAP Server	Breast Cancer
PMID17349583Sig1208	SAGE Tags Statistically Significantly Differentially Expressed Between Metastatic CD44+ and CD24+ Cells	Breast Cancer
PMID17404078Sig50	ERβ Gene Expression Signature	Breast Cancer
PMID17404081Sig28	28-Gene Signature for Breast Cancer Recurrent and Metastatic Potential	Breast Cancer

PMID17428335Sig182	PAM Gene List	Breast Cancer
PMID17428335Sig22	Overlap Genes	Breast Cancer
PMID17428335Sig52	Correlation Genes	Breast Cancer
PMID17428335Sig63	SAM Gene List	Breast Cancer
PMID17555561Sig2659	Pre-Treatment Biopsies of Primary Estrogen Receptor Positive Breast Cancer Patients Assigned to Treatment with Anastrozole or Letrozole for 2 Weeks before Surgery	Breast Cancer
PMID17640171Sig7	Genistein-Treated HCC1395 Breast Cancer Cells	Breast Cancer
PMID17663798Sig139	Cluster #3 (EGFR-Associated Signature Highly Enriched for Luminal A and B Tumors and Highly Expressed in Most of the HER2+/ER- and Basal-Like Tumors that were also High for Cluster #2)	Breast Cancer
PMID17663798Sig27	Cluster #2 (EGFR-Associated Signature Enriched in 58% of All Basal-Like Tumors, 48% of All HER2+/ER- Tumors and 3 Luminal B Tumors)	Breast Cancer
PMID17663798Sig35	Cluster #1 (EGFR-Associated Signature Enriched in a Mix of Breast Tumors that Contained All Five Breast Cancer Subtypes: Luminal A, Luminal B, Basal-Like, HER2+/ER-, and Normal-Like Samples)	Breast Cancer
PMID17664038Sig18	Treatment of MCF-7 Cells with 17β-estradiol (E_2) and the Extract of *Glycyrrhiza glabra* root	Breast Cancer
PMID17785550Sig67	DTC (Disseminated Tumor Cells) Signature	Breast Cancer
PMID17804718Sig61	SV40 T/t-antigen Oncogene Signature	Breast Cancer
PMID17883867Sig60	60-Gene Profile	Breast Cancer
PMID17885619Sig143	breast cancer with Aromatase Inhibitor Letrozole	Breast Cancer
PMID17899370Sig45	Stromal Fibroblasts Deriving from Malignant Tissue of Women with Invasive Breast Cancer	Breast Cancer
PMID17899371Sig374	374 Gene Set	Breast Cancer
PMID17952122Sig4	Four Epithelial Genes	Breast Cancer
PMID17999412Sig24	Inflammatory Breast Cancer Tumor Stroma	Breast Cancer
PMID18024211Sig35	Cyclophosphamide 35 Probesets	Breast Cancer
PMID18024211Sig46	Fluorouracil 46 Probesets	Breast Cancer
PMID18024211Sig47	Docetaxel 47 Probesets	Breast Cancer
PMID18024211Sig74	Doxorubicin 74 Probesets	Breast Cancer
PMID18044827Sig278	One of these Four Main Groups of Tumors: ECM1, ECM2, ECM3, or ECM4	Breast Cancer
PMID18199711Sig229	Breast Epithelium of Parous Control Women	Breast Cancer
PMID18231641Sig73	73-Gene Outcome Signature	Breast Cancer
PMID18271932Sig33	Predictor Gene Set for TP53 Status	Breast Cancer
PMID18278552Sig24	Integrated 24-Gene Signature	Breast Cancer
PMID18304324Sig112	112-Gene Breast Cancer Prognostic Signature	Breast Cancer
PMID18338247Sig14	Genes Uniquely Regulated by Tamoxifen	Breast Cancer
PMID18338247Sig183	Estrogen-Regulated Genes that Changed with Aromatase Inhibitor and Aromatase Inhibitor + Tamoxifen Treatment	Breast Cancer
PMID18338247Sig23	Tamoxifen "Estrogen-Like" Agonist Genes whose Expression Changes with Treatment	Breast Cancer
PMID18338247Sig25	Endocrine Therapy Response	Breast Cancer
PMID18338247Sig298	Genes Regulated by Tamoxifen in the Same Manner as Estrogen	Breast Cancer
PMID18338247Sig736	Genes Expressed Differentially in Nonresponders	Breast Cancer

	Compared to Responders	
PMID18347175Sig36	36-Gene PAM Signature	Breast Cancer
PMID18360352Sig21	Oncotype DX Breast Cancer Assay	Breast Cancer
PMID18366635Sig30	Pathologic Complete Response (PCR) to Weekly Paclitaxel Administration Followed by Chemotherapy with 5-Fluorouracil, Doxorubicin, and Cyclophosphamide (T/FAC)	Breast Cancer
PMID18381933Sig1001	ERBB2-Amplified Ductal Carcinoma in situ	Breast Cancer
PMID18387200Sig1488	Human Estrogen-Responsive Breast Cancer Cells (ZR-75.1) Cultured in Steroid-Free Medium for 4 Days	Breast Cancer
PMID18414401Sig66	Desmoid-Type Fibromatosis Core Gene Set	Breast Cancer
PMID18425577Sig182	ER+/PR+ Signature	Breast Cancer
PMID18425577Sig594	ER-PR-_signature_down	Breast Cancer
PMID18451222Sig5	Five-Gene Molecular Grade Index	Breast Cancer
PMID18498629Sig181	181-Gene Classifier	Breast Cancer
PMID18592372Sig2160	ER-Positive (+) Breast Cancer	Breast Cancer
PMID18593943ERBB2Sig56	Chromosome 17 (ERBB2) Cluster	Breast Cancer
PMID18593943JunFosSig56	Jun-Fos Cluster	Breast Cancer
PMID18593943Sig117	Estrogen Receptor (Luminal) Cluster	Breast Cancer
PMID18593943Sig125	Transcription Cluster	Breast Cancer
PMID18593943Sig130	T-Cell Cluster	Breast Cancer
PMID18593943Sig169	Stromal Cluster	Breast Cancer
PMID18593943Sig221	Basal-Like Cluster	Breast Cancer
PMID18593943Sig276	Normal-Like Cluster	Breast Cancer
PMID18593943Sig35	Interferon Cluster	Breast Cancer
PMID18593943Sig58	B-Cell Cluster	Breast Cancer
PMID18593943Sig94	Proliferation Cluster	Breast Cancer
PMID1861214845yoSig50	Age ≤ 45 Years Signature	Breast Cancer
PMID1861214865yoSig50	Age ≥ 65 Years Signature	Breast Cancer
PMID18625725Sig271	Activated TGF-beta Type I Receptor Signature	Breast Cancer
PMID18631401Sig75	Ox-E/ER gene signature	Breast Cancer
PMID18662380Sig20	(HER2 Signaling) ERBB2 Coexpression Module	Breast Cancer
PMID18662380Sig288	[Estrogen Receptor (ER) Signaling] ESR1 Coexpression Module	Breast Cancer
PMID18662380Sig355	(Proliferation) AURKA Coexpression Module	Breast Cancer
PMID18684329Sig127	Consensus Signature	Breast Cancer
PMID18698033Sig10	CASP3 (Apoptosis) Module	Breast Cancer
PMID18698033Sig14	VEGF (Angiogenesis) Module	Breast Cancer
PMID18698033Sig229	AURKA (Proliferation) Module	Breast Cancer
PMID18698033Sig28	ERBB2 (HER2 Signaling) Module	Breast Cancer
PMID18698033Sig469	ESR1 (ER Signaling) Module	Breast Cancer
PMID18698033Sig68	PLAU (Tumor Invasion) Module	Breast Cancer
PMID18698033Sig95	STAT1 (Immune Response) Module	Breast Cancer
PMID18714348Sig22	22-Gene 3D-Signature	Breast Cancer
PMID18757322Sig1084	IGF-I Signature	Breast Cancer
PMID18786252Sig26	Cell Cycle Gene Signature	Breast Cancer
PMID18812439Sig15	miRNA Signature	Breast Cancer
PMID18812439Sig52	miRNA Targets Signature	Breast Cancer

PMID19010862Sig205	IBC Signature	Breast Cancer
PMID19010929Sig41	41 Breast Cancer–Specific ASEs (Alternative Splicing Events)	Breast Cancer
PMID19014521Sig159	Immune Module	Breast Cancer
PMID19014521Sig41	RNA Splicing Module	Breast Cancer
PMID19014521Sig72	expression Grade Index [25]	Breast Cancer
PMID19043454Sig217	217-Gene SAM List	Breast Cancer
PMID19101988Sig18	Expression Levels of Genes that are Potentially Involved in the Maintenance of Genomic Stability	Breast Cancer
PMID19101988Sig38	Commonly Differentially Expressed Genes for the Comparisons of dGS, aGS, and aGU Breast Carcinomas	Breast Cancer
PMID19112599Sig40	Molecular Signature of Metastasis	Breast Cancer
PMID19204204Sig50	PAM50	Breast Cancer
PMID19225562Sig19	Tumor Stroma of African-American Patients Independent of the Tumors' ER-Status	Breast Cancer
PMID19225562Sig30	Tumor Epithelium of the ER-Negative Tumors of African-American Patients	Breast Cancer
PMID19225562Sig46	Tumor Epithelium of the ER-Positive Tumors of African-American Patients	Breast Cancer
PMID19225562Sig485	LCM-Dissected Tumor Epithelium of African-American Breast Cancer Patients	Breast Cancer
PMID19225562Sig8	Tumor Epithelium of African-American Patients Independent of the Tumors' ER-Status	Breast Cancer
PMID19249242Sig36	Exons Located in Unchanged Genes that are Overexpressed in Malignant Tumors with High Splice Indices in Cancer	Breast Cancer
PMID19249242Sig71	Genes Overexpressed (More than Two Times) in Breast Cancer Compared with Benign Lesions	Breast Cancer
PMID19266279Sig16	Malignancy-Risk Genes with p Value < 0.05	Breast Cancer
PMID19266279Sig30	30 Selected Malignancy-Risk Signature Genes	Breast Cancer
PMID19266279Sig37	Subset of Malignancy-Risk Genes Associated with DNA Replication, Mitosis, and Cancer Risk	Breast Cancer
PMID19435916Sig29	Claudin-Low Gene Cluster	Breast Cancer
PMID19435916Sig929	MBC versus Common Breast Tumor Expression Signature	Breast Cancer
PMID19455244Sig5	Tumor Metastasis Signature	Breast Cancer
PMID19497124Sig81	High-Dose Hyperfractionated Radical Radiotherapy Signature	Breast Cancer
PMID19666588Sig493	CD44^{+}/CD24$^{-/low}$-Mammosphere Gene Signature	Breast Cancer
PMID19723662Sig12	Probesets Used for Molecular Subtype Assignment	Breast Cancer
PMID19724859Sig60	Residual Breast Cancer before Doxorubicin and Cyclophosphamide Neoadjuvant Chemotherapy	Breast Cancer
PMID19755993Sig28	Trastuzumab Response Signature	Breast Cancer
PMID19967557Sig23	23-Gene Signature which Predicts Response of Basal-Like Breast Cancer to Neoadjuvant Chemotherapy	Breast Cancer
PMID20068109Sig184	Good Prognosis Signature	Breast Cancer
PMID20068109Sig271	Poor Prognosis Signature	Breast Cancer
PMID20078854Sig160	breast cancer signature	Breast Cancer
PMID20098429Sig12	8q22 Amplification Signature	Breast Cancer
PMID20156340Sig22	HER2+ Subtype Signature	Breast Cancer

PMID20156340Sig229	ER+/HER2- Subtype Signature	Breast Cancer
PMID20156340Sig63	ER-/HER2- Subtype Signature	Breast Cancer
PMID20158880Sig10	Selected Non-Redundant Survival-Significant Gene Pairs Identified in Both Cohorts of Breast Cancer Patients	Breast Cancer
PMID20158880Sig4	Individual Genes Selected among the TNFAIP1/POLDIP2 SFGM (Structural-Functional Gene Module) and 6 "Neighboring" Genes which Proved to be Survival Significant in at Least One Cohort (P Value ≤ 0.05)	Breast Cancer
PMID20158880StockholmSig11	P Values of Kolmogorov-Smirnov Test of Normality in Stockholm cohort for 5 Genes in SFGM Group (SFGM Matrix) and 6 "Neighboring" Genes	Breast Cancer
PMID20158880StockholmSig17	P Values of Kolmogorov-Smirnov Test of Normality in Stockholm Cohort for 17 Genes of ERBB2 Amplicon on 17q12	Breast Cancer
PMID20158880UppsalaSig11	P Values of Kolmogorov-Smirnov Test of Normality in Uppsala cohort for 5 Genes in SFGM Group (SFGM Matrix) and 6 "Neighboring" Genes	Breast Cancer
PMID20158880UppsalaSig17	P Values of Kolmogorov-Smirnov Test of Normality in Uppsala Cohort for 17 Genes of ERBB2 Amplicon on 17q12	Breast Cancer
PMID20368555Sig1022	GSE3494 ER Predictor Gene List	Breast Cancer
PMID20368555Sig205	GSE1456 Recurrence Predictor Gene List	Breast Cancer
PMID20368555Sig50	Docetaxel Chemosensitivity Signature Gene List	Breast Cancer
PMID20368555Sig52	Adriamycin Chemosensitivity Signature Gene List	Breast Cancer
PMID20490655Sig368	368-Gene SVM Model	Breast Cancer
PMID20521089Sig35	Genes that Demonstrate Significantly Correlated or Anti-Correlated Expression with CD24 across the Basal B Cell Lines	Breast Cancer
PMID20521089Sig55	Basal B Breast Cancer Cell Line Subtype	Breast Cancer
PMID20521089Sig57	Genes that Demonstrate Correlated or Anti-Correlated Expression with CD24 across the Basal B Cell Lines	Breast Cancer
PMID20521089Sig80	Occurrence of the Epithelial-Mesenchymal Transition in Breast Cancer Cell Lines	Breast Cancer
PMID20697068Sig165	Sensitivity to Endocrine Therapy (SET) Index	Breast Cancer
PMID20713713Sig251	EMT Core Signature	Breast Cancer
PMID20813035Sig1048	$CD49f^{+}/EpCAM^{-/low}$ SUM149PT (Basal-Like) Cells Fraction Signature	Breast Cancer
PMID20813035Sig1177	Normal Breast-Like Tumor Signature	Breast Cancer
PMID20813035Sig1275	Luminal Tumor Signature	Breast Cancer
PMID20813035Sig137	Cell Adhesion Genes in Claudin-Low Tumors Signature	Breast Cancer
PMID20813035Sig1447	Basal-Like Tumor Signature	Breast Cancer
PMID20813035Sig1667	Claudin-Low Tumor Signature	Breast Cancer
PMID20813035Sig19	Luminal Gene Cluster	Breast Cancer
PMID20813035Sig26	Claudin-Low versus Basal-Like Signature	Breast Cancer
PMID20813035Sig41	Proliferation Gene Cluster	Breast Cancer
PMID20813035Sig509	HER2-Enriched Tumor Signature	Breast Cancer
PMID20813035Sig807	Nine-Cell Line Claudin-Low Predictor	Breast Cancer
PMID20932292Sig12	Genes Targeted by the 17q12-q21-Amplicon in IBCs and NIBCs	Breast Cancer

PMID20932292Sig37	Genes whose Expression Levels were Downregulated and Upregulated in Proportion to DNA Copy Number Losses and Gains (or Amplification), Respectively	Breast Cancer
PMID20932292Sig402	Genes with Expression Significantly Different in ER- and ER+ ERBB2-Amplified Tumors	Breast Cancer
PMID20932292Sig43	Copy Number Aberration (CNA) Signature	Breast Cancer
PMID21176237Sig12	Oncotype DX Colon Cancer Assay	Breast Cancer
PMID21501481Sig15	Tumor Vascular Endothelial Normalization Signature	Breast Cancer
PMID21501481Sig18	Cell Cycle and Proliferation Signature	Breast Cancer
PMID21501481Sig21	Stromal Reaction Signature	Breast Cancer
PMID21501481Sig224	Wound Response Signature	Breast Cancer
PMID21558518Sig26	Early Relapse in ER-Negative Breast Cancer Signature	Breast Cancer
PMID21558518Sig32	Early Relapse in ER-Positive Breast Cancer Signature	Breast Cancer
PMID21558518Sig37	Excellent Pathologic Response in ER-Positive Breast Cancer Signature	Breast Cancer
PMID21558518Sig51	Extensive Residual Disease in ER-Negative Breast Cancer Signature	Breast Cancer
PMID21558518Sig52	Excellent Pathologic Response in ER-Negative Breast Cancer Signature	Breast Cancer
PMID21558518Sig73	Extensive Residual Disease in ER-Positive Breast Cancer Signature	Breast Cancer
PMID23383020BRmet50	a meta-signature in breast cancer for poor prognosis prediction	Breast Cancer
PMID16704732Sig222	T-Cell Signature	cell type specific
PMID16704732Sig23	CD8+ T-Cell Signature	cell type specific
PMID16704732Sig411	Granulocyte Signature	cell type specific
PMID16704732Sig427	B-Cell Signature	cell type specific
PMID16704732Sig67	Lymphocyte Signature	cell type specific
PMID11707567CMSig25	Colon Metastasis Signature	Colon cancer
PMID17255271Sig19	German 19-Gene Classifier	Colon cancer
PMID17255271Sig22	New Zealand 22-Gene Classifier	Colon cancer
PMID19050079Sig50	50-Gene Predictor of Recurrence in Early Stage Colon Carcinoma	Colon cancer
PMID19914252Sig34	34-Gene Recurrence Classifier	Colon cancer
PMID18470533Sig31	Thirty-One Schizophrenia Susceptibility Genes	Complex neuropsychiatric disorders
PMID18599519Sig10	gene cluster 2 from Type 1 Diabetes Monocytes	Diabets
PMID18599519Sig12	gene cluster 1 from Monocytes of Bipolar Patients	Diabets
PMID11807556Sig100	Supervised Learning Outcome Predictor	Embryonal tumours of the central nervous system (CNS)
PMID14522886Sig66	Genes Differentially Expressed in Endometrial Carcinomas	Endometrial Carcinomas
PMID11965276Sig125	Gastrointestinal Stromal Tumor Gene Cluster	Gastrointestinal Stromal Tumors
PMID18714668Sig1658	Preliminary Molecular Signature of Acute GVHD in Allogeneic HSCT Patients	GVHD
PMID16467079Sig91	91 Top Classifiers for HPV Status by Significance	Head and neck

	Analysis of Microarrays	cancer
PMID17409455Sig126	HS-up (Hypoxia Score of the up-regulated genes)	Head and neck cancer
PMID17409455Sig68	HS-down (Hypoxia Score of the down-regulated genes)	Head and neck cancer
PMID12648972Sig12	Scoring System for Early Intrahepatic Recurrence	Hepatocellular carcinoma
PMID15897992Sig10	HIV Serostatus Predictor Set	HIV-1 disease
PMID15897992Sig6	6-Gene Signature Set that can be Used to Distinguish Samples from Seropositive Persons with Decreasing or Increasing CD4+ T Cell Counts	HIV-1 disease
PMID15930270Sig16	Tumor-Forming Genetic Signature (Genes Induced in a Highly Synergistic Manner Only when Both H-Ras and p53 Inactivation Occurred)	Human fibroblasts oncogenci alternation
PMID15930270Sig38	Inactivated p53-Associated Proliferation Signature	Human fibroblasts oncogenci alternation
PMID10521349Sig50	AML-ALL Class Predictor	Leukemia
PMID12086872ALLSig20	Discriminator of Relapse versus CCR in HD Cases	Leukemia
PMID12086872AMLSig20	Predictor of the Development of Secondary AML in TEL-AML1-Positive ALLs	Leukemia
PMID12086872Sig7	Discriminator of Relapse versus CCR in T-ALL Cases	Leukemia
PMID12738660Sig35	35 Prognosis-Associated Genes	Leukemia
PMID15084693Sig133	133-Gene Outcome Predictor	Leukemia
PMID15084694Sig180	PAM Genes of Prognostically Important Clusters (#13, #12, #9, #16, #10, #4, #15, #4 and #15, and FLT3ITD)	Leukemia
PMID20166207Sig82	GEP of CLL-CECs and nCECs	Leukemia
PMID20564080Sig16	PK Genes Specifically Expressed in B-NEG Patients Versus at Least 2 Other Groups	Leukemia
PMID20564080Sig53	PK Genes Distinctive of Each ALL Subgroup	Leukemia
PMID19514085Sig73	73-Gene Signature that Correlated with Vascular Invasion	Liver cancer
PMID11707567C1Sig25	Cluster C1 (Proliferation-Related Gene Expression)	Lung cancer
PMID11707567C2Sig25	Cluster C2 (Neuroendocrine Gene Expression, Ornithine Decarboxylase 1 and Surfactant Gene Expression)	Lung cancer
PMID11707567C3Sig25	Cluster C3 (Ornithine Decarboxylase 1 and Surfactant Gene Expression, Type II Pneumocyte Gene Expression)	Lung cancer
PMID11707567C4Sig25	Cluster C4 (Type II Pneumocyte Gene Expression)	Lung cancer
PMID11707567GroupISig25	Group I Signature	Lung cancer
PMID11707590Sig20	Lung cancer adenocarcinomas signature group 2	Lung cancer
PMID11707590Sig217	Lung cancer adenocarcinomas signature all groups	Lung cancer
PMID11707590Sig26	Small Cell Tumors	Lung cancer
PMID11707590Sig33	Squamous Lung Tumors	Lung cancer
PMID11707590Sig38	Large Cell Tumors	Lung cancer
PMID11707590Sig39	Lung cancer adenocarcinomas signature group 1	Lung cancer
PMID11707590Sig90	Lung cancer adenocarcinomas signature group 3	Lung cancer
PMID12036904Sig22	Statistically Significant Genes for DFS from Cox Proportional Hazards Model Testing	Lung cancer
PMID12118244Sig100	100 Survival-Related Genes from low risk cancer	Lung cancer

	group for lung adenocarcinoma	
PMID14504202Sig7	Lung Cancer Prognosis Signature	Lung cancer
PMID14871811LACSig29	Lung Adenocarcinomas	Lung cancer
PMID14871811LSCCSig48	Lung Squamous Cell Carcinomas	Lung cancer
PMID16049480Sig18	Lung and Bone Metastasis	Lung cancer
PMID16899777mgene19Sig14	Metagene 19	Lung cancer
PMID16899777mgene31Sig13	Metagene 31	Lung cancer
PMID16899777mgene35Sig5	Metagene 35	Lung cancer
PMID16899777mgene40Sig26	Metagene 40	Lung cancer
PMID16899777mgene41Sig16	Metagene 41	Lung cancer
PMID16899777mgene69Sig14	Metagene 69	Lung cancer
PMID16899777mgene74Sig11	Metagene 74	Lung cancer
PMID16899777mgene79Sig19	Metagene 79	Lung cancer
PMID16899777mgene86Sig15	Metagene 86	Lung cancer
PMID17194181Sig64	64-Gene Expression Signature	Lung cancer
PMID17202451Sig16	16-Gene Signature	Lung cancer
PMID17202451Sig5	Five-Gene Signature	Lung cancer
PMID18676831Sig6	Six-Gene Lung Metastasis Signature	Lung cancer
PMID20233996Sig3	Three-Gene Signature of Lau et al for lung cancer prognosis	Lung cancer
PMID20233996Sig5	Five-Gene Signature of Chen et al for lung cancer prognosis	Lung cancer
PMID12075054Sig17	Poor prognosis signature in large-B-cell lymphoma	Lymphoma
PMID14961037Sig120	120 Genes whose Expression Patterns Differed between Reactive Lymph Node Tissue and Three Types of SBCL: Follicular Lymphoma, Mantle Cell Lymphoma, and Chronic Lymphocytic Leukemia/Small Lymphocytic Lymphoma	Lymphoma
PMID15115829Sig6	Six-Gene Model	Lymphoma
PMID16760443Sig21	c-myc and Target Genes	Lymphoma
PMID16760443Sig23	NF-κB Target Genes	Lymphoma
PMID16760443Sig49	Germinal-Center B-Cell Genes	Lymphoma
PMID16760443Sig6	MHC Class I Genes	Lymphoma
PMID12756227Sig79	Genes expresed in mammosphere-derived cells only	Mammary stem cells
PMID18591436Sig45	Transcriptome-Based Biomarker (TBB) that Predicts Long-Term Clinical Outcome in Patients with New-Onset Heart Failure	New-onset heart failure (HF)
PMID17079134Sig38	Genes Found Differentially Expressed in HPV-Positive SCCHN versus Normal Oral Epithelium	Oral cancer
PMID17079134Sig39	Genes Found Differentially Expressed in HPV-Negative SCCHN versus Normal Oral Epithelium	Oral cancer
PMID15643601Sig11	Pancreatic Carcinoma Metastasis	Pancreatic cancer
PMID12154050Sig80	Cohort of Cross-Validated Genes Identified by Meta-Analysis of Prostate Cancer Gene Expression Profiles	Prostate cancer
PMID15067324Sig218	Recurrent Human Prostate Tumors	Prostate cancer
PMID18006806Sig14	Polycomb Repression Signature	Prostate cancer
PMID18505969Sig87	87-Gene TMPRSS2–ERG Signature	Prostate cancer
PMID18846227Sig17	17 Gene/Probe Model	Prostate cancer
PMID14871811RCCSig39	Renal Cell Carcinomas	Renal cancer

PMID12469122Sig17	17-Gene Signature Associated with Metastasis	Solid tumor
PMID19658182Sig61	Sixty-One Genes Differentially Expressed Between Benign, Borderline, and/or Malignant Thyroid Lesions	Thyroid cancer
PMID14527998Sig249	Normal Old Cells and Werner Syndrome Cells	Werner syndrome (WS)

Table S2. List of validation datasets in breast cancer

PubMed	Datasets	Total probe number	Total sample number	Clinical endpoints	N/A rate	Differential index
21501481	**GSE20685**	54619	327	OS	0%	76%
16141321	**BR1128**	22215	249	DSS	0%	66%
18593943	**GSE11121**	22215	200	DMFS	0%	65%
20490655	**GSE21653**	54613	266	DFS	0%	61%
17079448	**BR1095**	22215	289	DFS	0%	60%
17079448	**BR1414**	22215	147	RFS, DFS	0%	54%
22522925	**BRMetabricV22522925**	48803	995	DSS, OS	0%	53%
22522925	**BRMetabricD22522925**	48803	997	DSS, OS	0%	53%
15721472	**BR1405**	23255	286	RFS	0%	48%
21558518	**GSE25055**	22215	310	DMFS	0%	46%
16505416	**BR1224**	20024	40	RFS	0%	43%
12490681	**NKI295 BR2411**	24481	295	RFS, DFS, DMFS	0%	43%
18498629	**BR1141**	22215	275	RFS, DMFS	0%	40%
11823860	**BR544**	19517	97	DMFS	0%	39%
17545524	**GSE7390**	22215	198	RFS,DMFS,OS	0%	36%
21558518	**GSE25065**	22215	198	DMFS	0%	34%
16643655	**BR1552**	22575	69	RFS	2%	32%
19204204	**GSE10886**	20835	197	RFS,OS	0%	30%
20697068	**GSE17705**	22215	298	DMFS	0%	28%
16626501	**GSE2607GPL887**	20024	50	RFS, OS	0%	17%
16478745	**BR1042**	22215	172	RFS	0%	15%
21115855	**GSE20624**	20835	293	RFS	0%	9%
18347175	**BR18347175**	21521	155	RFS, DFS	0%	6%
20813035	**GSE18229**	20835	199	RFS, OS	4%	4%
18592372	GSE10510	26581	148	OS, DFS	38%	1%
17663798	BR17663798	22575	191	RFS,DFS	42%	-1%
20098429	GSE16446	54613	120	DMFS, OS	0%	-1%
16626501	GSE2607GPL1390	20835	64	RFS, OS	68%	-3%
18782450	BR907	20024	94	RFS, DFS	56%	-3%
19435916	GSE10885	20835	119	RFS, OS	4%	-15%
20932292	GSE17907	24577	55	DMFS	0%	-19%

Table S3. Identification of prognostic signature candidates in breast cancer

Signature ID	% Significant p values	Signature description
PMID23383020BRmet50	74%	A meta-signature in breast cancer for poor prognosis prediction
PMID18271932Sig33	68%	Predictor Gene Set for TP53 Status
PMID18360352Sig21	65%	Oncotype DX Breast Cancer Assay
PMID18662380Sig355	65%	(Proliferation) AURKA Coexpression Module
PMID18231641Sig73	61%	73-Gene Outcome Signature
PMID16626501Sig53	61%	53 Classifier Genes
PMID15899795Sig50	61%	Breast cancer cell proliferation signature
PMID16280042Sig64	58%	64-Gene Set
PMID17885619Sig143	58%	Aromatase Inhibitor Letrozole Signature
PMID18684329Sig127	58%	Consensus Signature
PMID18425577Sig182	58%	ER+/PR+ Signature
PMID15598354Sig90	58%	90-Gene Meta-Signature
PMID18498629Sig181	58%	181-Gene Classifier
PMID15721472Sig76	58%	76-Gene Prognostic Signature (for Development of Metastasis)
PMID17150101Sig52	55%	p53 Loss Signature
PMID20156340Sig229	55%	ER+/HER2- Subtype Signature
PMID18698033Sig229	55%	AURKA (Proliferation) Module
PMID18347175Sig36	55%	36-Gene PAM Signature
PMID18338247Sig736	55%	Genes Expressed Differentially in Nonresponders Compared to Responders
PMID12714683Sig29	55%	Cluster of 29 Genes Showing Consistent Up-Regulation in IDC
PMID11707567C1Sig25	55%	Cluster C1 (Proliferation-Related Gene Expression)
PMID17160137Sig59	55%	59 RB (Retinoblastoma Tumor Suppressor) Target Genes
PMID17883867Sig60	52%	60-Gene Profile for recurrence
PMID17079448Sig264	52%	PAM 264-Gene Classifier
PMID17076897Sig52	52%	Consensus Prognostic Gene Expression Classifier for ER-Positive (+) Breast Cancer
PMID12714683Sig85	52%	85 Genes with Increased Expression in IDC
PMID20368555Sig1022	52%	GSE3494 ER Predictor Gene List
PMID19723662Sig12	52%	Probesets Used for Molecular Subtype Assignment
PMID19225562Sig485	52%	LCM-dissected breast cancer signatures
PMID16491069Sig45	52%	Breast Cancer Proliferation Cluster
PMID16505416Sig822	52%	Estrogen-SAM List
PMID21501481Sig18	52%	Cell Cycle and Proliferation Signature
PMID19014521Sig72	52%	Cancer proliferation gene set
PMID21501481Sig224	52%	Wound Response Signature
PMID11823860Sig70	52%	MammaPrint for metastasis prediction
PMID18593943Sig117	52%	Estrogen Receptor (Luminal) Cluster
PMID16091131Sig31	52%	Preoperative Systemic Chemotherapy (PST) with Epirubicin/Cyclophosphamide (EC) Favorable Outcome Signature

Table S4. Annotation of genes in BRmet50.

Gene Symbol	Expression in Cancer	Role in Cancer	Function Category
UBE2C	Up[1, 2]	Progression[1, 2]	Cell Cycle
KIF2C	Up[3]	Progression[4]	Cell Cycle
TACC3	Up[5]	Progression[5]	Cell Cycle
MAD2L1	Up[6]	Progression[6]	Cell Cycle
AURKA	Up[7]	Progression[7]	Cell Cycle
CEP55	Up[8]	Progression[9]	Cell Cycle
CCNB1	Up[10]	Progression[11]	Cell Cycle
RRM2	Up[12, 13]	Progression[14]	Cell Cycle
DLGAP5	Up[15]	Progression[15]	Cell Cycle
NEK2	Up[16]	Progression[17]	Cell Cycle
NDC80	Up[18]	NA*	Cell Cycle
UBE2S	Up[19]	NA	Cell Cycle
CCNB2	Up[20]	NA	Cell Cycle
KIF20A	Up[21]	NA	Cell Cycle
TRIP13	Up[22]	NA	Cell Cycle
CDKN3	Up[23]	NA	Cell Cycle
RAD51	Up[24]	Progression[24]	DNA Replication
KPNA2	Up[25]	Progression[25]	DNA Replication
TYMS	Up[26]	NA	DNA Replication
CDT1	Up[27]	NA	DNA Replication
FEN1	Up[28]	NA	DNA Replication
RFC4	Up[29]	NA	DNA Replication
EZH2	Up[30]	Progression[30]	Proliferation
DDX39	Up[31]	Progression[31]	Proliferation
GTPBP4	Down[32]	Suppressor[32]	Proliferation
CCT5	Up[33]	NA	Protein Folding
HJURP	NA	NA	Cell Cycle
SPAG5	NA	NA	Cell Cycle
KIF4A	NA	NA	Cell Cycle
PRC1	NA	NA	Cell Cycle
KIF23	NA	NA	Cell Cycle
NUSAP1	NA	NA	Cell Cycle
CENPN	NA	NA	Cell Cycle
LRP8	NA	NA	Cell Movement
GMPS	NA	NA	DNA Replication
MCM10	NA	NA	DNA Replication
CDC45L	NA	NA	DNA Replication
GARS	NA	NA	Proliferation
C1orf106	NA	NA	NA
BTG2	Down[34]	Suppressor[34]	Anti-Proliferation
SCUBE2	Down[35]	Suppressor[35]	Anti-Proliferation
OGN	Down[36]	NA	Cellular Assembly
SH3BGRL	Down[37]	NA	Thioredoxin Fold Proteins
COL14A1	NA	NA	Cellular Assembly
SPARCL1	NA	NA	Cellular Assembly

RAI2	NA	NA	Proliferation
KIF13B	NA	NA	Cell Movement
QDPR	NA	NA	Aamino Acid and Oxidation
ALDH3A2	NA	NA	Lipid Oxidoreductase Activity
CIRBP	NA	NA	mRNA Stabilization

*Up for up-regulation; Down for down-regulation; NA for not available.
Meta-direction: concordant expression direction of the BRmet50 genes.

Table S5. Annotation of genes in PMID18271932Sig33

Gene Symbol	Expression in Cancer	Role in Cancer	Function Category
CCNB2*	Up[38]	Oncogene[38]	Cell cycle
CDC45	Up[39]	Growth[39]	DNA replication
CEP55	Up[8]	Antigen[8]	Cell cycle
KIF23	Up[40]	Growth[40]	Cell cycle
KIF2C	UP[41]	Progression[41]	Cell cycle
PRC1	Up[42]	Progression[42]	Cell cycle
UBE2C	Up[43]	Progression[44]	Ubiquitination
ASPM	Up[45]	Progression[45]	Cell cycle
BCL11A	Up[46]	Oncogene[46]	B cell development
BIRC5	Up[47]	Apoptosis inhibition[47]	Apoptosis
CDCA8	Up[48]	Growth[48]	Cell cycle
CENPF	Up[49]	Oncogene[50]	Cell cycle
HEXIM1	Up[51]	Progression[51]	Cell cycle
MAPRE1	Up[52]	Oncogene[52]	Cell cycle
PLK1	Up[53]	Progression[53]	Cell cycle
PTP4A2	Up[54]	Oncogene[54] Up	Proliferation
PTTG1	Up[55]	Oncogene[55]	Cell cycle
STMN1	Up[56]	Progression[57]	Cellular Assembly
SULF2	Up[58]	Progression[58]	Cell movement
UBE2T	Up[59]	Progression[60]	Ubiquitination
CENPE	Down[61]	Growth[61]	Cell cycle
LIN9	Down[62]	Suppressor[62]	DNA replication
RPS27L	Down[63]	Growth[64]	Apoptosis
TMEM25	Down[65]	Biomarker[65]	NA
C15orf42	NA	NA	NA
C3orf18	NA	NA	NA
FAM63A	NA	NA	NA
FANCI	NA	NA	DNA repair
MKNK2	NA	NA	Proliferation
MUTYH	NA	NA	DNA repair
PKMYT1	NA	NA	Cell cycle
RILPL2	NA	NA	NA

* Seven common gene symbols between BRmet50 and PMID18271932Sig33 are underlined.

Table S6. TP53, ER, PR, and HER2 statuses in TCGA tumor samples.

TCGA Sample ID	TP53 Mutation	TP53 Color	ER	PR	HER2	SUBTYPE	SUBTYPE Color
TCGA-AC-A23C	R174fs	RED	Positive	Positive	Positive	ER/HER2	PURPLE
TCGA-E2-A15E	H193R	RED	Positive	Positive	Positive	ER/HER2	PURPLE
TCGA-E9-A1N5	H193R	RED	Positive	Positive	Positive	ER/HER2	PURPLE
TCGA-A2-A0EY	Y234C	RED	Positive	Negative	Positive	ER/HER2	PURPLE
TCGA-D8-A1X5	C238F	RED	Positive	Positive	Positive	ER/HER2	PURPLE
TCGA-D8-A1XA	R306*	RED	Positive	Positive	Equivocal	ER/HER2	PURPLE
TCGA-BH-A1FE	N247I	RED	Positive	Positive	NA	ER/HER2	PURPLE
TCGA-BH-A18H	Q331*	RED	Positive	Positive	Positive	ER/HER2	PURPLE
TCGA-A8-A08H	WT	WHITE	Positive	Positive	Positive	ER/HER2	PURPLE
TCGA-A8-A07P	WT	WHITE	Positive	Positive	Positive	ER/HER2	PURPLE
TCGA-D8-A140	WT	WHITE	Positive	Positive	Positive	ER/HER2	PURPLE
TCGA-GM-A2DA	WT	WHITE	Positive	Positive	Positive	ER/HER2	PURPLE
TCGA-B6-A0WZ	WT	WHITE	Positive	Positive	NA	ER/HER2	PURPLE
TCGA-B6-A0IP	WT	WHITE	Positive	Positive	NA	ER/HER2	PURPLE
TCGA-AQ-A1H2	WT	WHITE	Positive	Positive	Positive	ER/HER2	PURPLE
TCGA-A8-A099	WT	WHITE	Positive	Positive	Positive	ER/HER2	PURPLE
TCGA-A8-A08C	WT	WHITE	Positive	Positive	Positive	ER/HER2	PURPLE
TCGA-B6-A0IC	WT	WHITE	Positive	Positive	NA	ER/HER2	PURPLE
TCGA-C8-A133	WT	WHITE	Positive	Positive	NA	ER/HER2	PURPLE
TCGA-B6-A0X4	WT	WHITE	Positive	Positive	NA	ER/HER2	PURPLE
TCGA-B6-A0WS	WT	WHITE	Positive	Positive	NA	ER/HER2	PURPLE
TCGA-AR-A1AX	WT	WHITE	Positive	Positive	Positive	ER/HER2	PURPLE
TCGA-A8-A0AB	WT	WHITE	Positive	Positive	Positive	ER/HER2	PURPLE
TCGA-C8-A132	WT	WHITE	Positive	Positive	NA	ER/HER2	PURPLE
TCGA-B6-A0WT	WT	WHITE	Positive	Positive	NA	ER/HER2	PURPLE
TCGA-BH-A18I	WT	WHITE	Positive	Positive	Positive	ER/HER2	PURPLE
TCGA-BH-A18M	WT	WHITE	Positive	Positive	Positive	ER/HER2	PURPLE
TCGA-B6-A0IH	WT	WHITE	Positive	Positive	NA	ER/HER2	PURPLE
TCGA-A8-A08T	WT	WHITE	Positive	Positive	Positive	ER/HER2	PURPLE
TCGA-AN-A0FK	WT	WHITE	Positive	Positive	Positive	ER/HER2	PURPLE
TCGA-E2-A1B1	WT	WHITE	Positive	Positive	Positive	ER/HER2	PURPLE
TCGA-E2-A1IH	WT	WHITE	Positive	Positive	Equivocal	ER/HER2	PURPLE
TCGA-D8-A1JB	WT	WHITE	Positive	Positive	Positive	ER/HER2	PURPLE
TCGA-AN-A041	WT	WHITE	Positive	Negative	Positive	ER/HER2	PURPLE
TCGA-AN-A03X	WT	WHITE	Positive	Positive	Positive	ER/HER2	PURPLE
TCGA-E9-A22E	WT	WHITE	Positive	Positive	Positive	ER/HER2	PURPLE
TCGA-B6-A0I5	WT	WHITE	Positive	Positive	NA	ER/HER2	PURPLE
TCGA-B6-A0X0	WT	WHITE	Positive	Positive	NA	ER/HER2	PURPLE
TCGA-A8-A08S	WT	WHITE	Positive	Positive	Positive	ER/HER2	PURPLE
TCGA-AN-A0XP	WT	WHITE	Positive	Positive	Positive	ER/HER2	PURPLE
TCGA-BH-A1FL	WT	WHITE	Positive	Positive	NA	ER/HER2	PURPLE
TCGA-A7-A26H	WT	WHITE	Positive	Negative	Positive	ER/HER2	PURPLE
TCGA-AN-A0FS	WT	WHITE	Positive	Negative	Positive	ER/HER2	PURPLE

TCGA-BH-A1FR	WT	WHITE	Positive	Positive	NA	ER/HER2	PURPLE
TCGA-B6-A0RV	WT	WHITE	Positive	Positive	NA	ER/HER2	PURPLE
TCGA-E9-A295	WT	WHITE	Positive	Positive	Positive	ER/HER2	PURPLE
TCGA-A8-A06T	WT	WHITE	Positive	Positive	Positive	ER/HER2	PURPLE
TCGA-B6-A0RP	WT	WHITE	Positive	Positive	NA	ER/HER2	PURPLE
TCGA-C8-A1HE	WT	WHITE	Positive	Positive	Equivocal	ER/HER2	PURPLE
TCGA-BH-A0BQ	WT	WHITE	Positive	Positive	Positive	ER/HER2	PURPLE
TCGA-B6-A0X7	WT	WHITE	Positive	Positive	NA	ER/HER2	PURPLE
TCGA-AN-A0FN	WT	WHITE	Positive	Positive	Positive	ER/HER2	PURPLE
TCGA-AC-A23G	WT	WHITE	Positive	Positive	Positive	ER/HER2	PURPLE
TCGA-B6-A0RO	WT	WHITE	Positive	Positive	NA	ER/HER2	PURPLE
TCGA-E9-A1N4	WT	WHITE	Positive	Positive	Positive	ER/HER2	PURPLE
TCGA-A2-A0SY	WT	WHITE	Positive	Positive	Positive	ER/HER2	PURPLE
TCGA-AC-A2FB	WT	WHITE	Positive	Positive	Positive	ER/HER2	PURPLE
TCGA-D8-A1XY	WT	WHITE	Positive	Positive	Positive	ER/HER2	PURPLE
TCGA-BH-A0B6	WT	WHITE	Positive	Positive	Positive	ER/HER2	PURPLE
TCGA-AC-A23E	WT	WHITE	Positive	Positive	NA	ER/HER2	PURPLE
TCGA-E2-A3DX	WT	WHITE	Positive	Positive	Positive	ER/HER2	PURPLE
TCGA-AQ-A0Y5	WT	WHITE	Positive	Positive	Positive	ER/HER2	PURPLE
TCGA-B6-A0RQ	WT	WHITE	Positive	Positive	NA	ER/HER2	PURPLE
TCGA-BH-A0W5	WT	WHITE	Positive	Positive	Equivocal	ER/HER2	PURPLE
TCGA-AR-A1AW	R273C	RED	Positive	Positive	Negative	ER	GREEN
TCGA-E9-A1NF	F113C	RED	Positive	Positive	Negative	ER	GREEN
TCGA-BH-A0C3	G187S	RED	Positive	Negative	Negative	ER	GREEN
TCGA-AN-A046	E11K	RED	Positive	Positive	Negative	ER	GREEN
TCGA-BH-A1EY	Y126C	RED	Positive	Positive	Negative	ER	GREEN
TCGA-AR-A1AN	R110fs	RED	Positive	Positive	Negative	ER	GREEN
TCGA-BH-A0B0	R175H	RED	Positive	Positive	Negative	ER	GREEN
TCGA-A7-A3RF	R273H	RED	Positive	Positive	Negative	ER	GREEN
TCGA-BH-A0BT	R158G	RED	Positive	Positive	Negative	ER	GREEN
TCGA-AR-A1AS	R175H	RED	Positive	Positive	Negative	ER	GREEN
TCGA-BH-A0BC	R248W	RED	Positive	Positive	Negative	ER	GREEN
TCGA-AR-A1AP	R196*	RED	Positive	Positive	Negative	ER	GREEN
TCGA-E2-A108	R273H	RED	Positive	Positive	Negative	ER	GREEN
TCGA-AN-A0AS	R110del	RED	Positive	Negative	Negative	ER	GREEN
TCGA-AR-A24T	R273H	RED	Positive	Positive	Negative	ER	GREEN
TCGA-E2-A1IN	V157G	RED	Positive	Positive	Negative	ER	GREEN
TCGA-AN-A0XT	Y126_splice	RED	Positive	Negative	Negative	ER	GREEN
TCGA-BH-A0BP	K132N	RED	Positive	Positive	Negative	ER	GREEN
TCGA-E2-A1IO	P190fs	RED	Positive	Positive	Negative	ER	GREEN
TCGA-BH-A0DI	I255N	RED	Positive	Positive	Negative	ER	GREEN
TCGA-D8-A1XR	WT	WHITE	Positive	Positive	Negative	ER	GREEN
TCGA-BH-A0C1	WT	WHITE	Positive	Positive	Negative	ER	GREEN
TCGA-A8-A07F	WT	WHITE	Positive	Positive	Negative	ER	GREEN
TCGA-E2-A15L	WT	WHITE	Positive	Positive	Negative	ER	GREEN
TCGA-BH-A0DG	WT	WHITE	Positive	Negative	Negative	ER	GREEN
TCGA-EW-A1J1	WT	WHITE	Positive	Positive	Negative	ER	GREEN
TCGA-AR-A24O	WT	WHITE	Positive	Positive	Negative	ER	GREEN

TCGA-BH-A1ET	WT	WHITE	Positive	Positive	Negative	ER	GREEN
TCGA-AO-A12B	WT	WHITE	Positive	Positive	Negative	ER	GREEN
TCGA-D8-A1JI	WT	WHITE	Positive	Positive	Negative	ER	GREEN
TCGA-GM-A2DC	WT	WHITE	Positive	Positive	Negative	ER	GREEN
TCGA-C8-A12M	WT	WHITE	Positive	Negative	Negative	ER	GREEN
TCGA-A8-A06Y	WT	WHITE	Positive	Positive	Negative	ER	GREEN
TCGA-AC-A2QI	WT	WHITE	Positive	Negative	Negative	ER	GREEN
TCGA-AN-A0XO	WT	WHITE	Positive	Negative	Negative	ER	GREEN
TCGA-EW-A1PE	WT	WHITE	Positive	Positive	Negative	ER	GREEN
TCGA-E2-A156	WT	WHITE	Positive	Positive	Negative	ER	GREEN
TCGA-AO-A12H	WT	WHITE	Positive	Positive	Negative	ER	GREEN
TCGA-BH-A0DX	WT	WHITE	Positive	Positive	Negative	ER	GREEN
TCGA-A1-A0SQ	WT	WHITE	Positive	Positive	Negative	ER	GREEN
TCGA-D8-A1JN	WT	WHITE	Positive	Positive	Negative	ER	GREEN
TCGA-BH-A0DO	WT	WHITE	Positive	Positive	Negative	ER	GREEN
TCGA-E2-A15I	WT	WHITE	Positive	Positive	Negative	ER	GREEN
TCGA-BH-A18N	WT	WHITE	Positive	Positive	Negative	ER	GREEN
TCGA-A8-A083	WT	WHITE	Positive	Positive	Negative	ER	GREEN
TCGA-BH-A18S	WT	WHITE	Positive	Positive	Negative	ER	GREEN
TCGA-A7-A13G	WT	WHITE	Positive	Positive	Negative	ER	GREEN
TCGA-AO-A0J8	WT	WHITE	Positive	Positive	Negative	ER	GREEN
TCGA-D8-A1JS	WT	WHITE	Positive	Positive	Negative	ER	GREEN
TCGA-BH-A0HI	WT	WHITE	Positive	Positive	Negative	ER	GREEN
TCGA-E2-A15G	WT	WHITE	Positive	Positive	Negative	ER	GREEN
TCGA-D8-A27E	WT	WHITE	Positive	Positive	Negative	ER	GREEN
TCGA-BH-A0DS	WT	WHITE	Positive	Positive	Negative	ER	GREEN
TCGA-E2-A1IK	WT	WHITE	Positive	Positive	Negative	ER	GREEN
TCGA-E2-A1IU	WT	WHITE	Positive	Positive	Negative	ER	GREEN
TCGA-BH-A0HO	WT	WHITE	Positive	Positive	Negative	ER	GREEN
TCGA-D8-A27P	WT	WHITE	Positive	Positive	Negative	ER	GREEN
TCGA-D8-A1XC	WT	WHITE	Positive	Positive	Negative	ER	GREEN
TCGA-AO-A0JC	WT	WHITE	Positive	Positive	Negative	ER	GREEN
TCGA-A1-A0SB	WT	WHITE	Positive	Negative	Negative	ER	GREEN
TCGA-AO-A1KO	WT	WHITE	Positive	Positive	Negative	ER	GREEN
TCGA-A2-A0YC	WT	WHITE	Positive	Positive	Negative	ER	GREEN
TCGA-BH-A1ES	WT	WHITE	Positive	Positive	Negative	ER	GREEN
TCGA-AO-A126	WT	WHITE	Positive	Positive	Negative	ER	GREEN
TCGA-E2-A15K	WT	WHITE	Positive	Positive	Negative	ER	GREEN
TCGA-EW-A1P6	WT	WHITE	Positive	Positive	Negative	ER	GREEN
TCGA-A8-A0A4	WT	WHITE	Positive	Positive	Negative	ER	GREEN
TCGA-AR-A1AU	WT	WHITE	Positive	Positive	Negative	ER	GREEN
TCGA-EW-A1J5	WT	WHITE	Positive	Positive	Negative	ER	GREEN
TCGA-EW-A1P0	WT	WHITE	Positive	Negative	Negative	ER	GREEN
TCGA-AN-A0FF	WT	WHITE	Positive	Positive	Negative	ER	GREEN
TCGA-A1-A0SE	WT	WHITE	Positive	Positive	Negative	ER	GREEN
TCGA-A8-A09K	WT	WHITE	Positive	Positive	Negative	ER	GREEN
TCGA-EW-A1P3	WT	WHITE	Positive	Positive	Negative	ER	GREEN
TCGA-A7-A3IZ	WT	WHITE	Positive	Negative	Negative	ER	GREEN

TCGA-AR-A2LL	WT	WHITE	Positive	Positive	Negative	ER	GREEN
TCGA-BH-A0HW	WT	WHITE	Positive	Negative	Negative	ER	GREEN
TCGA-A8-A08O	WT	WHITE	Positive	Positive	Negative	ER	GREEN
TCGA-A8-A0A9	WT	WHITE	Positive	Positive	Negative	ER	GREEN
TCGA-E2-A15F	WT	WHITE	Positive	Positive	Negative	ER	GREEN
TCGA-D8-A1X7	WT	WHITE	Positive	Positive	Negative	ER	GREEN
TCGA-AO-A0JI	WT	WHITE	Positive	Negative	Negative	ER	GREEN
TCGA-E9-A1R2	WT	WHITE	Positive	Negative	Negative	ER	GREEN
TCGA-A8-A06N	WT	WHITE	Positive	Negative	Negative	ER	GREEN
TCGA-BH-A1F5	WT	WHITE	Positive	Positive	Negative	ER	GREEN
TCGA-D8-A1X8	WT	WHITE	Positive	Indeterminate	Negative	ER	GREEN
TCGA-A8-A0A2	WT	WHITE	Positive	Positive	Negative	ER	GREEN
TCGA-BH-A0DE	WT	WHITE	Positive	Positive	Negative	ER	GREEN
TCGA-AO-A12G	WT	WHITE	Positive	Positive	Negative	ER	GREEN
TCGA-BH-A0AY	WT	WHITE	Positive	Positive	Negative	ER	GREEN
TCGA-A2-A0T7	WT	WHITE	Positive	Positive	Negative	ER	GREEN
TCGA-BH-A0BF	WT	WHITE	Positive	Positive	Negative	ER	GREEN
TCGA-BH-A18K	WT	WHITE	Positive	Positive	Negative	ER	GREEN
TCGA-B6-A0IO	WT	WHITE	Positive	Indeterminate	NA	ER	GREEN
TCGA-A1-A0SF	WT	WHITE	Positive	Positive	Negative	ER	GREEN
TCGA-AR-A2LO	WT	WHITE	Positive	Positive	Negative	ER	GREEN
TCGA-A2-A0YL	WT	WHITE	Positive	Positive	Negative	ER	GREEN
TCGA-AO-A0J5	WT	WHITE	Positive	Negative	Negative	ER	GREEN
TCGA-BH-A0BV	WT	WHITE	Positive	Positive	Negative	ER	GREEN
TCGA-AR-A1AV	WT	WHITE	Positive	Positive	Negative	ER	GREEN
TCGA-AQ-A1H3	WT	WHITE	Positive	Positive	Negative	ER	GREEN
TCGA-D8-A27T	WT	WHITE	Positive	Positive	Negative	ER	GREEN
TCGA-BH-A201	WT	WHITE	Positive	Positive	Negative	ER	GREEN
TCGA-A8-A08Z	WT	WHITE	Positive	Positive	Negative	ER	GREEN
TCGA-AR-A24M	WT	WHITE	Positive	Positive	Negative	ER	GREEN
TCGA-A8-A0A1	WT	WHITE	Positive	Positive	Negative	ER	GREEN
TCGA-D8-A1XB	WT	WHITE	Positive	Positive	Negative	ER	GREEN
TCGA-BH-A18J	WT	WHITE	Positive	Positive	Negative	ER	GREEN
TCGA-BH-A0BA	WT	WHITE	Positive	Positive	Negative	ER	GREEN
TCGA-A8-A0A6	WT	WHITE	Positive	Positive	Negative	ER	GREEN
TCGA-BH-A0BJ	WT	WHITE	Positive	Positive	Negative	ER	GREEN
TCGA-BH-A1EU	WT	WHITE	Positive	Positive	Negative	ER	GREEN
TCGA-AN-A0XL	WT	WHITE	Positive	Positive	Negative	ER	GREEN
TCGA-BH-A0DP	WT	WHITE	Positive	Positive	Negative	ER	GREEN
TCGA-D8-A1XO	WT	WHITE	Positive	Positive	Negative	ER	GREEN
TCGA-BH-A0E9	WT	WHITE	Positive	Positive	Negative	ER	GREEN
TCGA-A7-A26E	WT	WHITE	Positive	Positive	Negative	ER	GREEN
TCGA-D8-A27V	WT	WHITE	Positive	Positive	Negative	ER	GREEN
TCGA-AR-A252	WT	WHITE	Positive	Positive	Negative	ER	GREEN
TCGA-A2-A1FV	WT	WHITE	Positive	Positive	Negative	ER	GREEN
TCGA-BH-A1EX	WT	WHITE	Positive	Positive	Negative	ER	GREEN
TCGA-A1-A0SD	WT	WHITE	Positive	Positive	Negative	ER	GREEN
TCGA-AC-A2B8	WT	WHITE	Positive	Positive	Negative	ER	GREEN

TCGA-BH-A1EO	WT	WHITE	Positive	Positive	Negative	ER	GREEN
TCGA-C8-A1HI	WT	WHITE	Positive	Positive	Negative	ER	GREEN
TCGA-BH-A0GZ	WT	WHITE	Positive	Positive	Negative	ER	GREEN
TCGA-D8-A1X6	WT	WHITE	Positive	Positive	Negative	ER	GREEN
TCGA-AR-A1AK	WT	WHITE	Positive	Positive	Negative	ER	GREEN
TCGA-A2-A0YD	WT	WHITE	Positive	Positive	Negative	ER	GREEN
TCGA-E2-A10F	WT	WHITE	Positive	Positive	Negative	ER	GREEN
TCGA-E2-A1L9	WT	WHITE	Positive	Positive	Negative	ER	GREEN
TCGA-D8-A1JE	WT	WHITE	Positive	Positive	Negative	ER	GREEN
TCGA-E2-A10E	WT	WHITE	Positive	Positive	Negative	ER	GREEN
TCGA-BH-A0H7	WT	WHITE	Positive	Positive	Negative	ER	GREEN
TCGA-AR-A24V	WT	WHITE	Positive	Positive	Negative	ER	GREEN
TCGA-E9-A1NH	WT	WHITE	Positive	Positive	Negative	ER	GREEN
TCGA-C8-A12N	WT	WHITE	Positive	Positive	Negative	ER	GREEN
TCGA-A2-A0T5	WT	WHITE	Positive	Positive	Negative	ER	GREEN
TCGA-AR-A24X	WT	WHITE	Positive	Positive	Negative	ER	GREEN
TCGA-AO-A12A	WT	WHITE	Positive	Positive	Negative	ER	GREEN
TCGA-D8-A27K	WT	WHITE	Positive	Positive	Negative	ER	GREEN
TCGA-BH-A0DT	WT	WHITE	Positive	Positive	Negative	ER	GREEN
TCGA-BH-A28Q	WT	WHITE	Positive	Positive	Indeterminate	ER	GREEN
TCGA-A2-A0CQ	WT	WHITE	Positive	Positive	Negative	ER	GREEN
TCGA-BH-A0DH	WT	WHITE	Positive	Positive	Negative	ER	GREEN
TCGA-BH-A0HA	WT	WHITE	Positive	Positive	Negative	ER	GREEN
TCGA-AO-A0J9	WT	WHITE	Positive	Positive	Negative	ER	GREEN
TCGA-BH-A0W7	WT	WHITE	Positive	Positive	Negative	ER	GREEN
TCGA-GM-A2DK	WT	WHITE	Positive	Negative	Negative	ER	GREEN
TCGA-A8-A09T	WT	WHITE	Positive	Positive	Negative	ER	GREEN
TCGA-EW-A1P5	WT	WHITE	Positive	Positive	Negative	ER	GREEN
TCGA-A7-A3IY	WT	WHITE	Positive	Positive	Negative	ER	GREEN
TCGA-A2-A0SU	WT	WHITE	Positive	Positive	Negative	ER	GREEN
TCGA-AO-A03P	WT	WHITE	Positive	Positive	Negative	ER	GREEN
TCGA-A7-A0CD	WT	WHITE	Positive	Positive	Negative	ER	GREEN
TCGA-EW-A1OX	WT	WHITE	Positive	Positive	Negative	ER	GREEN
TCGA-BH-A0BS	WT	WHITE	Positive	Positive	Negative	ER	GREEN
TCGA-E2-A1L6	WT	WHITE	Positive	Positive	Negative	ER	GREEN
TCGA-D8-A1XV	WT	WHITE	Positive	Positive	Negative	ER	GREEN
TCGA-A8-A093	WT	WHITE	Positive	Positive	Negative	ER	GREEN
TCGA-D8-A1XG	WT	WHITE	Positive	Negative	Negative	ER	GREEN
TCGA-A8-A09V	WT	WHITE	Positive	Positive	Negative	ER	GREEN
TCGA-A8-A096	WT	WHITE	Positive	Positive	Negative	ER	GREEN
TCGA-GM-A2DN	WT	WHITE	Positive	Positive	Negative	ER	GREEN
TCGA-AO-A0JJ	WT	WHITE	Positive	Positive	Negative	ER	GREEN
TCGA-E2-A10B	WT	WHITE	Positive	Positive	Negative	ER	GREEN
TCGA-A2-A0ET	WT	WHITE	Positive	Positive	Negative	ER	GREEN
TCGA-AR-A1AL	WT	WHITE	Positive	Positive	Negative	ER	GREEN
TCGA-A2-A0EM	WT	WHITE	Positive	Positive	Negative	ER	GREEN
TCGA-E2-A1L8	WT	WHITE	Positive	Positive	Negative	ER	GREEN
TCGA-BH-A0H9	WT	WHITE	Positive	Positive	Negative	ER	GREEN

TCGA-AN-A0XV	WT	WHITE	Positive	Positive	Negative	ER	GREEN
TCGA-A7-A3J0	WT	WHITE	Positive	Positive	Negative	ER	GREEN
TCGA-D8-A27G	WT	WHITE	Positive	Positive	Negative	ER	GREEN
TCGA-C8-A12X	WT	WHITE	Positive	Positive	Negative	ER	GREEN
TCGA-A7-A0CG	WT	WHITE	Positive	Negative	Negative	ER	GREEN
TCGA-A2-A1FZ	WT	WHITE	Positive	Positive	Negative	ER	GREEN
TCGA-D8-A27I	WT	WHITE	Positive	Positive	Negative	ER	GREEN
TCGA-A8-A0AD	WT	WHITE	Positive	Positive	Negative	ER	GREEN
TCGA-E2-A14Q	WT	WHITE	Positive	Positive	Negative	ER	GREEN
TCGA-E2-A154	WT	WHITE	Positive	Positive	Negative	ER	GREEN
TCGA-BH-A1FG	WT	WHITE	Positive	Positive	Negative	ER	GREEN
TCGA-E2-A1IL	WT	WHITE	Positive	Positive	Negative	ER	GREEN
TCGA-E2-A15P	WT	WHITE	Positive	Positive	Negative	ER	GREEN
TCGA-E2-A1BD	WT	WHITE	Positive	Positive	Negative	ER	GREEN
TCGA-E2-A1IF	WT	WHITE	Positive	Positive	Negative	ER	GREEN
TCGA-BH-A0HP	WT	WHITE	Positive	Negative	Negative	ER	GREEN
TCGA-A2-A3KD	WT	WHITE	Positive	Positive	Negative	ER	GREEN
TCGA-AO-A125	WT	WHITE	Positive	Positive	Negative	ER	GREEN
TCGA-A8-A09B	WT	WHITE	Positive	Positive	Negative	ER	GREEN
TCGA-E2-A15J	WT	WHITE	Positive	Positive	Negative	ER	GREEN
TCGA-A7-A3J1	WT	WHITE	Positive	Positive	Negative	ER	GREEN
TCGA-AR-A2LE	WT	WHITE	Positive	Negative	NA	ER	GREEN
TCGA-AC-A2FG	WT	WHITE	Positive	Negative	Negative	ER	GREEN
TCGA-A7-A0DB	WT	WHITE	Positive	Positive	Negative	ER	GREEN
TCGA-D8-A1XU	WT	WHITE	Positive	Positive	Negative	ER	GREEN
TCGA-BH-A0HQ	WT	WHITE	Positive	Positive	Negative	ER	GREEN
TCGA-D8-A146	WT	WHITE	Positive	Positive	Negative	ER	GREEN
TCGA-A2-A0YI	WT	WHITE	Positive	Positive	Negative	ER	GREEN
TCGA-BH-A0H3	WT	WHITE	Positive	Positive	Negative	ER	GREEN
TCGA-AC-A2FO	WT	WHITE	Positive	Negative	Indeterminate	ER	GREEN
TCGA-AC-A3BB	WT	WHITE	Positive	Positive	Negative	ER	GREEN
TCGA-A8-A08A	WT	WHITE	Positive	Positive	Negative	ER	GREEN
TCGA-E2-A1IJ	WT	WHITE	Positive	Positive	Negative	ER	GREEN
TCGA-AR-A2LM	WT	WHITE	Positive	Positive	Negative	ER	GREEN
TCGA-BH-A0EA	WT	WHITE	Positive	Positive	Negative	ER	GREEN
TCGA-E2-A15D	WT	WHITE	Positive	Positive	Negative	ER	GREEN
TCGA-EW-A1J2	WT	WHITE	Positive	Positive	Negative	ER	GREEN
TCGA-A8-A09Z	WT	WHITE	Positive	Negative	Negative	ER	GREEN
TCGA-E2-A1B4	WT	WHITE	Positive	Positive	Negative	ER	GREEN
TCGA-A1-A0SG	WT	WHITE	Positive	Positive	Negative	ER	GREEN
TCGA-BH-A0E7	WT	WHITE	Positive	Positive	Negative	ER	GREEN
TCGA-A8-A06P	WT	WHITE	Positive	Positive	Negative	ER	GREEN
TCGA-A7-A0D9	WT	WHITE	Positive	Negative	Negative	ER	GREEN
TCGA-GM-A2D9	WT	WHITE	Positive	Positive	Negative	ER	GREEN
TCGA-BH-A0H6	WT	WHITE	Positive	Positive	Negative	ER	GREEN
TCGA-BH-A0BM	WT	WHITE	Positive	Negative	Negative	ER	GREEN
TCGA-A2-A04N	WT	WHITE	Positive	Positive	Negative	ER	GREEN
TCGA-EW-A1IX	WT	WHITE	Positive	Positive	Negative	ER	GREEN

TCGA-A2-A0EW	WT	WHITE	Positive	Positive	Negative	ER	GREEN
TCGA-A2-A0T6	WT	WHITE	Positive	Positive	Negative	ER	GREEN
TCGA-E2-A1BC	WT	WHITE	Positive	Positive	Negative	ER	GREEN
TCGA-GM-A2DM	WT	WHITE	Positive	Positive	Negative	ER	GREEN
TCGA-E2-A14U	WT	WHITE	Positive	Positive	Negative	ER	GREEN
TCGA-BH-A0DQ	WT	WHITE	Positive	Positive	Negative	ER	GREEN
TCGA-A2-A0EU	WT	WHITE	Positive	Positive	Negative	ER	GREEN
TCGA-A8-A091	WT	WHITE	Positive	Negative	Negative	ER	GREEN
TCGA-E2-A153	WT	WHITE	Positive	Positive	Negative	ER	GREEN
TCGA-A2-A0CP	WT	WHITE	Positive	Positive	Negative	ER	GREEN
TCGA-E9-A227	WT	WHITE	Positive	Positive	Negative	ER	GREEN
TCGA-BH-A0W4	WT	WHITE	Positive	Positive	Negative	ER	GREEN
TCGA-BH-A0EI	WT	WHITE	Positive	Positive	Negative	ER	GREEN
TCGA-E2-A1IG	WT	WHITE	Positive	Positive	Negative	ER	GREEN
TCGA-BH-A0AZ	WT	WHITE	Positive	Positive	Negative	ER	GREEN
TCGA-E9-A1NG	WT	WHITE	Positive	Positive	Negative	ER	GREEN
TCGA-A2-A0CV	WT	WHITE	Positive	Positive	Negative	ER	GREEN
TCGA-A2-A0CS	WT	WHITE	Positive	Positive	Negative	ER	GREEN
TCGA-BH-A0EB	WT	WHITE	Positive	Positive	Negative	ER	GREEN
TCGA-B6-A1KI	WT	WHITE	Positive	Positive	Negative	ER	GREEN
TCGA-BH-A0H5	WT	WHITE	Positive	Positive	Negative	ER	GREEN
TCGA-E2-A1B5	WT	WHITE	Positive	Positive	Negative	ER	GREEN
TCGA-A2-A25A	WT	WHITE	Positive	Positive	Negative	ER	GREEN
TCGA-AO-A0JF	WT	WHITE	Positive	Positive	Negative	ER	GREEN
TCGA-A8-A07G	WT	WHITE	Positive	Positive	Negative	ER	GREEN
TCGA-A2-A1G0	WT	WHITE	Positive	Positive	Negative	ER	GREEN
TCGA-D8-A145	WT	WHITE	Positive	Positive	Negative	ER	GREEN
TCGA-D8-A141	WT	WHITE	Positive	Positive	Negative	ER	GREEN
TCGA-A2-A0EO	WT	WHITE	Positive	Positive	Negative	ER	GREEN
TCGA-BH-A0DV	WT	WHITE	Positive	Positive	Negative	ER	GREEN
TCGA-E2-A15C	WT	WHITE	Positive	Positive	Negative	ER	GREEN
TCGA-A2-A0EX	WT	WHITE	Positive	Positive	Negative	ER	GREEN
TCGA-D8-A1JH	WT	WHITE	Positive	Positive	Negative	ER	GREEN
TCGA-A8-A07J	WT	WHITE	Positive	Positive	Negative	ER	GREEN
TCGA-GI-A2C8	WT	WHITE	Positive	Positive	Negative	ER	GREEN
TCGA-A2-A259	WT	WHITE	Positive	Positive	Negative	ER	GREEN
TCGA-BH-A0BO	WT	WHITE	Positive	Positive	Negative	ER	GREEN
TCGA-AR-A24W	WT	WHITE	Positive	Positive	Negative	ER	GREEN
TCGA-EW-A1PG	WT	WHITE	Positive	Positive	Negative	ER	GREEN
TCGA-D8-A1JU	WT	WHITE	Positive	Positive	Negative	ER	GREEN
TCGA-AC-A2FK	WT	WHITE	Positive	Positive	Negative	ER	GREEN
TCGA-A2-A0ES	WT	WHITE	Positive	Positive	Negative	ER	GREEN
TCGA-A2-A0YK	WT	WHITE	Positive	Negative	Negative	ER	GREEN
TCGA-BH-A1FH	WT	WHITE	Positive	Negative	NA	ER	GREEN
TCGA-AR-A2LQ	WT	WHITE	Positive	Positive	Negative	ER	GREEN
TCGA-AC-A2FF	WT	WHITE	Positive	Positive	Negative	ER	GREEN
TCGA-B6-A0WY	WT	WHITE	Positive	Negative	NA	ER	GREEN

TCGA-D8-A27L	WT	WHITE	Positive	Positive	Negative	ER	GREEN
TCGA-A2-A0EN	WT	WHITE	Positive	Positive	Negative	ER	GREEN
TCGA-BH-A208	G187_splice	RED	NA	NA	NA	TNBC	RED
TCGA-GM-A2DI	S260fs	RED	Negative	Negative	Negative	TNBC	RED
TCGA-AO-A03U	WT	WHITE	Negative	Negative	Negative	TNBC	RED
TCGA-EW-A1P7	WT	WHITE	Negative	Negative	Negative	TNBC	RED
TCGA-AR-A2LH	WT	WHITE	Negative	Negative	Negative	TNBC	RED
TCGA-EW-A1OV	WT	WHITE	Negative	Negative	Negative	TNBC	RED
TCGA-A2-A1G6	WT	WHITE	Negative	Negative	Negative	TNBC	RED
TCGA-EW-A1P1	WT	WHITE	Negative	Negative	Negative	TNBC	RED
TCGA-OL-A5RY	H179Q	RED	NA	NA	NA	NA	WHITE
TCGA-AC-A3OD	Q144*	RED	NA	NA	NA	NA	WHITE
TCGA-PE-A5DE	M169I	RED	NA	NA	NA	NA	WHITE
TCGA-BH-A5J0	E285K	RED	NA	NA	NA	NA	WHITE
TCGA-B6-A401	R175H	RED	NA	NA	NA	NA	WHITE
TCGA-OL-A5D6	C242G	RED	NA	NA	NA	NA	WHITE
TCGA-AC-A5EH	L194R	RED	NA	NA	NA	NA	WHITE
TCGA-E9-A5UO	WT	WHITE	NA	NA	NA	NA	WHITE
TCGA-E9-A245	WT	WHITE	NA	NA	NA	NA	WHITE
TCGA-E9-A5UP	WT	WHITE	NA	NA	NA	NA	WHITE
TCGA-GM-A3NY	WT	WHITE	NA	NA	NA	NA	WHITE
TCGA-AC-A3W7	WT	WHITE	NA	NA	NA	NA	WHITE
TCGA-B6-A40B	WT	WHITE	NA	NA	NA	NA	WHITE
TCGA-E9-A54X	WT	WHITE	NA	NA	NA	NA	WHITE
TCGA-A2-A0CK	WT	WHITE	NA	NA	NA	NA	WHITE
TCGA-A7-A13H	WT	WHITE	NA	NA	NA	NA	WHITE
TCGA-AR-A1AM	WT	WHITE	NA	NA	NA	NA	WHITE
TCGA-AC-A3EH	WT	WHITE	NA	NA	NA	NA	WHITE
TCGA-MS-A51U	WT	WHITE	NA	NA	NA	NA	WHITE
TCGA-A2-A4S0	WT	WHITE	NA	NA	NA	NA	WHITE
TCGA-LL-A50Y	WT	WHITE	NA	NA	NA	NA	WHITE
TCGA-AC-A3TN	WT	WHITE	NA	NA	NA	NA	WHITE
TCGA-D8-A3Z6	WT	WHITE	NA	NA	NA	NA	WHITE
TCGA-AC-A3YJ	WT	WHITE	NA	NA	NA	NA	WHITE
TCGA-E9-A3Q9	WT	WHITE	NA	NA	NA	NA	WHITE
TCGA-A2-A3XW	WT	WHITE	NA	NA	NA	NA	WHITE
TCGA-E9-A2JT	WT	WHITE	NA	NA	NA	NA	WHITE
TCGA-E9-A1R6	WT	WHITE	NA	NA	NA	NA	WHITE
TCGA-OL-A5D8	WT	WHITE	NA	NA	NA	NA	WHITE
TCGA-LL-A5YN	WT	WHITE	NA	NA	NA	NA	WHITE
TCGA-EW-A423	WT	WHITE	NA	NA	NA	NA	WHITE
TCGA-A7-A425	WT	WHITE	NA	NA	NA	NA	WHITE
TCGA-PE-A5DD	WT	WHITE	NA	NA	NA	NA	WHITE
TCGA-B6-A408	WT	WHITE	NA	NA	NA	NA	WHITE
TCGA-AR-A2LJ	WT	WHITE	NA	NA	NA	NA	WHITE
TCGA-EW-A3E8	WT	WHITE	NA	NA	NA	NA	WHITE
TCGA-E9-A1R4	WT	WHITE	NA	NA	NA	NA	WHITE
TCGA-AC-A2FE	WT	WHITE	NA	NA	NA	NA	WHITE

TCGA-A7-A4SB	WT	WHITE	NA	NA	NA	NA	WHITE
TCGA-E2-A570	WT	WHITE	NA	NA	NA	NA	WHITE
TCGA-AR-A5QP	WT	WHITE	NA	NA	NA	NA	WHITE
TCGA-E9-A1RC	WT	WHITE	NA	NA	NA	NA	WHITE
TCGA-A2-A3KC	WT	WHITE	NA	NA	NA	NA	WHITE
TCGA-C8-A3M7	WT	WHITE	NA	NA	NA	NA	WHITE
TCGA-AR-A5QN	WT	WHITE	NA	NA	NA	NA	WHITE
TCGA-A7-A0CH	WT	WHITE	NA	NA	NA	NA	WHITE
TCGA-E9-A1R5	WT	WHITE	NA	NA	NA	NA	WHITE
TCGA-E9-A1R3	WT	WHITE	NA	NA	NA	NA	WHITE
TCGA-E9-A229	WT	WHITE	NA	NA	NA	NA	WHITE
TCGA-AQ-A54O	WT	WHITE	NA	NA	NA	NA	WHITE
TCGA-E9-A1RA	WT	WHITE	NA	NA	NA	NA	WHITE
TCGA-C8-A12Y	WT	WHITE	NA	NA	NA	NA	WHITE
TCGA-E9-A1RD	WT	WHITE	NA	NA	NA	NA	WHITE
TCGA-E9-A24A	WT	WHITE	NA	NA	NA	NA	WHITE
TCGA-E9-A1R0	WT	WHITE	NA	NA	NA	NA	WHITE
TCGA-E9-A1N3	WT	WHITE	NA	NA	NA	NA	WHITE
TCGA-AC-A3W6	WT	WHITE	NA	NA	NA	NA	WHITE
TCGA-OL-A66H	WT	WHITE	NA	NA	NA	NA	WHITE
TCGA-AR-A5QM	WT	WHITE	NA	NA	NA	NA	WHITE
TCGA-A7-A426	WT	WHITE	NA	NA	NA	NA	WHITE
TCGA-OL-A66K	WT	WHITE	NA	NA	NA	NA	WHITE
TCGA-GM-A3NW	WT	WHITE	NA	NA	NA	NA	WHITE
TCGA-A2-A4S2	WT	WHITE	NA	NA	NA	NA	WHITE
TCGA-OL-A5RX	WT	WHITE	NA	NA	NA	NA	WHITE
TCGA-GM-A3XN	WT	WHITE	NA	NA	NA	NA	WHITE
TCGA-OL-A5RV	WT	WHITE	NA	NA	NA	NA	WHITE
TCGA-B6-A0RN	WT	WHITE	Negative	Negative	NA	NA	WHITE
TCGA-A2-A0CR	WT	WHITE	NA	NA	NA	NA	WHITE
TCGA-E9-A3X8	WT	WHITE	NA	NA	NA	NA	WHITE
TCGA-A2-A4RW	WT	WHITE	NA	NA	NA	NA	WHITE
TCGA-OL-A66J	WT	WHITE	NA	NA	NA	NA	WHITE
TCGA-AC-A3HN	WT	WHITE	NA	NA	NA	NA	WHITE
TCGA-OL-A5DA	WT	WHITE	NA	NA	NA	NA	WHITE
TCGA-E9-A5FK	WT	WHITE	NA	NA	NA	NA	WHITE
TCGA-AC-A3QP	WT	WHITE	NA	NA	NA	NA	WHITE
TCGA-BH-A42U	WT	WHITE	NA	NA	NA	NA	WHITE
TCGA-GM-A3XG	WT	WHITE	NA	NA	NA	NA	WHITE
TCGA-OL-A5RU	WT	WHITE	NA	NA	NA	NA	WHITE
TCGA-D8-A3Z5	WT	WHITE	NA	NA	NA	NA	WHITE
TCGA-GM-A5PV	WT	WHITE	NA	NA	NA	NA	WHITE
TCGA-A7-A5ZX	WT	WHITE	NA	NA	NA	NA	WHITE
TCGA-GM-A5PX	WT	WHITE	NA	NA	NA	NA	WHITE
TCGA-BH-A42V	WT	WHITE	NA	NA	NA	NA	WHITE
TCGA-GM-A4E0	WT	WHITE	NA	NA	NA	NA	WHITE
TCGA-HN-A2OB	WT	WHITE	NA	NA	NA	NA	WHITE

TCGA-D8-A4Z1	WT	WHITE	NA	NA	NA	NA	WHITE
TCGA-A2-A0CO	WT	WHITE	NA	NA	NA	NA	WHITE
TCGA-A2-A4RY	WT	WHITE	NA	NA	NA	NA	WHITE
TCGA-BH-A28O	WT	WHITE	NA	NA	NA	NA	WHITE
TCGA-AC-A5XS	WT	WHITE	NA	NA	NA	NA	WHITE
TCGA-AN-A0XS	WT	WHITE	Negative	Positive	Negative	NA	WHITE
TCGA-A1-A0SH	WT	WHITE	Negative	Positive	Negative	NA	WHITE
TCGA-AC-A62Y	WT	WHITE	NA	NA	NA	NA	WHITE
TCGA-A2-A0EP	WT	WHITE	NA	NA	NA	NA	WHITE
TCGA-E9-A1RI	WT	WHITE	NA	NA	NA	NA	WHITE
TCGA-A7-A5ZW	WT	WHITE	NA	NA	NA	NA	WHITE
TCGA-AC-A3YI	WT	WHITE	NA	NA	NA	NA	WHITE
TCGA-LL-A440	WT	WHITE	NA	NA	NA	NA	WHITE
TCGA-E2-A14Y	Q136E	RED	Positive	Positive	Positive	ER/HER2	PURPLE
TCGA-C8-A130	N131fs	RED	Positive	Positive	Equivocal	ER/HER2	PURPLE
TCGA-AN-A0FJ	V157fs	RED	Positive	Negative	Positive	ER/HER2	PURPLE
TCGA-BH-A18U	E286K	RED	Positive	Positive	Positive	ER/HER2	PURPLE
TCGA-C8-A12T	T102fs	RED	Positive	Positive	Positive	ER/HER2	PURPLE
TCGA-AN-A0AJ	G108fs	RED	Positive	Positive	Positive	ER/HER2	PURPLE
TCGA-B6-A0IJ	Y220C	RED	Positive	Positive	NA	ER/HER2	PURPLE
TCGA-B6-A0I9	C135Y	RED	Indeterminate	Positive	NA	ER/HER2	PURPLE
TCGA-D8-A1XJ	R282G	RED	Positive	Positive	Positive	ER/HER2	PURPLE
TCGA-C8-A26W	R273L	RED	Positive	Positive	Equivocal	ER/HER2	PURPLE
TCGA-EW-A1OZ	E258*	RED	Positive	Negative	Positive	ER/HER2	PURPLE
TCGA-AR-A254	C124*	RED	Positive	Positive	Positive	ER/HER2	PURPLE
TCGA-A2-A0YG	T125_splice	RED	Positive	Positive	Positive	ER/HER2	PURPLE
TCGA-A7-A2KD	L93fs	RED	Positive	Positive	Positive	ER/HER2	PURPLE
TCGA-A8-A09I	WT	WHITE	Positive	Positive	Positive	ER/HER2	PURPLE
TCGA-E2-A14V	WT	WHITE	Positive	Positive	Positive	ER/HER2	PURPLE
TCGA-BH-A0DD	WT	WHITE	Positive	Positive	Positive	ER/HER2	PURPLE
TCGA-C8-A1HL	WT	WHITE	Positive	Negative	Equivocal	ER/HER2	PURPLE
TCGA-E9-A22D	WT	WHITE	Positive	Positive	Positive	ER/HER2	PURPLE
TCGA-A1-A0SN	WT	WHITE	Positive	Positive	Positive	ER/HER2	PURPLE
TCGA-B6-A0X5	WT	WHITE	Positive	Positive	NA	ER/HER2	PURPLE
TCGA-AO-A0JM	WT	WHITE	Positive	Positive	Positive	ER/HER2	PURPLE
TCGA-AN-A0AK	WT	WHITE	Positive	Negative	Positive	ER/HER2	PURPLE
TCGA-C8-A1HF	H179Y	RED	Negative	Positive	Positive	HER2	BLUE
TCGA-E2-A14P	V157F	RED	Negative	Negative	Positive	HER2	BLUE
TCGA-B6-A1KF	T125_splice	RED	Negative	Negative	Equivocal	HER2	BLUE
TCGA-A2-A04U	G108fs	RED	Negative	Negative	Positive	HER2	BLUE
TCGA-C8-A1HK	R280*	RED	Negative	Negative	Positive	HER2	BLUE
TCGA-E9-A1N9	R196*	RED	Negative	Positive	Positive	HER2	BLUE
TCGA-A2-A1G1	V225_splice	RED	Negative	Negative	Positive	HER2	BLUE
TCGA-A8-A07R	R248W	RED	Negative	Negative	Positive	HER2	BLUE
TCGA-BH-A18R	WT	WHITE	Indeterminate	Negative	Positive	HER2	BLUE
TCGA-BH-A0EE	WT	WHITE	Negative	Negative	Positive	HER2	BLUE
TCGA-E9-A22G	WT	WHITE	Negative	Negative	Positive	HER2	BLUE
TCGA-C8-A137	WT	WHITE	Negative	Negative	Positive	HER2	BLUE

TCGA-AR-A1AJ	R273H	RED	Positive	Negative	Negative	ER	GREEN
TCGA-A8-A079	S215I	RED	Positive	Positive	Negative	ER	GREEN
TCGA-AO-A03R	L265R	RED	Positive	Positive	Negative	ER	GREEN
TCGA-C8-A1HG	C141R	RED	Positive	Positive	Negative	ER	GREEN
TCGA-A2-A0CW	R196*	RED	Positive	Positive	Negative	ER	GREEN
TCGA-D8-A1Y3	R213*	RED	Positive	Positive	Negative	ER	GREEN
TCGA-C8-A1HM	L194P	RED	Positive	Positive	Negative	ER	GREEN
TCGA-AO-A0JD	H193L	RED	Positive	Positive	Negative	ER	GREEN
TCGA-AR-A251	E285K	RED	Positive	Negative	Negative	ER	GREEN
TCGA-D8-A1JM	R273H	RED	Positive	Negative	Negative	ER	GREEN
TCGA-EW-A1PC	R273C	RED	Positive	Positive	Negative	ER	GREEN
TCGA-AR-A1AH	G108fs	RED	Positive	Negative	Negative	ER	GREEN
TCGA-AN-A0FY	V173M	RED	Positive	Positive	Negative	ER	GREEN
TCGA-AO-A03O	R175H	RED	Positive	Positive	Negative	ER	GREEN
TCGA-A8-A094	R209fs	RED	Positive	Negative	Negative	ER	GREEN
TCGA-A7-A13E	R175H	RED	Positive	Negative	Negative	ER	GREEN
TCGA-AO-A03L	S261_splice	RED	Positive	Positive	Negative	ER	GREEN
TCGA-EW-A1PA	R213*	RED	Positive	Positive	Negative	ER	GREEN
TCGA-AR-A0U2	C176F	RED	Positive	Positive	Negative	ER	GREEN
TCGA-C8-A27A	G199V	RED	Positive	Positive	Negative	ER	GREEN
TCGA-BH-A0HU	WT	WHITE	Positive	Positive	Negative	ER	GREEN
TCGA-A8-A08L	WT	WHITE	Positive	Negative	Negative	ER	GREEN
TCGA-BH-A209	WT	WHITE	Positive	Positive	NA	ER	GREEN
TCGA-A8-A07L	WT	WHITE	Positive	Positive	Negative	ER	GREEN
TCGA-AR-A24Q	WT	WHITE	Positive	Negative	Negative	ER	GREEN
TCGA-AR-A24H	WT	WHITE	Positive	Positive	Negative	ER	GREEN
TCGA-BH-A1FM	WT	WHITE	Positive	Negative	NA	ER	GREEN
TCGA-C8-A12U	WT	WHITE	Positive	Positive	Negative	ER	GREEN
TCGA-AR-A0TY	WT	WHITE	Positive	Negative	Negative	ER	GREEN
TCGA-BH-A0HB	WT	WHITE	Positive	Positive	Negative	ER	GREEN
TCGA-A8-A08F	WT	WHITE	Positive	Positive	Negative	ER	GREEN
TCGA-EW-A2FV	WT	WHITE	Positive	Positive	Negative	ER	GREEN
TCGA-E2-A15S	WT	WHITE	Positive	Negative	Negative	ER	GREEN
TCGA-E2-A109	WT	WHITE	Positive	Negative	Negative	ER	GREEN
TCGA-A8-A09W	WT	WHITE	Positive	Positive	Negative	ER	GREEN
TCGA-A2-A0D4	WT	WHITE	Positive	Positive	Negative	ER	GREEN
TCGA-BH-A0C7	WT	WHITE	Positive	Negative	Negative	ER	GREEN
TCGA-D8-A1XF	WT	WHITE	Positive	Positive	Negative	ER	GREEN
TCGA-A7-A13F	WT	WHITE	Positive	Positive	Negative	ER	GREEN
TCGA-EW-A2FW	WT	WHITE	Positive	Positive	Negative	ER	GREEN
TCGA-AN-A0FW	WT	WHITE	Positive	Indeterminate	Negative	ER	GREEN
TCGA-A8-A09D	WT	WHITE	Positive	Positive	Negative	ER	GREEN
TCGA-AN-A0AM	WT	WHITE	Positive	Negative	Negative	ER	GREEN
TCGA-D8-A13Y	WT	WHITE	Positive	Positive	Negative	ER	GREEN
TCGA-A2-A25B	WT	WHITE	Positive	Positive	Negative	ER	GREEN
TCGA-A1-A0SP	S183*	RED	Negative	Negative	Negative	TNBC	RED
TCGA-EW-A1PH	K292fs	RED	Negative	Negative	Negative	TNBC	RED

TCGA-E2-A159	V216M	RED	Negative	Negative	Negative	TNBC	RED
TCGA-E2-A1B6	R342*	RED	Negative	Negative	Negative	TNBC	RED
TCGA-AO-A129	R175H	RED	Negative	Negative	Negative	TNBC	RED
TCGA-BH-A1F6	S127P	RED	Negative	Negative	Negative	TNBC	RED
TCGA-BH-A18V	E56*	RED	Negative	Negative	Negative	TNBC	RED
TCGA-AR-A1AI	F270I	RED	Negative	Negative	Negative	TNBC	RED
TCGA-A2-A0YM	R333fs	RED	Negative	Negative	Negative	TNBC	RED
TCGA-C8-A275	G266E	RED	NA	NA	NA	TNBC	RED
TCGA-A7-A0CE	T155P	RED	Negative	Negative	Negative	TNBC	RED
TCGA-BH-A0B3	V216M	RED	Negative	Negative	Negative	TNBC	RED
TCGA-AN-A0AR	E171fs	RED	Negative	Negative	Negative	TNBC	RED
TCGA-AR-A0TS	E198*	RED	Negative	Negative	Negative	TNBC	RED
TCGA-AO-A124	R213*	RED	Negative	Negative	Negative	TNBC	RED
TCGA-C8-A12L	C238F	RED	Negative	Negative	Equivocal	TNBC	RED
TCGA-E2-A1LG	I255del	RED	Negative	Negative	Negative	TNBC	RED
TCGA-A7-A26I	G266E	RED	Negative	Negative	Negative	TNBC	RED
TCGA-D8-A147	C176F	RED	Negative	Negative	Negative	TNBC	RED
TCGA-E2-A1II	Y126_splice	RED	Negative	Positive	Negative	TNBC	RED
TCGA-C8-A12K	I195T	RED	NA	NA	NA	TNBC	RED
TCGA-E2-A14N	R196*	RED	Negative	Negative	Negative	TNBC	RED
TCGA-A8-A08R	C238fs	RED	Negative	Negative	Negative	TNBC	RED
TCGA-A2-A0CM	E204fs	RED	Negative	Negative	Negative	TNBC	RED
TCGA-BH-A1FC	V225_splice	RED	Negative	Negative	Negative	TNBC	RED
TCGA-E2-A1L7	I255F	RED	Negative	Negative	Negative	TNBC	RED
TCGA-A1-A0SK	M133K	RED	Negative	Negative	Negative	TNBC	RED
TCGA-AR-A2LR	S241fs	RED	Negative	Negative	Negative	TNBC	RED
TCGA-GM-A2DH	Q331*	RED	Negative	Negative	Negative	TNBC	RED
TCGA-BH-A0B9	P278T	RED	Negative	Negative	Negative	TNBC	RED
TCGA-A7-A13D	E221*	RED	Negative	Positive	Negative	TNBC	RED
TCGA-EW-A1P4	H179R	RED	Negative	Negative	Negative	TNBC	RED
TCGA-A2-A0YE	I162fs	RED	Negative	Negative	Negative	TNBC	RED
TCGA-AO-A0J2	R175H	RED	Negative	Negative	Negative	TNBC	RED
TCGA-C8-A134	R273H	RED	Negative	Negative	Equivocal	TNBC	RED
TCGA-AR-A1AQ	R342fs	RED	Negative	Negative	Negative	TNBC	RED
TCGA-AR-A1AY	V122fs	RED	Negative	Negative	Negative	TNBC	RED
TCGA-BH-A18T	Y107D	RED	Negative	Negative	Negative	TNBC	RED
TCGA-AR-A1AR	S90fs	RED	Negative	Negative	Negative	TNBC	RED
TCGA-AR-A256	F109fs	RED	Negative	Negative	Negative	TNBC	RED
TCGA-C8-A27B	H193R	RED	Negative	Negative	Negative	TNBC	RED
TCGA-D8-A27F	S241fs	RED	Negative	Negative	Negative	TNBC	RED
TCGA-C8-A1HJ	R196*	RED	Negative	Negative	Negative	TNBC	RED
TCGA-A2-A0D0	R196*	RED	Negative	Negative	Negative	TNBC	RED
TCGA-A8-A07O	T125_splice	RED	Negative	Negative	Negative	TNBC	RED
TCGA-E2-A14R	I195T	RED	Negative	Negative	Negative	TNBC	RED
TCGA-C8-A131	R175H	RED	Negative	Negative	Negative	TNBC	RED
TCGA-BH-A0E6	C238fs	RED	Negative	Negative	Negative	TNBC	RED
TCGA-BH-A203	R273H	RED	NA	NA	NA	TNBC	RED
TCGA-AN-A0AL	R175H	RED	Negative	Negative	Negative	TNBC	RED

TCGA-E9-A1RH	E339fs	RED	NA	NA	NA	TNBC	RED
TCGA-E9-A244	R175H	RED	NA	NA	NA	TNBC	RED
TCGA-BH-A0WA	S241C	RED	Negative	Negative	Negative	TNBC	RED
TCGA-A2-A0EQ	162_163IY>N	RED	Negative	Negative	Negative	TNBC	RED
TCGA-D8-A13Z	C141Y	RED	Negative	Negative	Negative	TNBC	RED
TCGA-E9-A248	H115fs	RED	NA	NA	NA	TNBC	RED
TCGA-D8-A1JL	H193L	RED	Negative	Negative	Negative	TNBC	RED
TCGA-A7-A26F	C176F	RED	Negative	Negative	Negative	TNBC	RED
TCGA-D8-A1XQ	L252fs	RED	Negative	Negative	Negative	TNBC	RED
TCGA-AO-A0J6	T211fs	RED	Negative	Negative	Negative	TNBC	RED
TCGA-BH-A0E0	R175H	RED	Negative	Negative	Negative	TNBC	RED
TCGA-A8-A07U	F328fs	RED	Negative	Positive	Negative	TNBC	RED
TCGA-A2-A04T	R273H,D208G	RED	Negative	Negative	Negative	TNBC	RED
TCGA-EW-A1PB	WT	WHITE	Negative	Negative	Negative	TNBC	RED
TCGA-AN-A0XU	WT	WHITE	Negative	Negative	Negative	TNBC	RED
TCGA-AO-A0J4	WT	WHITE	Negative	Negative	Negative	TNBC	RED
TCGA-BH-A0AV	WT	WHITE	Negative	Negative	Negative	TNBC	RED
TCGA-E2-A1AZ	WT	WHITE	Negative	Negative	Negative	TNBC	RED
TCGA-AC-A2BK	WT	WHITE	Negative	Negative	Negative	TNBC	RED
TCGA-E2-A1LI	WT	WHITE	Negative	Negative	Negative	TNBC	RED
TCGA-EW-A1P8	WT	WHITE	Negative	Negative	Negative	TNBC	RED
TCGA-D8-A143	WT	WHITE	Negative	Negative	Negative	TNBC	RED
TCGA-E2-A1LL	WT	WHITE	Negative	Negative	Negative	TNBC	RED
TCGA-AO-A1KR	WT	WHITE	Negative	Negative	Negative	TNBC	RED
TCGA-D8-A1XK	WT	WHITE	Negative	Negative	Negative	TNBC	RED
TCGA-A2-A0D2	WT	WHITE	Negative	Negative	Negative	TNBC	RED
TCGA-AN-A04D	WT	WHITE	Negative	Negative	Negative	TNBC	RED
TCGA-AO-A12F	WT	WHITE	Negative	Negative	Negative	TNBC	RED
TCGA-E2-A1LK	WT	WHITE	Negative	Negative	Negative	TNBC	RED
TCGA-A2-A0T0	WT	WHITE	Negative	Negative	Negative	TNBC	RED
TCGA-AN-A0AT	WT	WHITE	Negative	Negative	Negative	TNBC	RED
TCGA-OL-A5RW	R209fs	RED	NA	NA	NA	NA	WHITE
TCGA-B6-A400	G108fs	RED	NA	NA	NA	NA	WHITE
TCGA-BH-A5IZ	R273H	RED	NA	NA	NA	NA	WHITE
TCGA-E9-A1RB	E286Q	RED	NA	NA	NA	NA	WHITE
TCGA-B6-A0RU	R196*	RED	Negative	Negative	NA	NA	WHITE
TCGA-E2-A573	Y220C	RED	NA	NA	NA	NA	WHITE
TCGA-AR-A5QQ	R209fs	RED	NA	NA	NA	NA	WHITE
TCGA-A2-A3Y0	R342*	RED	NA	NA	NA	NA	WHITE
TCGA-GM-A3XL	R156del	RED	NA	NA	NA	NA	WHITE
TCGA-A2-A3XT	C135R	RED	NA	NA	NA	NA	WHITE
TCGA-A7-A4SE	S261_splice	RED	NA	NA	NA	NA	WHITE
TCGA-A2-A3XX	L111P	RED	NA	NA	NA	NA	WHITE
TCGA-OL-A5D7	V272M	RED	NA	NA	NA	NA	WHITE
TCGA-B6-A0X1	Q331_splice	RED	Negative	Negative	NA	NA	WHITE
TCGA-B6-A0IQ	R213*	RED	Negative	Negative	NA	NA	WHITE
TCGA-OL-A66I	R342*	RED	NA	NA	NA	NA	WHITE
TCGA-B6-A409	L252fs	RED	NA	NA	NA	NA	WHITE

TCGA-B6-A402	R196*	RED	NA	NA	NA	NA	WHITE
TCGA-A2-A4S3	K132E	RED	NA	NA	NA	NA	WHITE
TCGA-LL-A5YO	L194R	RED	NA	NA	NA	NA	WHITE
TCGA-B6-A0RE	S303fs	RED	Negative	Negative	NA	NA	WHITE
TCGA-E2-A574	WT	WHITE	NA	NA	NA	NA	WHITE
TCGA-HN-A2NL	WT	WHITE	NA	NA	NA	NA	WHITE
TCGA-OL-A5S0	WT	WHITE	NA	NA	NA	NA	WHITE
TCGA-B6-A0I1	WT	WHITE	Negative	Negative	NA	NA	WHITE
TCGA-A2-A3XU	WT	WHITE	NA	NA	NA	NA	WHITE
TCGA-E9-A2JS	WT	WHITE	NA	NA	NA	NA	WHITE
TCGA-BH-A42T	WT	WHITE	NA	NA	NA	NA	WHITE
TCGA-E9-A228	WT	WHITE	NA	NA	NA	NA	WHITE
TCGA-B6-A0RT	WT	WHITE	Negative	Negative	NA	NA	WHITE
TCGA-E9-A3QA	WT	WHITE	NA	NA	NA	NA	WHITE
TCGA-A8-A075	G187_splice	RED	Positive	Positive	Positive	ER/HER2	PURPLE
TCGA-BH-A0C0	R110fs	RED	Positive	Positive	Positive	ER/HER2	PURPLE
TCGA-D8-A1J9	V173L	RED	Positive	Negative	Positive	ER/HER2	PURPLE
TCGA-BH-A0AW	F270S	RED	Positive	Negative	Positive	ER/HER2	PURPLE
TCGA-D8-A27N	R175H	RED	Positive	Positive	Positive	ER/HER2	PURPLE
TCGA-BH-A202	R213*	RED	Positive	Positive	Positive	ER/HER2	PURPLE
TCGA-BH-A1FN	R280T	RED	Positive	Positive	NA	ER/HER2	PURPLE
TCGA-BH-A1F2	E285K	RED	Positive	Positive	Positive	ER/HER2	PURPLE
TCGA-AQ-A04H	V197G	RED	Positive	Positive	Positive	ER/HER2	PURPLE
TCGA-A8-A06U	R209fs	RED	Positive	Positive	Positive	ER/HER2	PURPLE
TCGA-BH-A0DZ	E258Q	RED	Positive	Positive	Positive	ER/HER2	PURPLE
TCGA-BH-A0B7	G187_splice	RED	Positive	Positive	Positive	ER/HER2	PURPLE
TCGA-A8-A097	P250fs	RED	Positive	Positive	Positive	ER/HER2	PURPLE
TCGA-C8-A138	A307fs	RED	Positive	Negative	Equivocal	ER/HER2	PURPLE
TCGA-AN-A0FT	H193R	RED	Positive	Positive	Positive	ER/HER2	PURPLE
TCGA-B6-A0RH	Y220S	RED	Positive	Positive	NA	ER/HER2	PURPLE
TCGA-E2-A152	K132E	RED	Positive	Negative	Positive	ER/HER2	PURPLE
TCGA-AR-A0TX	Y205C	RED	Positive	Positive	Positive	ER/HER2	PURPLE
TCGA-AQ-A04L	H193R	RED	Positive	Negative	Positive	ER/HER2	PURPLE
TCGA-BH-A1FJ	WT	WHITE	Negative	Positive	NA	ER/HER2	PURPLE
TCGA-EW-A1J3	WT	WHITE	Positive	Positive	Positive	ER/HER2	PURPLE
TCGA-EW-A1IW	WT	WHITE	Positive	Positive	Positive	ER/HER2	PURPLE
TCGA-A8-A09G	WT	WHITE	Positive	Negative	Positive	ER/HER2	PURPLE
TCGA-A8-A07I	WT	WHITE	Positive	Negative	Positive	ER/HER2	PURPLE
TCGA-C8-A1HN	WT	WHITE	Positive	Positive	Equivocal	ER/HER2	PURPLE
TCGA-A8-A08B	WT	WHITE	Positive	Negative	Positive	ER/HER2	PURPLE
TCGA-BH-A18P	WT	WHITE	Positive	Negative	Positive	ER/HER2	PURPLE
TCGA-A8-A07B	WT	WHITE	Positive	Positive	Positive	ER/HER2	PURPLE
TCGA-D8-A1XS	WT	WHITE	Positive	Positive	Positive	ER/HER2	PURPLE
TCGA-B6-A0WV	WT	WHITE	Positive	Positive	NA	ER/HER2	PURPLE
TCGA-A2-A25E	WT	WHITE	Positive	Positive	Equivocal	ER/HER2	PURPLE
TCGA-EW-A1PD	WT	WHITE	Positive	Positive	Positive	ER/HER2	PURPLE
TCGA-BH-A0HY	WT	WHITE	Positive	Negative	Positive	ER/HER2	PURPLE
TCGA-AC-A23H	WT	WHITE	Positive	Negative	Positive	ER/HER2	PURPLE

TCGA-A2-A0CX	WT	WHITE	Positive	Negative	Positive	ER/HER2	PURPLE
TCGA-AR-A250	WT	WHITE	Positive	Negative	Positive	ER/HER2	PURPLE
TCGA-A8-A06R	WT	WHITE	Positive	Negative	Positive	ER/HER2	PURPLE
TCGA-E2-A14W	WT	WHITE	Positive	Positive	Positive	ER/HER2	PURPLE
TCGA-D8-A27W	WT	WHITE	Positive	Positive	Positive	ER/HER2	PURPLE
TCGA-A8-A06X	WT	WHITE	Positive	Negative	Positive	ER/HER2	PURPLE
TCGA-BH-A1F8	WT	WHITE	Positive	Positive	Positive	ER/HER2	PURPLE
TCGA-E9-A22H	WT	WHITE	Positive	Positive	Positive	ER/HER2	PURPLE
TCGA-B6-A0IG	WT	WHITE	Positive	Positive	NA	ER/HER2	PURPLE
TCGA-B6-A0RI	WT	WHITE	Positive	Positive	NA	ER/HER2	PURPLE
TCGA-E2-A105	WT	WHITE	Positive	Positive	Equivocal	ER/HER2	PURPLE
TCGA-AN-A0FZ	WT	WHITE	Positive	Negative	Positive	ER/HER2	PURPLE
TCGA-A2-A04X	WT	WHITE	Positive	Positive	Positive	ER/HER2	PURPLE
TCGA-B6-A0IM	WT	WHITE	Positive	Positive	NA	ER/HER2	PURPLE
TCGA-A8-A09N	WT	WHITE	Positive	Positive	Positive	ER/HER2	PURPLE
TCGA-A8-A08G	WT	WHITE	Positive	Positive	Positive	ER/HER2	PURPLE
TCGA-A8-A090	WT	WHITE	Positive	Positive	Positive	ER/HER2	PURPLE
TCGA-E9-A1NA	WT	WHITE	Positive	Positive	Positive	ER/HER2	PURPLE
TCGA-B6-A2IU	WT	WHITE	Positive	Positive	NA	ER/HER2	PURPLE
TCGA-B6-A0WW	WT	WHITE	Positive	Positive	NA	ER/HER2	PURPLE
TCGA-BH-A0B4	WT	WHITE	Positive	Positive	Positive	ER/HER2	PURPLE
TCGA-B6-A0IB	WT	WHITE	Positive	Positive	NA	ER/HER2	PURPLE
TCGA-AN-A0FD	WT	WHITE	Positive	Positive	Positive	ER/HER2	PURPLE
TCGA-E2-A15H	WT	WHITE	Positive	Positive	Positive	ER/HER2	PURPLE
TCGA-A8-A08P	WT	WHITE	Positive	Positive	Positive	ER/HER2	PURPLE
TCGA-A8-A076	WT	WHITE	Positive	Positive	Positive	ER/HER2	PURPLE
TCGA-BH-A1EV	WT	WHITE	Positive	Positive	Positive	ER/HER2	PURPLE
TCGA-A1-A0SM	WT	WHITE	Positive	Negative	Positive	ER/HER2	PURPLE
TCGA-AR-A255	WT	WHITE	Positive	Positive	Positive	ER/HER2	PURPLE
TCGA-A8-A09E	WT	WHITE	Positive	Positive	Positive	ER/HER2	PURPLE
TCGA-B6-A0IA	WT	WHITE	Positive	Positive	NA	ER/HER2	PURPLE
TCGA-AO-A0JL	R306*	RED	Negative	Negative	Positive	HER2	BLUE
TCGA-A8-A08X	C176F	RED	Negative	Negative	Positive	HER2	BLUE
TCGA-D8-A1XT	A276P	RED	Negative	Negative	Positive	HER2	BLUE
TCGA-C8-A12P	R248W	RED	Negative	Negative	Positive	HER2	BLUE
TCGA-A2-A0D1	Q331*	RED	Negative	Negative	Positive	HER2	BLUE
TCGA-C8-A135	S241fs	RED	Negative	Negative	Positive	HER2	BLUE
TCGA-AN-A0FV	L252P	RED	Negative	Negative	Positive	HER2	BLUE
TCGA-C8-A278	H168P	RED	Negative	Negative	Positive	HER2	BLUE
TCGA-AN-A0FL	V225_splice	RED	Negative	Negative	Positive	HER2	BLUE
TCGA-E9-A1ND	Y220C	RED	Negative	Negative	Positive	HER2	BLUE
TCGA-E2-A1B0	K132N	RED	Negative	Negative	Positive	HER2	BLUE
TCGA-C8-A12Z	R342P	RED	Negative	Negative	Positive	HER2	BLUE
TCGA-AN-A0FX	R248W	RED	Negative	Negative	Positive	HER2	BLUE
TCGA-C8-A12Q	Y205N	RED	Negative	Negative	Positive	HER2	BLUE
TCGA-AO-A12D	S241fs	RED	Negative	Negative	Positive	HER2	BLUE
TCGA-A2-A0T1	R273C	RED	Negative	Negative	Positive	HER2	BLUE
TCGA-EW-A2FR	R175H	RED	Negative	Negative	Positive	HER2	BLUE

TCGA-E2-A1LE	K164E	RED	Negative	Negative	Positive	HER2	BLUE
TCGA-A2-A04W	V272M	RED	Negative	Negative	Positive	HER2	BLUE
TCGA-AN-A04C	WT	WHITE	Negative	Negative	Positive	HER2	BLUE
TCGA-AR-A24U	WT	WHITE	Negative	Negative	Positive	HER2	BLUE
TCGA-E9-A1NC	WT	WHITE	Negative	Positive	Positive	HER2	BLUE
TCGA-AO-A0JE	WT	WHITE	Negative	Negative	Positive	HER2	BLUE
TCGA-D8-A1JA	WT	WHITE	Negative	Negative	Positive	HER2	BLUE
TCGA-A8-A0A7	WT	WHITE	Negative	Negative	Positive	HER2	BLUE
TCGA-BH-A1EN	WT	WHITE	Negative	Negative	Positive	HER2	BLUE
TCGA-E2-A1LB	WT	WHITE	Negative	Negative	Positive	HER2	BLUE
TCGA-A8-A092	C238Y	RED	Positive	Positive	Negative	ER	GREEN
TCGA-A2-A0SV	FLH113del	RED	Positive	Positive	Negative	ER	GREEN
TCGA-A2-A0SW	R273C	RED	Positive	Negative	Negative	ER	GREEN
TCGA-AR-A0U3	Q167fs	RED	Positive	Positive	Negative	ER	GREEN
TCGA-D8-A1JJ	C141Y	RED	Positive	Positive	Negative	ER	GREEN
TCGA-A8-A06O	G279E	RED	Positive	Positive	Negative	ER	GREEN
TCGA-A8-A06Q	C238Y	RED	Positive	Positive	Negative	ER	GREEN
TCGA-A8-A07W	H179R	RED	Positive	Positive	Negative	ER	GREEN
TCGA-A2-A0YT	I255S	RED	Positive	Negative	Negative	ER	GREEN
TCGA-AR-A24K	F109fs	RED	Positive	Positive	Negative	ER	GREEN
TCGA-A7-A0CJ	R175H	RED	Positive	Positive	Negative	ER	GREEN
TCGA-C8-A12W	H193Y	RED	Positive	Positive	Negative	ER	GREEN
TCGA-A2-A0YH	P278L	RED	Positive	Positive	Negative	ER	GREEN
TCGA-EW-A1OY	Q192*	RED	Positive	Positive	Negative	ER	GREEN
TCGA-D8-A1XL	R248W	RED	Positive	Positive	Negative	ER	GREEN
TCGA-AO-A0JB	R213*	RED	Positive	Positive	Negative	ER	GREEN
TCGA-A8-A081	C141Y	RED	Positive	Positive	Negative	ER	GREEN
TCGA-E2-A155	S33_splice	RED	Positive	Negative	Negative	ER	GREEN
TCGA-AR-A24S	D281V	RED	Positive	Positive	Negative	ER	GREEN
TCGA-E2-A15M	L130V	RED	Positive	Positive	Negative	ER	GREEN
TCGA-BH-A0GY	R175H	RED	Positive	Positive	Negative	ER	GREEN
TCGA-A8-A08I	Y163C	RED	Positive	Positive	Negative	ER	GREEN
TCGA-BH-A0DL	E286A	RED	Positive	Negative	Negative	ER	GREEN
TCGA-A2-A0YJ	R342*	RED	Positive	Negative	Negative	ER	GREEN
TCGA-A8-A06Z	P128fs	RED	Positive	Positive	Negative	ER	GREEN
TCGA-E2-A2P5	F341S	RED	Positive	Positive	Negative	ER	GREEN
TCGA-BH-A0BR	V274F	RED	Positive	Positive	Negative	ER	GREEN
TCGA-BH-A1FD	V173L	RED	Positive	Positive	Negative	ER	GREEN
TCGA-GM-A2DL	D281E	RED	Positive	Positive	Negative	ER	GREEN
TCGA-C8-A12O	R273H	RED	Positive	Positive	Negative	ER	GREEN
TCGA-AR-A24P	Y234C	RED	Positive	Positive	Negative	ER	GREEN
TCGA-A1-A0SI	R175H	RED	Positive	Positive	Negative	ER	GREEN
TCGA-AO-A03N	A161T	RED	Positive	Positive	Negative	ER	GREEN
TCGA-C8-A26V	I232S	RED	Positive	Positive	Negative	ER	GREEN
TCGA-D8-A1XZ	Q167*	RED	Positive	Negative	Negative	ER	GREEN
TCGA-BH-A0HX	C135W	RED	Positive	Positive	Negative	ER	GREEN
TCGA-BH-A0BD	WT	WHITE	Positive	Positive	Negative	ER	GREEN
TCGA-BH-A0H0	WT	WHITE	Positive	Positive	Negative	ER	GREEN

TCGA-D8-A1J8	WT	WHITE	Positive	Positive	Negative	ER	GREEN
TCGA-AN-A03Y	WT	WHITE	Positive	Positive	Negative	ER	GREEN
TCGA-A2-A0T3	WT	WHITE	Positive	Positive	Negative	ER	GREEN
TCGA-A8-A095	WT	WHITE	Positive	Positive	Negative	ER	GREEN
TCGA-A8-A07E	WT	WHITE	Positive	Positive	Negative	ER	GREEN
TCGA-AR-A2LK	WT	WHITE	Positive	Positive	Negative	ER	GREEN
TCGA-D8-A1JD	WT	WHITE	Positive	Positive	Negative	ER	GREEN
TCGA-AN-A0XW	WT	WHITE	Positive	Positive	Negative	ER	GREEN
TCGA-BH-A0E1	WT	WHITE	Positive	Positive	Negative	ER	GREEN
TCGA-BH-A0E2	WT	WHITE	Positive	Positive	Negative	ER	GREEN
TCGA-E9-A22A	WT	WHITE	Positive	Negative	Negative	ER	GREEN
TCGA-E2-A15A	WT	WHITE	Positive	Positive	Negative	ER	GREEN
TCGA-BH-A18F	WT	WHITE	Positive	Positive	Negative	ER	GREEN
TCGA-A8-A084	WT	WHITE	Positive	Negative	Negative	ER	GREEN
TCGA-BH-A0B5	WT	WHITE	Positive	Positive	Negative	ER	GREEN
TCGA-A7-A26J	WT	WHITE	Positive	Positive	Negative	ER	GREEN
TCGA-E2-A10A	WT	WHITE	Positive	Positive	Negative	ER	GREEN
TCGA-A2-A1FX	WT	WHITE	Positive	Positive	Negative	ER	GREEN
TCGA-AR-A24L	WT	WHITE	Positive	Positive	Negative	ER	GREEN
TCGA-BH-A0AU	WT	WHITE	Positive	Positive	Negative	ER	GREEN
TCGA-AR-A24R	WT	WHITE	Positive	Positive	Negative	ER	GREEN
TCGA-E2-A15R	WT	WHITE	Positive	Positive	Negative	ER	GREEN
TCGA-BH-A0BZ	WT	WHITE	Positive	Positive	Negative	ER	GREEN
TCGA-AO-A03M	WT	WHITE	Positive	Positive	Negative	ER	GREEN
TCGA-AR-A1AO	WT	WHITE	Positive	Negative	Negative	ER	GREEN
TCGA-A2-A04R	WT	WHITE	Positive	Positive	Negative	ER	GREEN
TCGA-D8-A1JC	WT	WHITE	Positive	Positive	Negative	ER	GREEN
TCGA-D8-A1Y0	WT	WHITE	Positive	Positive	Negative	ER	GREEN
TCGA-AO-A1KS	WT	WHITE	Positive	Positive	Negative	ER	GREEN
TCGA-AO-A1KT	WT	WHITE	Positive	Positive	Negative	ER	GREEN
TCGA-A2-A0CL	WT	WHITE	Positive	Positive	Negative	ER	GREEN
TCGA-BH-A18L	WT	WHITE	Positive	Positive	Negative	ER	GREEN
TCGA-A2-A1FW	WT	WHITE	Positive	Negative	Negative	ER	GREEN
TCGA-A2-A25C	WT	WHITE	Positive	Positive	Negative	ER	GREEN
TCGA-E2-A1LA	WT	WHITE	Positive	Positive	Negative	ER	GREEN
TCGA-D8-A27R	WT	WHITE	Positive	Positive	Negative	ER	GREEN
TCGA-D8-A1JT	WT	WHITE	Positive	Positive	Negative	ER	GREEN
TCGA-AR-A24N	WT	WHITE	Positive	Positive	Negative	ER	GREEN
TCGA-AR-A24Z	WT	WHITE	Positive	Positive	Negative	ER	GREEN
TCGA-AO-A0J7	WT	WHITE	Positive	Positive	Negative	ER	GREEN
TCGA-E2-A10C	WT	WHITE	Positive	Positive	Negative	ER	GREEN
TCGA-E2-A107	WT	WHITE	Positive	Negative	Negative	ER	GREEN
TCGA-E2-A15O	WT	WHITE	Positive	Positive	Negative	ER	GREEN
TCGA-AN-A0XR	WT	WHITE	Positive	Negative	Negative	ER	GREEN
TCGA-A8-A082	WT	WHITE	Positive	Positive	Negative	ER	GREEN
TCGA-E2-A14S	WT	WHITE	Positive	Positive	Negative	ER	GREEN
TCGA-GM-A2DO	WT	WHITE	Positive	Positive	Negative	ER	GREEN
TCGA-A2-A04V	WT	WHITE	Positive	Positive	Negative	ER	GREEN

TCGA-AO-A03T	WT	WHITE	Positive	Positive	Negative	ER	GREEN
TCGA-C8-A273	WT	WHITE	Positive	Positive	Negative	ER	GREEN
TCGA-A8-A07Z	WT	WHITE	Positive	Positive	Negative	ER	GREEN
TCGA-BH-A0W3	WT	WHITE	Positive	Positive	Negative	ER	GREEN
TCGA-E2-A14Z	WT	WHITE	Positive	Positive	Negative	ER	GREEN
TCGA-AO-A12E	WT	WHITE	Positive	Positive	Negative	ER	GREEN
TCGA-E2-A14T	WT	WHITE	Positive	Positive	Negative	ER	GREEN
TCGA-A2-A1G4	WT	WHITE	Positive	Positive	Negative	ER	GREEN
TCGA-C8-A1HO	WT	WHITE	Positive	Positive	Negative	ER	GREEN
TCGA-E2-A1IE	WT	WHITE	Positive	Positive	Negative	ER	GREEN
TCGA-A2-A0YF	WT	WHITE	Positive	Negative	Negative	ER	GREEN
TCGA-A8-A09M	WT	WHITE	Positive	Positive	Negative	ER	GREEN
TCGA-A8-A085	WT	WHITE	Positive	Positive	Negative	ER	GREEN
TCGA-E2-A15T	WT	WHITE	Positive	Positive	Negative	ER	GREEN
TCGA-A2-A0ER	WT	WHITE	Positive	Positive	Negative	ER	GREEN
TCGA-E2-A2P6	WT	WHITE	Positive	Positive	Negative	ER	GREEN
TCGA-A8-A09R	WT	WHITE	Positive	Positive	Negative	ER	GREEN
TCGA-D8-A1XM	WT	WHITE	Positive	Positive	Negative	ER	GREEN
TCGA-A2-A0EV	WT	WHITE	Positive	Positive	Negative	ER	GREEN
TCGA-E9-A1NE	WT	WHITE	Positive	Positive	Negative	ER	GREEN
TCGA-BH-A0HK	WT	WHITE	Positive	Negative	Negative	ER	GREEN
TCGA-D8-A1Y1	WT	WHITE	Positive	Positive	Negative	ER	GREEN
TCGA-E9-A1NI	WT	WHITE	Positive	Positive	Negative	ER	GREEN
TCGA-A2-A0T4	WT	WHITE	Positive	Positive	Negative	ER	GREEN
TCGA-AO-A1KP	WT	WHITE	Positive	Positive	Negative	ER	GREEN
TCGA-A2-A04Y	WT	WHITE	Positive	Positive	Negative	ER	GREEN
TCGA-D8-A1JP	WT	WHITE	Positive	Positive	Negative	ER	GREEN
TCGA-AO-A0J3	WT	WHITE	Positive	Positive	Negative	ER	GREEN
TCGA-BH-A0DK	WT	WHITE	Positive	Positive	Negative	ER	GREEN
TCGA-A2-A25D	WT	WHITE	Positive	Negative	Negative	ER	GREEN
TCGA-C8-A274	WT	WHITE	Positive	Positive	Negative	ER	GREEN
TCGA-C8-A26Z	WT	WHITE	Positive	Positive	Negative	ER	GREEN
TCGA-A1-A0SJ	WT	WHITE	Positive	Positive	Negative	ER	GREEN
TCGA-A8-A08J	WT	WHITE	Positive	Negative	Negative	ER	GREEN
TCGA-A8-A09Q	WT	WHITE	Positive	Positive	Negative	ER	GREEN
TCGA-B6-A1KC	WT	WHITE	Positive	Negative	Negative	ER	GREEN
TCGA-AN-A049	WT	WHITE	Positive	Positive	Negative	ER	GREEN
TCGA-EW-A1IY	WT	WHITE	Positive	Positive	Negative	ER	GREEN
TCGA-AN-A04A	WT	WHITE	Positive	Positive	Negative	ER	GREEN
TCGA-AO-A0JA	WT	WHITE	Positive	Positive	Negative	ER	GREEN
TCGA-A8-A09C	WT	WHITE	Positive	Positive	Negative	ER	GREEN
TCGA-D8-A1X9	WT	WHITE	Positive	Positive	Negative	ER	GREEN
TCGA-A8-A09A	WT	WHITE	Positive	Positive	Negative	ER	GREEN
TCGA-A2-A0CT	WT	WHITE	Positive	Negative	Negative	ER	GREEN
TCGA-E9-A22B	WT	WHITE	Positive	Negative	Negative	ER	GREEN
TCGA-E2-A14O	WT	WHITE	Positive	Positive	Negative	ER	GREEN
TCGA-AC-A2BM	WT	WHITE	Positive	Positive	Negative	ER	GREEN
TCGA-A8-A086	WT	WHITE	Positive	Positive	Negative	ER	GREEN

TCGA-A2-A0D3	WT	WHITE	Positive	Positive	Negative	ER	GREEN
TCGA-EW-A1IZ	WT	WHITE	Positive	Positive	Negative	ER	GREEN
TCGA-EW-A2FS	WT	WHITE	Positive	Negative	Negative	ER	GREEN
TCGA-A2-A0CU	WT	WHITE	Positive	Positive	Negative	ER	GREEN
TCGA-D8-A1Y2	WT	WHITE	Positive	Positive	Negative	ER	GREEN
TCGA-B6-A0IN	WT	WHITE	Positive	Negative	NA	ER	GREEN
TCGA-AO-A03V	WT	WHITE	Positive	Positive	Negative	ER	GREEN
TCGA-AR-A2LN	WT	WHITE	Positive	Positive	Negative	ER	GREEN
TCGA-EW-A1J6	WT	WHITE	Positive	Positive	Negative	ER	GREEN
TCGA-A7-A0DA	I195T	RED	Negative	Negative	Negative	TNBC	RED
TCGA-E9-A1N8	T256fs	RED	Negative	NA	Negative	TNBC	RED
TCGA-A2-A0SX	G199V	RED	Negative	Negative	Negative	TNBC	RED
TCGA-E2-A1LH	K132T	RED	Negative	Negative	Negative	TNBC	RED
TCGA-GM-A2DF	V225_splice	RED	Negative	Negative	Negative	TNBC	RED
TCGA-A2-A04P	G108fs	RED	Negative	Negative	Negative	TNBC	RED
TCGA-BH-A1FU	R175H	RED	Negative	Negative	NA	TNBC	RED
TCGA-D8-A1JK	Q331_splice	RED	Negative	Positive	Negative	TNBC	RED
TCGA-AO-A128	R342*	RED	Negative	Negative	Negative	TNBC	RED
TCGA-AC-A2QH	D281Y	RED	Negative	Negative	Negative	TNBC	RED
TCGA-A8-A09X	M246I	RED	Negative	Negative	Negative	TNBC	RED
TCGA-D8-A142	P278A	RED	Negative	Negative	Negative	TNBC	RED
TCGA-D8-A27M	R196*	RED	Negative	Negative	Negative	TNBC	RED
TCGA-E9-A243	W91*,Y220S	RED	NA	NA	NA	TNBC	RED
TCGA-BH-A0BL	P151H	RED	Negative	Negative	Negative	TNBC	RED
TCGA-D8-A1JG	L289F	RED	Negative	Negative	Negative	TNBC	RED
TCGA-E2-A150	C176*	RED	Negative	Negative	Negative	TNBC	RED
TCGA-BH-A0RX	R342*	RED	Negative	Negative	Negative	TNBC	RED
TCGA-BH-A0BG	L201fs	RED	Negative	Negative	Negative	TNBC	RED
TCGA-A7-A26G	Y220H	RED	Negative	Negative	Negative	TNBC	RED
TCGA-BH-A1F0	V147fs	RED	Negative	Indeterminate	Negative	TNBC	RED
TCGA-E2-A14X	R213*	RED	Negative	Negative	Negative	TNBC	RED
TCGA-BH-A18Q	A76fs	RED	Negative	Negative	Negative	TNBC	RED
TCGA-AQ-A04J	L111P	RED	Negative	Negative	Negative	TNBC	RED
TCGA-GM-A2DB	E294*	RED	Negative	Negative	Negative	TNBC	RED
TCGA-A2-A0T2	Y220C	RED	Negative	Negative	Negative	TNBC	RED
TCGA-C8-A26X	V173M	RED	Negative	Negative	Negative	TNBC	RED
TCGA-D8-A1JF	H179R	RED	Negative	Negative	Negative	TNBC	RED
TCGA-D8-A1XW	Y126N	RED	Negative	Positive	Negative	TNBC	RED
TCGA-GM-A2DD	R175H	RED	Negative	Negative	Negative	TNBC	RED
TCGA-E2-A158	I195T	RED	Negative	Negative	Negative	TNBC	RED
TCGA-D8-A27H	WT	WHITE	Negative	Negative	Negative	TNBC	RED
TCGA-C8-A26Y	WT	WHITE	Negative	Negative	Negative	TNBC	RED
TCGA-A2-A04Q	WT	WHITE	Negative	Negative	Negative	TNBC	RED
TCGA-BH-A0BW	WT	WHITE	Negative	Negative	Negative	TNBC	RED
TCGA-A2-A25F	WT	WHITE	Negative	Positive	Negative	TNBC	RED
TCGA-A2-A0ST	WT	WHITE	Negative	Negative	Negative	TNBC	RED
TCGA-C8-A12V	WT	WHITE	Negative	Negative	Negative	TNBC	RED
TCGA-AC-A2QJ	WT	WHITE	Negative	Negative	Negative	TNBC	RED

TCGA-BH-A18G	WT	WHITE	Negative	Negative	Negative	TNBC	RED
TCGA-BH-A1EW	WT	WHITE	Negative	Negative	Negative	TNBC	RED
TCGA-A2-A3XS	S241fs	RED	NA	NA	NA	NA	WHITE
TCGA-EW-A3U0	E204*	RED	NA	NA	NA	NA	WHITE
TCGA-E9-A5FL	H193R	RED	NA	NA	NA	NA	WHITE
TCGA-B6-A0IK	L194R	RED	Negative	Negative	NA	NA	WHITE
TCGA-A2-A3XY	G187_splice	RED	NA	NA	NA	NA	WHITE
TCGA-LL-A5YP	R280K	RED	NA	NA	NA	NA	WHITE
TCGA-A7-A4SD	M237I	RED	NA	NA	NA	NA	WHITE
TCGA-AC-A62X	I195T	RED	NA	NA	NA	NA	WHITE
TCGA-AR-A0U0	L194R	RED	Negative	Negative	NA	NA	WHITE
TCGA-GI-A2C9	C176Y	RED	NA	NA	NA	NA	WHITE
TCGA-B6-A3ZX	V216M	RED	NA	NA	NA	NA	WHITE
TCGA-B6-A0WX	L114*,R248W	RED	Negative	Negative	NA	NA	WHITE
TCGA-B6-A1KN	L265P	RED	Negative	Negative	NA	NA	WHITE
TCGA-AC-A5XU	R342P	RED	NA	NA	NA	NA	WHITE
TCGA-A7-A56D	T304fs	RED	NA	NA	NA	NA	WHITE
TCGA-AQ-A54N	R306*	RED	NA	NA	NA	NA	WHITE
TCGA-A2-A3XV	C135F	RED	NA	NA	NA	NA	WHITE
TCGA-B6-A0IE	A307_splice	RED	Negative	Negative	NA	NA	WHITE
TCGA-A7-A4SA	P151A	RED	NA	NA	NA	NA	WHITE
TCGA-JL-A3YW	E51*	RED	NA	NA	NA	NA	WHITE
TCGA-AN-A0XN	Y126_splice	RED	Negative	Positive	Negative	NA	WHITE
TCGA-A7-A5ZV	T211fs	RED	NA	NA	NA	NA	WHITE
TCGA-E9-A3HO	WT	WHITE	NA	NA	NA	NA	WHITE
TCGA-LL-A5YM	WT	WHITE	NA	NA	NA	NA	WHITE
TCGA-E9-A1RG	WT	WHITE	NA	NA	NA	NA	WHITE
TCGA-OL-A5RZ	WT	WHITE	NA	NA	NA	NA	WHITE
TCGA-E9-A1RF	WT	WHITE	NA	NA	NA	NA	WHITE
TCGA-E9-A247	WT	WHITE	NA	NA	NA	NA	WHITE
TCGA-LL-A441	WT	WHITE	NA	NA	NA	NA	WHITE
TCGA-PE-A5DC	WT	WHITE	NA	NA	NA	NA	WHITE
TCGA-E9-A226	WT	WHITE	NA	NA	NA	NA	WHITE
TCGA-BH-A204	WT	WHITE	NA	NA	NA	NA	WHITE
TCGA-B6-A0RG	WT	WHITE	Negative	Negative	NA	NA	WHITE
TCGA-A7-A4SF	WT	WHITE	NA	NA	NA	NA	WHITE
TCGA-E2-A56Z	WT	WHITE	NA	NA	NA	NA	WHITE
TCGA-JL-A3YX	WT	WHITE	NA	NA	NA	NA	WHITE
TCGA-AC-A3W5	WT	WHITE	NA	NA	NA	NA	WHITE
TCGA-B6-A40C	WT	WHITE	NA	NA	NA	NA	WHITE
TCGA-LQ-A4E4	WT	WHITE	NA	NA	NA	NA	WHITE
TCGA-AC-A3TM	WT	WHITE	NA	NA	NA	NA	WHITE
TCGA-LL-A5YL	WT	WHITE	NA	NA	NA	NA	WHITE
TCGA-BH-A2L8	WT	WHITE	NA	NA	NA	NA	WHITE
TCGA-AC-A2FM	WT	WHITE	NA	NA	NA	NA	WHITE
TCGA-C8-A3M8	WT	WHITE	NA	NA	NA	NA	WHITE
TCGA-A2-A3XZ	WT	WHITE	NA	NA	NA	NA	WHITE
TCGA-OK-A5Q2	WT	WHITE	NA	NA	NA	NA	WHITE

TCGA-A2-A4RX	WT	WHITE	NA	NA	NA	NA	WHITE
TCGA-E9-A249	WT	WHITE	NA	NA	NA	NA	WHITE
TCGA-E9-A1QZ	WT	WHITE	NA	NA	NA	NA	WHITE
TCGA-E9-A1RE	WT	WHITE	NA	NA	NA	NA	WHITE
TCGA-B6-A0I2	WT	WHITE	NA	NA	NA	NA	WHITE
TCGA-E9-A1R7	WT	WHITE	NA	NA	NA	NA	WHITE

Reference List

(1) Chen S, Chen Y, Hu C, Jing H, Cao Y, Liu X. Association of clinicopathological features with UbcH10 expression in colorectal cancer. *J Cancer Res Clin Oncol* 2010 March;136(3):419-26.

(2) Jiang L, Bao Y, Luo C et al. Knockdown of ubiquitin-conjugating enzyme E2C/UbcH10 expression by RNA interference inhibits glioma cell proliferation and enhances cell apoptosis in vitro. *J Cancer Res Clin Oncol* 2010 February;136(2):211-7.

(3) Shimo A, Tanikawa C, Nishidate T et al. Involvement of kinesin family member 2C/mitotic centromere-associated kinesin overexpression in mammary carcinogenesis. *Cancer Sci* 2008 January;99(1):62-70.

(4) Nakamura Y, Tanaka F, Haraguchi N et al. Clinicopathological and biological significance of mitotic centromere-associated kinesin overexpression in human gastric cancer. *Br J Cancer* 2007 August 20;97(4):543-9.

(5) Yim EK, Tong SY, Ho EM, Bae JH, Um SJ, Park JS. Anticancer effects on TACC3 by treatment of paclitaxel in HPV-18 positive cervical carcinoma cells. *Oncol Rep* 2009 February;21(2):549-57.

(6) Zhang SH, Xu AM, Chen XF, Li DH, Sun MP, Wang YJ. Clinicopathologic significance of mitotic arrest defective protein 2 overexpression in hepatocellular carcinoma. *Hum Pathol* 2008 December;39(12):1827-34.

(7) Shang X, Burlingame SM, Okcu MF et al. Aurora A is a negative prognostic factor and a new therapeutic target in human neuroblastoma. *Mol Cancer Ther* 2009 August;8(8):2461-9.

(8) Inoda S, Hirohashi Y, Torigoe T et al. Cep55/c10orf3, a tumor antigen derived from a centrosome residing protein in breast carcinoma. *J Immunother* 2009 June;32(5):474-85.

(9) Chen CH, Chien CY, Huang CC et al. Expression of FLJ10540 is correlated with aggressiveness of oral cavity squamous cell carcinoma by stimulating cell migration and invasion through increased FOXM1 and MMP-2 activity. *Oncogene* 2009 July 30;28(30):2723-37.

(10) Zheng H, Hu W, Deavers MT et al. Nuclear cyclin B1 is overexpressed in low-malignant-potential ovarian tumors but not in epithelial ovarian cancer. *Am J Obstet Gynecol* 2009 October;201(4):367-6.

(11) de HT, Hasselt N, Troost D et al. Molecular risk stratification of medulloblastoma patients based on immunohistochemical analysis of MYC, LDHB, and CCNB1 expression. *Clin Cancer Res* 2008 July 1;14(13):4154-60.

(12) Zhang K, Hu S, Wu J et al. Overexpression of RRM2 decreases thrombspondin-1 and increases VEGF production in human cancer cells in vitro and in vivo: implication of RRM2 in angiogenesis. *Mol Cancer* 2009;8:11.

(13) Duxbury MS, Ito H, Zinner MJ, Ashley SW, Whang EE. RNA interference targeting the M2 subunit of ribonucleotide reductase enhances pancreatic adenocarcinoma chemosensitivity to gemcitabine. *Oncogene* 2004 February 26;23(8):1539-48.

(14) Boukovinas I, Papadaki C, Mendez P et al. Tumor BRCA1, RRM1 and RRM2 mRNA expression levels and clinical response to first-line gemcitabine plus docetaxel in non-small-cell lung cancer patients. *PLoS One* 2008;3(11):e3695.

(15) Zhao L, Qin LX, Ye QH et al. KIAA0008 gene is associated with invasive phenotype of human hepatocellular carcinoma--a functional analysis. *J Cancer Res Clin Oncol* 2004 December;130(12):719-27.

(16) Szponar A, Zubakov D, Pawlak J, Jauch A, Kovacs G. Three genetic developmental stages of papillary renal cell tumors: duplication of chromosome 1q marks fatal progression. *Int J Cancer* 2009 May 1;124(9):2071-6.

(17) Tsunoda N, Kokuryo T, Oda K et al. Nek2 as a novel molecular target for the treatment of breast carcinoma. *Cancer Sci* 2009 January;100(1):111-6.

(18) Xiao GF, Tang HH. [Expression and clinical significance of highly expressed protein in cancer (Hec 1) in human primary gallbladder carcinoma]. *Xi Bao Yu Fen Zi Mian Yi Xue Za Zhi* 2008 September;24(9):910-2.

(19) Chen MF, Lee KD, Lu MS et al. The predictive role of E2-EPF ubiquitin carrier protein in esophageal squamous cell carcinoma. *J Mol Med* 2009 March;87(3):307-20.

(20) Park SH, Yu GR, Kim WH, Moon WS, Kim JH, Kim DG. NF-Y-dependent cyclin B2 expression in colorectal adenocarcinoma. *Clin Cancer Res* 2007 February 1;13(3):858-67.

(21) Taniuchi K, Nakagawa H, Nakamura T et al. Down-regulation of RAB6KIFL/KIF20A, a kinesin involved with membrane trafficking of discs large homologue 5, can attenuate growth of pancreatic cancer cell. *Cancer Res* 2005 January 1;65(1):105-12.

(22) Kang JU, Koo SH, Kwon KC, Park JW, Kim JM. Gain at chromosomal region 5p15.33, containing TERT, is the most frequent genetic event in early stages of non-small cell lung cancer. *Cancer Genet Cytogenet* 2008 April 1;182(1):1-11.

(23) Jiang R, Xia Y, Li J et al. High expression levels of IKKalpha and IKKbeta are necessary for the malignant properties of liver cancer. *Int J Cancer* 2010 March 1;126(5):1263-74.

(24) Mitra A, Jameson C, Barbachano Y et al. Overexpression of RAD51 occurs in aggressive prostatic cancer. *Histopathology* 2009 December;55(6):696-704.

(25) Dankof A, Fritzsche FR, Dahl E et al. KPNA2 protein expression in invasive breast carcinoma and matched peritumoral ductal carcinoma in situ. *Virchows Arch* 2007 November;451(5):877-81.

(26) Naoe M, Ogawa Y, Morita J et al. Expression of the fluoropyrimidine-metabolizing enzymes in bladder cancers as measured by the Danenberg tumor profile. *Oncol Res* 2009;18(4):153-62.

(27) Seo J, Chung YS, Sharma GG et al. Cdt1 transgenic mice develop lymphoblastic lymphoma in the absence of p53. *Oncogene* 2005 December 8;24(55):8176-86.

(28) Singh P, Yang M, Dai H et al. Overexpression and hypomethylation of flap endonuclease 1 gene in breast and other cancers. *Mol Cancer Res* 2008 November;6(11):1710-7.

(29) Arai M, Kondoh N, Imazeki N et al. The knockdown of endogenous replication factor C4 decreases the growth and enhances the chemosensitivity of hepatocellular carcinoma cells. *Liver Int* 2009 January;29(1):55-62.

(30) Karanikolas BD, Figueiredo ML, Wu L. Comprehensive evaluation of the role of EZH2 in the growth, invasion, and aggression of a panel of prostate cancer cell lines. *Prostate* 2010 May 1;70(6):675-88.

(31) Sugiura T, Nagano Y, Noguchi Y. DDX39, upregulated in lung squamous cell cancer, displays RNA helicase activities and promotes cancer cell growth. *Cancer Biol Ther* 2007 June;6(6):957-64.

(32) Lee H, Kim D, Dan HC et al. Identification and characterization of putative tumor suppressor NGB, a GTP-binding protein that interacts with the neurofibromatosis 2 protein. *Mol Cell Biol* 2007 March;27(6):2103-19.

(33) Ooe A, Kato K, Noguchi S. Possible involvement of CCT5, RGS3, and YKT6 genes up-regulated in p53-mutated tumors in resistance to docetaxel in human breast cancers. *Breast Cancer Res Treat* 2007 March;101(3):305-15.

(34) Karmakar S, Foster EA, Smith CL. Estradiol downregulation of the tumor suppressor gene BTG2 requires estrogen receptor-alpha and the REA corepressor. *Int J Cancer* 2009 April 15;124(8):1841-51.

(35) Cheng CJ, Lin YC, Tsai MT et al. SCUBE2 suppresses breast tumor cell proliferation and confers a favorable prognosis in invasive breast cancer. *Cancer Res* 2009 April 15;69(8):3634-41.

(36) Wang Y, Ma Y, Lu B, Xu E, Huang Q, Lai M. Differential expression of mimecan and thioredoxin domain-containing protein 5 in colorectal adenoma and cancer: a proteomic study. *Exp Biol Med (Maywood)* 2007 October;232(9):1152-9.

(37) Majid SM, Liss AS, You M, Bose HR. The suppression of SH3BGRL is important for v-Rel-mediated transformation. *Oncogene* 2006 February 2;25(5):756-68.

(38) Shubbar E, Kovacs A, Hajizadeh S et al. Elevated cyclin B2 expression in invasive breast carcinoma is associated with unfavorable clinical outcome. *BMC Cancer* 2013;13:1.

(39) Pollok S, Bauerschmidt C, Sanger J, Nasheuer HP, Grosse F. Human Cdc45 is a proliferation-associated antigen. *FEBS J* 2007 July;274(14):3669-84.

(40) Takahashi S, Fusaki N, Ohta S et al. Downregulation of KIF23 suppresses glioma proliferation. *J Neurooncol* 2012 February;106(3):519-29.

(41) Sircar K, Huang H, Hu L et al. Mitosis phase enrichment with identification of mitotic centromere-associated kinesin as a therapeutic target in castration-resistant prostate cancer. *PLoS One* 2012;7(2):e31259.

(42) Shimo A, Nishidate T, Ohta T, Fukuda M, Nakamura Y, Katagiri T. Elevated expression of protein regulator of cytokinesis 1, involved in the growth of breast cancer cells. *Cancer Sci* 2007 February;98(2):174-81.

(43) Zhao ZK, Wu WG, Chen L et al. Expression of UbcH10 in pancreatic ductal adenocarcinoma and its correlation with prognosis. *Tumour Biol* 2013 June;34(3):1473-7.

(44) Fristrup N, Birkenkamp-Demtroder K, Reinert T et al. Multicenter validation of cyclin D1, MCM7, TRIM29, and UBE2C as prognostic protein markers in non-muscle-invasive bladder cancer. *Am J Pathol* 2013 February;182(2):339-49.

(45) Wang WY, Hsu CC, Wang TY et al. A gene expression signature of epithelial tubulogenesis and a role for ASPM in pancreatic tumor progression. *Gastroenterology* 2013 November;145(5):1110-20.

(46) Jiang BY, Zhang XC, Su J et al. BCL11A overexpression predicts survival and relapse in non-small cell lung cancer and is modulated by microRNA-30a and gene amplification. *Mol Cancer* 2013;12:61.

(47) Sun XP, Dong X, Lin L et al. Up-regulation of survivin by AKT and hypoxia-inducible factor 1alpha contributes to cisplatin resistance in gastric cancer. *FEBS J* 2014 January;281(1):115-28.

(48) Hayama S, Daigo Y, Yamabuki T et al. Phosphorylation and activation of cell division cycle associated 8 by aurora kinase B plays a significant role in human lung carcinogenesis. *Cancer Res* 2007 May 1;67(9):4113-22.

(49) Mi YJ, Gao J, Xie JD et al. Prognostic relevance and therapeutic implications of centromere protein F expression in patients with esophageal squamous cell carcinoma. *Dis Esophagus* 2013 August;26(6):636-43.

(50) Dai Y, Liu L, Zeng T et al. Characterization of the oncogenic function of centromere protein F in hepatocellular carcinoma. *Biochem Biophys Res Commun* 2013 July 12;436(4):711-8.

(51) Mascareno EJ, Belashov I, Siddiqui MA, Liu F, Dhar-Mascareno M. Hexim-1 modulates androgen receptor and the TGF-beta signaling during the progression of prostate cancer. *Prostate* 2012 June 15;72(9):1035-44.

(52) Stypula-Cyrus Y, Mutyal NN, Dela CM et al. End-binding protein 1 (EB1) up-regulation is an early event in colorectal carcinogenesis. *FEBS Lett* 2014 March 3;588(5):829-35.

(53) Zhang G, Zhang Z, Liu Z. Polo-like kinase 1 is overexpressed in renal cancer and participates in the proliferation and invasion of renal cancer cells. *Tumour Biol* 2013 June;34(3):1887-94.

(54) Hardy S, Wong NN, Muller WJ, Park M, Tremblay ML. Overexpression of the protein tyrosine phosphatase PRL-2 correlates with breast tumor formation and progression. *Cancer Res* 2010 November 1;70(21):8959-67.

(55) Demeure MJ, Coan KE, Grant CS et al. PTTG1 overexpression in adrenocortical cancer is associated with poor survival and represents a potential therapeutic target. *Surgery* 2013 December;154(6):1405-16.

(56) Ke B, Wu LL, Liu N, Zhang RP, Wang CL, Liang H. Overexpression of stathmin 1 is associated with poor prognosis of patients with gastric cancer. *Tumour Biol* 2013 October;34(5):3137-45.

(57) Sonego M, Schiappacassi M, Lovisa S et al. Stathmin regulates mutant p53 stability and transcriptional activity in ovarian cancer. *EMBO Mol Med* 2013 May;5(5):707-22.

(58) Khurana A, McKean H, Kim H et al. Silencing of HSulf-2 expression in MCF10DCIS.com cells attenuate ductal carcinoma in situ progression to invasive ductal carcinoma in vivo. *Breast Cancer Res* 2012;14(2):R43.

(59) Hao J, Xu A, Xie X et al. Elevated expression of UBE2T in lung cancer tumors and cell lines. *Tumour Biol* 2008;29(3):195-203.

(60) Ueki T, Park JH, Nishidate T et al. Ubiquitination and downregulation of BRCA1 by ubiquitin-conjugating enzyme E2T overexpression in human breast cancer cells. *Cancer Res* 2009 November 15;69(22):8752-60.

(61) Liu Z, Ling K, Wu X et al. Reduced expression of cenp-e in human hepatocellular carcinoma. *J Exp Clin Cancer Res* 2009;28:156.

(62) Hauser S, Ulrich T, Wurster S, Schmitt K, Reichert N, Gaubatz S. Loss of LIN9, a member of the DREAM complex, cooperates with SV40 large T antigen to induce genomic instability and anchorage-independent growth. *Oncogene* 2012 April 5;31(14):1859-68.

(63) Huang CJ, Yang SH, Lee CL, Cheng YC, Tai SY, Chien CC. Ribosomal protein S27-like in colorectal cancer: a candidate for predicting prognoses. *PLoS One* 2013;8(6):e67043.

(64) He H, Sun Y. Ribosomal protein S27L is a direct p53 target that regulates apoptosis. *Oncogene* 2007 April 26;26(19):2707-16.

(65) Doolan P, Clynes M, Kennedy S et al. TMEM25, REPS2 and Meis 1: favourable prognostic and predictive biomarkers for breast cancer. *Tumour Biol* 2009;30(4):200-9.
